# Integration of positive environmental factors and differentiation of parental figures in social exposome research

**DOI:** 10.3389/fpsyg.2025.1655172

**Published:** 2025-10-27

**Authors:** Valeria Lavín, Pilar Torrecilla, Thomas R. Kwapil, Neus Barrantes-Vidal

**Affiliations:** ^1^Departament de Psicologia Clínica i de la Salut, Universitat Autònoma de Barcelona, Barcelona, Spain; ^2^Department of Psychology, University of Illinois at Urbana-Champaign, Champaign, IL, United States; ^3^CIBER de Salud Mental, Instituto de Salud Carlos III, Madrid, Spain

**Keywords:** exposome, positive experiences, adversity, psychopathology, well-being, resilience, risk

## Abstract

**Background:**

Early adversity is well established as a risk factor for poor mental health, but the potential protective role of positive experiences has been scarcely examined. The exposome paradigm provides a comprehensive framework to model the full spectrum of early environmental experiences, capturing both general and specific dimensions of these experiences. This study aimed to (i) develop an Early Social Exposome score integrating positive and negative experiences, and (ii) explore its associations with positive and negative outcomes.

**Methods:**

Early environmental experiences, psychopathology, positive outcomes, and functioning were assessed for 1,181 non-clinical young adults. Iterative exploratory factor analyses were conducted to optimize the modeling of environmental variables. A final Bifactor Confirmatory Factor Analysis was applied to obtain factor scores.

**Results:**

A general score and four specific factors emerged: Positive Experiences, Paternal Adversity, Maternal Adversity, and Role Reversal. The general Early Exposome was associated with higher psychopathology and lower positive mental health and functioning, whereas Positive Experiences showed the opposite pattern. Maternal Adversity was associated with psychopathology, whereas Paternal Adversity, mirroring epidemiological findings, showed a modest relationship with poorer functioning.

**Conclusion:**

Findings highlight the importance of assessing and integrating positive experiences in exposome research when modeling the early social environment. Its inclusion allowed to capture the significant protective role of positive peer experiences, which probably partially accounts for the heterogeneity of outcomes related to adversity exposures. Additionally, the figure or source of childhood experiences emerged as a relevant factor that should be contemplated in future research along with the nature of experiences.

## 1 Introduction

Early childhood is a critical period marked by heightened sensitivity to environmental influences. These formative years encapsulate a diverse spectrum of experiences that have profound and enduring effects on mental health (Shonkoff and Phillips, 2000). This malleability to environmental inputs is driven by rapid neurodevelopment and increased brain plasticity that characterizes this sensitive period (Kolb et al., 2013).

Adverse Childhood Experiences (ACE) can alter brain development, influencing neural circuits involved in threat detection, emotional regulation and reward processing (Teicher et al., 2016; Samson et al., 2024) and have long-lasting effects on psychological and social functioning (Pfaltz et al., 2022; Sheinbaum et al., 2024; Heinonen et al., 2018). ACE are associated with increased risk for many psychopathology expressions (Hostinar et al., 2023; McLaughlin et al., 2020; Pietrek et al., 2013), including depression (Humphreys et al., 2020), anxiety (Bandoli et al., 2017), behavioral disorders (Ballard et al., 2015) and psychosis (Sideli et al., 2020; Catalan et al., 2017). The high comorbidity rates across mental health disorders (McGrath et al., 2020) led to the identification of a broad, superordinate factor of general psychopathology (Snyder and Hankin, 2017; Smith et al., 2020), often called the P-factor (Caspi et al., 2014). This concept suggests a common dimension contributing to the interconnectedness of mental health symptoms and facilitates the examination of common risk and protective factors across diagnostic categories (Carver et al., 2017). However, whether a single dimension can capture the complexity and diversity of psychopathological variation is debated (Watts et al., 2024).

In contrast to ACE, research has scarcely examined the role of positive childhood experiences (PCE; e.g., supportive relationships, safe environments) in shaping mental health outcomes (Narayan et al., 2018; Masten, 2006). PCE not only buffer the detrimental impact of ACE, fostering resilience and well-being (Bethell et al., 2019; Redican et al., 2023), but independently contribute to improved adult health and reduced risk of mental and physical infirmity (Huang et al., 2023).

The operationalization and empirical study of early environments have largely focused on adversity (Lacey and Minnis, 2020). *Specificity models* focus on the individual effects of specific adversities (Cecil et al., 2017), whereas the *cumulative risk* models posit that multiple adversities have additive effects on developmental outcomes (Evans et al., 2013). Both approaches have limitations: specificity models fail to account for interplay and co-occurrence of individual adversities, whereas cumulative risk approaches lack precision in detailing mechanisms through which adversities influence development (McLaughlin et al., 2021).

Dimensional models have emerged as an alternative and encompass theory-driven and empirically-driven methods (Lacey and Minnis, 2020). Theory-driven models, such as the Dimensional Model of Adversity and Psychopathology (McLaughlin and Sheridan, 2016), aim to identify core underlying dimensions across types of adversities likely affecting developmental processes similarly (McLaughlin et al., 2021). Though gaining empirical support (e.g., Miller et al., 2018; Schäfer et al., 2023), this approach also has limitations, as some adversity subtypes do not fit into these dimensions or overlap across categories (Smith and Pollak, 2021). Empirically-driven methods, like factor analysis, group adversities based on correlations to derive dimensions (Lacey and Minnis, 2020) and offer explanatory power in investigating associations with several phenotypes (Brumley et al., 2019). Recent research supports combining approaches, revealing that different early experiences seem to specifically influence certain developmental processes, while also contributing to a general and cumulative vulnerability that impacts the expression of psychopathology (McGinnis et al., 2022; Gizdic et al., 2023).

Recently, the exposome paradigm has gained prominence in epidemiological research. This innovative approach advocates for the comprehensive integration of all environmental exposures experienced across a lifetime (Wild, 2005; Guloksuz et al., 2018). Exposome scores—aggregate weighted scores of environmental exposures—predict general mental health and functioning (Erzin and Guloksuz, 2021; Moore et al., 2022; Barzilay et al., 2022; Pries et al., 2022). Apart from this overarching score, the exposome framework also accounts for the specificity of environmental subdomains, and specific factors comprising the exposome have also been modeled (Pries et al., 2022).

While advances in exposome research have significantly improved our understanding of environmental exposures and their health effects—for instance, through major initiatives that map complex environmental exposures across the lifespan (ISGlobal, 2020; ISGlobal, 2021)—much of this work has focused on biological (e.g., endocrine disruptors, Warkentin et al., 2022), chemical (e.g., air pollution, Dominguez et al., 2023), and physical exposures (e.g., urban environment studies, Khomenko et al., 2023), leaving social and structural factors underrepresented (Gudi-Mindermann et al., 2023). Moreover, exposome research lacks studies examining both positive and negative experiences. Assessing positive experiences allows studying their potential role fostering resilience and favorable outcomes (Thakkar et al., 2023) and may better account for heterogenous outcomes associated with adversity.

### 1.1 The present study

This study employs an exposome framework analysis (Moore et al., 2022; Guloksuz et al., 2018) integrating a wide range of *both* adverse and positive early experiences. Furthermore, the study explores the associations of the early social exposome and its dimensions with both subclinical trans-syndromic psychopathology dimensions and positive mental health outcomes.

The study has two specific goals. First, to develop a comprehensive early exposome factor integrating adverse and positive experiences. Following Moore et al. (2022), we employed iterative factor analyses aimed at optimizing data dimensionality and tested the goodness of fit of a bifactor model encompassing both a general factor (i.e., exposome) and specific factors. Second, we examined the associations between the derived early exposome factor and its dimensions with three outcome domains—subclinical psychopathology, positive mental health, and general and social functioning.

Note that the exploratory nature of the analyses precluded making specific hypotheses regarding the number and content of the factor analysis-derived dimensions. However, we expected that elevated levels of adversity, as reflected in the general factor, would be associated with more symptoms, worse functioning, and lower levels of positive mental health, whereas higher levels of positive experiences would be related to fewer symptoms, enhanced functioning, and greater positive mental health indicators.

## 2 Methods

### 2.1 Participants

The study sample was composed of 1,181 non-clinically ascertained young adults (*M* age = 22.8 years, *SD* = 6.5, range = 18–62 years, 76% female) recruited from two universities in Barcelona. Breakdown of socioeconomic status was 3% low, 10.8% middle-low, 31.5% middle class, 61.2% middle-high, and 3.5% high. Initially, 1,220 participants completed the measures; however, 39 participants were excluded due to invalid responding, defined by a score greater than 3 on the Infrequency Scale (Chapman and Chapman, 1983). Recruitment was conducted via posters and emails sent to students and university staff and took place between 16 December 2022 and 29 March 2023. Exclusion criteria included: (a) being under 18 years old, and (b) having grandparents of non-Spanish origin to maintain ancestry homogeneity for genetic analyses used in the project. Written informed consent was obtained from all participants. The study was approved (ref. 5,426) by the Ethics Committee of the Universitat Autònoma de Barcelona (Comissió d’Ètica en l’Experimentació Animal i Humana).

### 2.2 Materials and procedure

See Supplementary material for a detailed description of the measures.

#### 2.2.1 Environmental measures

A total of 145 items reflecting a multidimensional exploration of early social environment were included in the analysis. These variables encompass positive and negative experiences from early childhood, including school dynamics, intrafamilial relationships, peer interactions, neighborhood characteristics, and financial status. The items were drawn from established self-report scales, including the Childhood Trauma Questionnaire-Brief (Bernstein et al., 2003), the Childhood Experiences of Care and Abuse Questionnaire-3 Brief (Li et al., 2020), the Parental Bonding Instrument (PBI; Parker et al., 1979), the Benevolent Childhood Experiences Scale (Narayan et al., 2018), the Positive Childhood Experiences Scale (Bethell et al., 2019) and 4 items assessing family-level protective factors derived by Crush et al. (2018).

#### 2.2.2 Phenotypic measures

Schizotypy traits and experiences were assessed with the Multidimensional Schizotypy Scale-Brief (MSS-B; Gross et al., 2018), the Schizotypal Personality Questionnaire (SPQ; Raine, 1991) and the Community Assessment of Psychic Experiences (CAPE; Stefanis et al., 2002). Depressive symptoms with the Beck Depression Inventory (BDI; Beck et al., 1979) and anxiety with the Beck Anxiety Inventory (BAI; Beck et al., 1988).

Positive mental health was assessed using the Rosenberg Self-Esteem Scale (Rosenberg, 1965), the Connor-Davidson Resilience Scale (Campbell-Sills and Stein, 2007) and the Warwick-Edinburgh Mental Wellbeing Scale (Tennant et al., 2007).

Functioning was assessed with the Social Functioning Questionnaire (Tyrer et al., 2005).

### 2.3 Statistical analysis

#### 2.3.1 Modeling of early environment and generation of factor scores

All 145 items from the early environmental measures were included in the analysis (Supplementary Table S1). Given the large number of variables, diverse formats (ordinal, continuous, and binary), and the data’s multidimensional yet interconnected nature, a complex data reduction process was conducted following Moore et al. (2022). Subsequently, the underlying factorial structure of the remaining items was entered into a Bifactor Confirmatory Factor Analysis (BCFA). See Supplementary methods for a detailed description of the process.

#### 2.3.2 Modeling of psychopathology, positive mental health, and functioning outcome measures and determination of factor scores

We derived an integrative P-factor score capturing transdiagnostic vulnerability to subclinical psychopathology including psychosis-spectrum, depressive, and anxiety features, as well as an analogous general factor score tapping positive mental health indicators such as well-being, resilience, and self-esteem. For each indicator, an EFA was conducted on all measures comprising it, and the resulting factorial structure was then used in a BCFA to compute a composite score.

#### 2.3.3 Associations of early exposome factor scores with outcome variables

Bivariate correlations and linear regression analysis were computed to test the association of the Early Exposome and its dimensions with the P-factor, Positive Mental Health index, functioning, and individual measures of psychopathology and positive mental health. In the regression analysis all predictors were entered simultaneously to examine their unique contribution. Bootstrap procedures with 2000 samples were used for regression models. The standardized regression coefficients (*β*) and effect sizes (*f*^2^) are reported for each predictor in the linear regressions.

## 3 Results

### 3.1 Results of the environmental data reduction

Descriptive statistics, reliability and correlations for study variables are displayed in Supplementary Tables S2, S3. A total of five EFAs were conducted (Supplementary Tables S4–S7 and Table 1) until a final solution was obtained, retaining 99 of the initial 145 variables for subsequent analysis. All items were included in the initial EFA (Supplementary Table S4). Items from measures that separate the mother/father figure clustered together within the same factors, whereas general items that did not specify parental figures grouped similarly. This prompted running two EFAs to analyze these two sets of items independently (Supplementary Tables S5, S6) and then we conducted a fourth EFA (Supplementary Table S7) including all remaining items and subscales from all measures.

**Table 1:** Factor loadings of the final exploratory factor analysis of the optimized collection of early exposome items using iterative target rotation (*n* = 1.181).

Item	Content	FAC1 Positive experiences	FAC2 Paternal adversity	FAC3 Maternal adversity	FAC4 Role reversal
PCE4_R	Sense of belonging in high school	**0.97**	0.19	0.25	0.09
BCE_8	Opportunities to have a good time	**0.92**	0.07	0.35	−0.04
PCE5_R	Felt supported by friends	**0.91**	0.13	0.32	0.08
Support2R	Peers to discuss problems and feelings	**0.89**	0.10	0.35	0.09
BCE2_R	At least one good friend	**0.87**	0.18	0.33	0.07
BCE_42R	Liked high school	**0.86**	0.12	0.28	0.06
PCE3_R	Enjoyed community traditions	**0.79**	−0.02	0.15	0.02
BCE7_R	Adult who provided support or advice	**0.70**	0.01	0.14	0.06
PCE7_R	Felt safe and protected by an adult at home	**0.68**	−0.08	−0.03	−0.23
PCE1_R	Capable of discussing feelings with family	**0.68**	−0.05	−0.06	0.12
PCE2_R	Supported by family during tough times	**0.68**	−0.05	−0.13	0.01
BCE5_R	At least one teacher that cared	**0.67**	0.00	0.30	0.06
BCE_9	Like or felt comfortable with oneself	**0.66**	0.00	0.13	−0.04
CTQ_7	Felt loved	**0.62**	−0.19	−0.13	−0.11
PCE6_R	At least two caring adults (not parents)	**0.62**	−0.07	0.07	−0.03
BCE_41R	Liked school	**0.59**	−0.02	0.19	−0.06
CTQ_5	Family member made me feel valued	**0.58**	−0.07	−0.17	0.07
CTQ_2	Family member took care of me	**0.58**	−0.04	0.01	−0.23
BCE1_R	Caregiver provided sense of safety	**0.56**	−0.04	0.06	0.02
FAMILYPROT2	Ways to have fun at home	**0.51**	−0.19	−0.02	−0.26
CTQ_29	Family was source of strength	**0.49**	−0.23	−0.18	−0.17
Support1R	Adults could go to with problems/discuss feelings	**0.49**	−0.16	−0.19	0.02
CTQ_20	Family felt close	**0.44**	−0.21	−0.04	−0.22
FAMILYPROT3	Lived in a happy home	**0.40**	−0.29	−0.05	−0.37
CARE_P1	Spoke in a warm and friendly voice	0.11	**−0.79**	0.04	0.02
PA_P4	Humiliated me, put me down.	−0.02	**0.78**	0.04	0.16
ANTIPATHY_F2	Was critical of me	−0.17	**0.77**	0.00	−0.01
ANTIPATHY_F1	Was difficult to please	−0.12	**0.75**	0.02	0.03
PA_P6	Was rejecting	−0.05	**0.74**	0.11	0.10
ANTIPATHY_F3	Made me feel I was a nuisance	−0.11	**0.74**	0.05	0.12
CARE_P5	Enjoyed talking things over with me	0.15	**−0.73**	−0.04	0.16
CARE_P6	Frequently smiled at me	0.10	**−0.72**	−0.09	0.10
PA_P1	Undermined my confidence	−0.06	**0.71**	0.00	0.08
PA_P2	Played on my fears	0.00	**0.69**	0.07	0.16
ANTIPATHY_F4	Picked on me unfairly	−0.08	**0.69**	−0.02	0.15
CARE_P9	Emotionally cold to me	−0.13	**0.69**	0.11	−0.11
PA_P8	Made me feel guilty, so I would do what I was told	0.16	**0.68**	0.12	0.29
CARE_P11	Made me feel I wasn’t wanted	−0.22	**0.67**	−0.04	0.02
CARE_P4	Was affectionate to me	0.12	**−0.67**	−0.09	0.09
CARE_P12	Did not praise me	−0.22	**0.66**	0.06	−0.16
CARE_P8	Did not talk with me very much	−0.13	**0.66**	0.10	−0.13
CARE_P10	Did not understand what I needed or wanted	−0.23	**0.65**	0.06	0.00
CARE_P3	Understood my problems and worries	0.23	**−0.64**	−0.04	0.08
PA_P3	Liked to see me suffer	−0.13	**0.63**	−0.07	0.12
CARE_P7	Made me feel better when I was upset	0.15	**−0.62**	−0.04	0.07
PA_P7	Took away things I cherished	−0.09	**0.57**	0.04	0.17
PA_P5	Shamed me in front of others	−0.06	**0.54**	0.06	0.11
PBI_Fat_OVERPR	Subscale score	0.02	**0.49**	0.09	0.05
ANTIPATHY_F6	Did not like me as much as my siblings	−0.24	**0.45**	0.07	−0.10
CARE_P2	Did not help me as much as I needed	−0.23	**0.45**	0.09	−0.02
ANTIPATHY_F5	Was there if I needed	−0.19	**0.32**	−0.09	−0.08
ANTIPATHY_M3	Was critical	−0.03	−0.01	**0.82**	0.08
ANTIPATHY_M1	Was difficult to please	−0.05	0.05	**0.74**	0.07
ANTIPATHY_M4	Made me feel I was a nuisance	−0.07	0.06	**0.74**	0.19
PA_M4	Humiliated me, put me down	0.02	0.02	**0.74**	0.20
PA_M1	Undermined my confidence	0.04	0.13	**0.73**	0.03
ANTIPATHY_M5	Picked on me unfairly	0.03	−0.07	**0.70**	0.25
CARE_M5	Enjoyed talking things over with me	0.18	−0.01	**−0.69**	0.11
CARE_M1	Spoke in a warm and friendly voice	0.15	0.01	**−0.68**	−0.01
PA_M8	Made me feel guilty, so I would do what I was told	0.04	0.06	**0.68**	0.14
CARE_M9	Emotionally cold to me	−0.13	0.00	**0.67**	0.02
PA_M3	Played on my fears	0.00	0.07	**0.67**	0.13
CARE_M11	Made me feel I wasn’t wanted	−0.25	−0.02	**0.67**	0.03
ANTIPATHY_M2	Made me feel unwanted	−0.27	−0.13	**0.66**	0.08
PA_M6	Was rejecting	−0.20	−0.05	**0.66**	0.05
CARE_M4	Was affectionate to me	0.16	−0.04	**−0.64**	0.00
CARE_M3	Understood my problems and worries	0.24	−0.01	**−0.64**	0.03
CARE_M10	Did not understand what I needed or wanted	−0.21	0.08	**0.63**	−0.02
CARE_M6	Frequently smiled at me	0.23	−0.01	**−0.60**	0.01
CARE_M12	Did not praise me	−0.23	0.08	**0.59**	−0.09
PA_M2	Confuse me by telling contradictory things	0.00	0.09	**0.56**	0.13
PBI_Mot_OVERPR	Subscale score	0.09	0.17	**0.56**	−0.02
CARE_M8	Did not talk with me very much	−0.24	0.01	**0.56**	−0.07
PA_M5	Shamed me in front of others	−0.05	0.05	**0.54**	0.12
CARE_M7	Made me feel better when I was upset	0.17	0.01	**−0.53**	0.08
PA_M9	Threatened to hurt loved ones to get what she wanted	0.02	−0.26	**0.52**	0.37
ANTIPATHY_M6	Did not like me as much as my siblings	−0.21	−0.04	**0.50**	0.06
PA_M11	Said wanted me dead	−0.23	−0.16	**0.45**	0.38
CARE_M2	Not helped me as much as I needed	−0.22	0.06	**0.42**	0.12
PA_M7	Deprivation of light, food, or company	0.04	0.11	**0.41**	0.37
CTQ_3	Family called me things like “stupid,” “lazy” or “ugly”	−0.16	0.27	**0.31**	0.23
RR_5R	Parent/s look to you for help as a child	0.02	−0.04	0.03	**0.61**
RR_1R	Lot of responsibility at home	−0.06	0.04	0.06	**0.59**
CTQ_1	Not enough food to eat	−0.17	−0.04	0.02	**0.58**
RR_6R	Parent/s confided their problems in you	0.13	−0.03	−0.03	**0.58**
RR_9R	Felt concerned and worried about parents	0.00	0.09	−0.04	**0.56**
RR_2R	Expected to do a lot of housework	−0.09	0.02	0.11	**0.51**
CECA_Discord	Subscale score	−0.06	0.22	0.13	**0.51**
CTQ_4	Parents too drunk or to high	−0.10	0.13	−0.12	**0.51**
CTQ_6	Wore dirty clothes	−0.21	−0.11	−0.07	**0.50**
RR_8R	Parent/s rely for emotional support as a child	0.01	0.15	0.15	**0.50**
RR_4R	Missed out seeing friends because of home responsibilities	−0.04	0.09	0.14	**0.48**
BCE10_R	Had a predictable home routine	0.38	−0.01	0.09	**−0.44**
RR_3R	Had to look after younger siblings	0.00	0.04	0.13	**0.43**
CECA_Violence	Subscale score	0.06	0.12	−0.03	**0.43**
FAMILYPROT4	Home overly chaotic or noisy	0.10	−0.13	−0.09	**−0.39**
CTQ_26	Was emotionally abused	−0.21	0.27	0.21	**0.38**
CTQ_14	Family said hurful or insulting things	−0.09	0.32	0.20	**0.37**
CTQ_Physical_Abuse	Subscale score	−0.04	0.17	0.19	**0.33**

Inter-factor correlations resulting from EFA				
	F1 Positive	F2 Father	F3 Mother	F4 General Adv.
FAC 1	–			
FAC 2	−0.52	–		
FAC 3	−0.63	0.29	–	
FAC 4	−0.32	0.15	0.23	–

Highest factor loadings for a given factor are bolded; Cross loadings are underlined; CTQ, Childhood Trauma Questionnaire; RR, role reversal; CECA, childhood experiences of care and abuse; PA, psychological abuse; PCE, positive childhood experiences; BCE, benevolent childhood experiences.

The concluding EFA employing iterated target rotation (ITR; Moore et al., 2015) revealed a four-factor solution (Table 1), as indicated by the scree plot in Supplementary Figure S1. Factor 1 encompassed extra-familiar positive experiences and peer support (“Positive Experiences”). Factor 2 included negative experiences or lack of care related to the paternal figure (“Paternal Adversity”). Factor 3 covered similar experiences as Factor 2 but pertaining to the maternal figure (“Maternal Adversity”). Factor 4 (“Role Reversal”) included items related to role reversal and some low-endorsed items related to other adverse experiences, such as parental violence and physical neglect.

**Figure 1:**
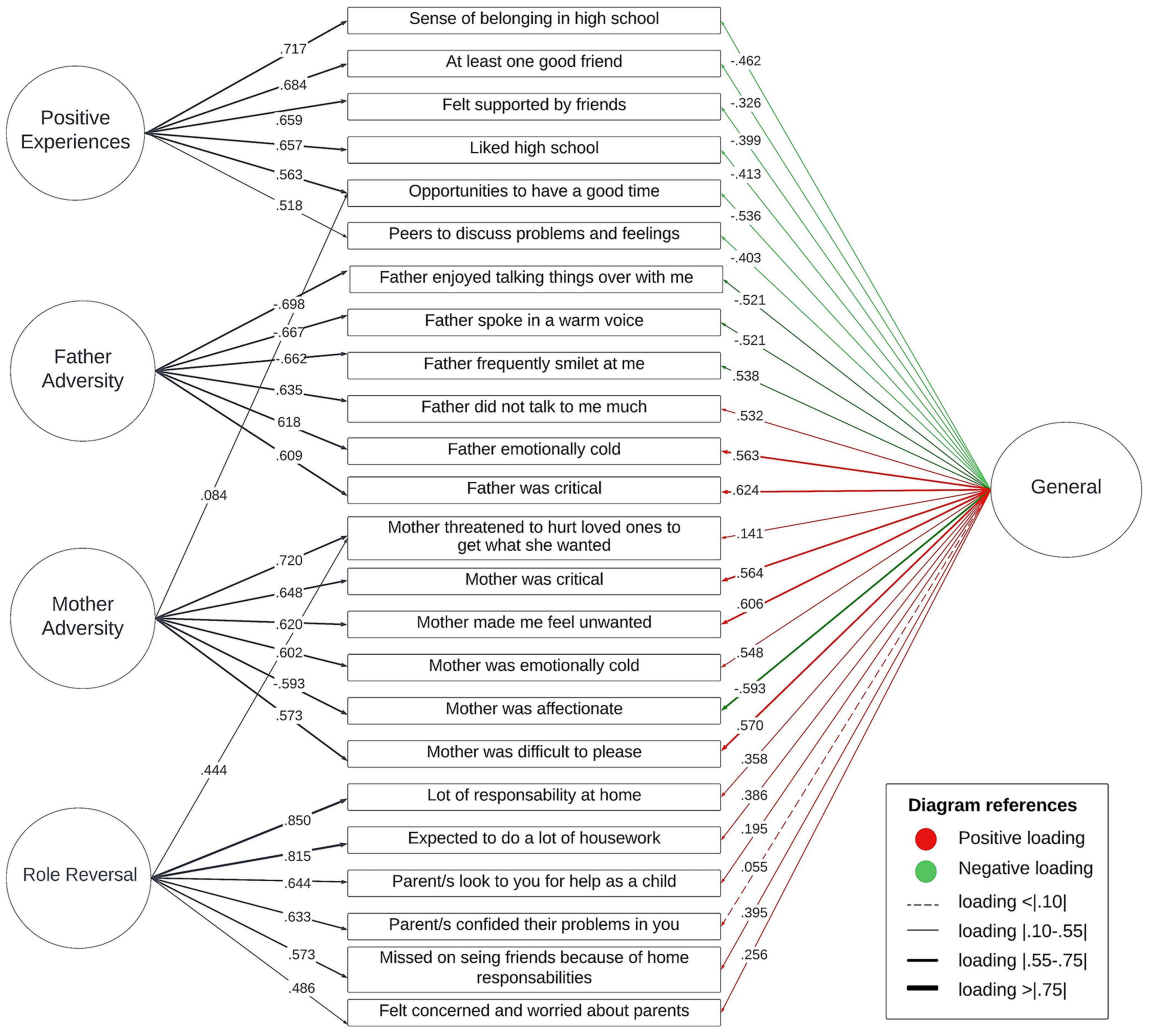
Early exposome bifactor model. Note that only the top six items with the highest loadings in the specific factors are presented.

The subsequent BCFA (see Figure 1 and Supplementary Table S8) demonstrated good global fit (CFI = 0.97, TLI = 0.97, SRMR = 0.06, and RMSEA = 0.05). All factors exhibited high reliability (*ω* = 0.86–0.96; *H* = 0.90–0.98). Supplementary Table S9 shows the complete model-based reliability results.

The general factor accounted for 56.7% of the ECV, indicating a strong general factor while also suggesting multidimensionality. Specific factors exhibited varying proportions of ECV, lower than the general factor [mean ECV-SG of 10.83% (range: 6.1–14.9%)], representing a contribution to the model in terms of explaining specific aspects or subdomains within the broader construct measured by the general factor.

### 3.2 Derivation of a P-factor score

An EFA using ITR revealed a four-factor structure with some cross-loadings (Supplementary Table S11). The subsequent BCFA (Supplementary Table S12) presented good model fit (CFI = 0.95, TLI = 0.94, RMSEA = 0.047, SRMR = 0.066; Supplementary Table S13 shows additional bifactor fit indices). A general P-factor plus four distinct factors were identified: Affective Dysregulation, Social/Cognitive Malfunctioning, Positive Schizotypy, and Negative Schizotypy.

### 3.3 Derivation of a positive mental health index

Given the expectation of a simple structure, an EFA with oblimin rotation was conducted. Items clustered by measure, yielding a three-factor solution devoid of cross-loadings (Supplementary Table S13). The subsequent BCFA (Supplementary Table S14) exhibited excellent fit (CFI = 0.97; TLI = 0.97; SRMR = 0.044; RMSEA = 0.087; Supplementary Table S15 shows additional bifactor fit indices).

### 3.4 Associations of the early exposome and its dimensions with outcome domains and individual scales

Table 2 presents the linear regression analyses examining the Early Exposome and its dimensions as predictors of the P-factor, the Positive Mental Health index and functioning, as well as the individual psychopathology and positive mental health measures. Bivariate correlations are presented in Supplementary Tables S16, S17.

**Table 2:** Linear regressions examining prediction of the main outcome domains and individual (P-factor, a Positive Mental Health Index and functioning) and individual measures by the Early Exposome and its dimensions (*n* = 1,181).

Criteria	Regression model																	
	Early exposome			F1 Positive experiences			F2 Paternal adversity			F3 Maternal adversity			F4 Role reversal			Total effect		
	*β*	*p*	*f* ^2^	*β*	*p*	*f* ^2^	*β*	*p*	*f* ^2^	*β*	*p*	*f* ^2^	*β*	*p*	*f* ^2^	*R* ^2^	*p*	*f* ^2^
Main outcome domains																		
P-factor	0.401	0.000	**0.20**	−0.171	0.000	0.04	0.043	0.097	0.00	0.100	0.000	0.01	0.041	0.113	0.00	0.220	0.000	**0.28**
Positive Mental Health	−0.367	0.000	**0.16**	0.155	0.000	0.03	−0.022	0.417	0.00	−0.043	0.104	0.00	0.056	0.036	0.00	0.173	0.000	**0.21**
Functioning	−0.484	0.000	**0.32**	0.188	0.000	0.05	−0.062	0.013	0.01	−0.028	0.267	0.00	0.021	0.400	0.00	0.291	0.000	***0.41***
Individual measures																		
Psychosis extended phenotype																		
MSS Positive	0.161	0.000	0.03	−0.033	0.242	0.00	0.015	0.598	0.00	0.084	0.004	0.01	0.119	0.000	0.01	0.053	0.000	0.06
MSS Negative	0.222	0.000	0.05	−0.148	0.000	0.02	−0.017	0.552	0.00	0.026	0.366	0.00	−0.002	0.946	0.00	0.075	0.000	0.08
MSS Disorganized	0.341	0.000	0.13	−141	0.000	0.02	0.037	0.171	0.00	0.096	0.000	0.01	0.020	0.464	0.00	0.159	0.000	**0.19**
CAPE Positive	0.212	0.000	0.05	−0.057	0.045	0.00	0.046	0.106	0.00	0.067	0.019	0.00	0.092	0.001	0.01	0.069	0.000	0.07
Suspiciousness	0.298	0.000	0.10	−0.174	0.000	0.03	0.032	0.239	0.00	0.076	0.005	0.01	0.045	0.100	0.00	0.137	0.000	**0.16**
Ideas of Reference	0.176	0.000	0.03	−0.039	0.173	0.00	0.046	0.107	0.00	0.069	0.016	0.01	0.043	0.130	0.00	0.046	0.000	0.05
Affective dysregulation																		
Depression	0.411	0.000	**0.21**	−0.150	0.000	0.03	0.012	0.644	0.00	0.036	0.176	0.00	0.034	0.188	0.00	0.204	0.000	**0.26**
Anxiety	0.331	0.000	0.12	−0.107	0.000	0.01	−0.027	0.332	0.00	0.020	0.463	0.00	0.059	0.031	0.00	0.129	0.000	0.**15**
Positive mental health																		
Well-being	−0.401	0.000	**0.19**	0.134	0.000	0.02	−0.016	0.546	0.00	0.000	0.999	0.00	0.053	0.046	0.01	0.187	0.000	**0.23**
Resilience	−0.260	0.000	0.07	0.144	0.000	0.02	−0.065	0.019	0.00	−0.026	0.357	0.00	0.110	0.000	0.01	0.114	0.000	0.13
Self-esteem	−0.341	0.000	0.12	0.152	0.000	0.03	0.001	0.963	0.00	−0.011	0.674	0.00	0.041	0.130	0.00	0.146	0.000	**0.17**

Factor scores were entered simultaneously to examine their unique contribution. *f*^2^ was calculated separately for each model. Bootstrap procedures (with 2,000 samples) were employed; According to Cohen (1992), *f*^2^ values of 0.02 or higher indicate small effect sizes, 0.15 indicate medium effect sizes (highlighted in bold), and ≥ 0.35 indicate large effect sizes (highlighted in bold and italic).

The total effects of the model, including the general and specific factors as predictors, were significantly associated with all primary outcome domains. Effect sizes were moderate for the P-factor and Positive Mental Health Index, whereas the effect size was large for functioning.

The general factor (moderate effect size) and Positive Experiences (small effect) were significantly associated with all outcome domains in the expected direction. Maternal Adversity showed a small but significant association with the P-factor, Paternal Adversity with functioning, and Role Reversal with the Positive Mental Health index—driven by an association with resilience; however, the last two did not reach the small effect size criterion.

At the individual scale level, the total effects of the model showed positive associations of moderate effect size with disorganized schizotypy, suspiciousness, depression, well-being, and self-esteem. These effects were primarily driven by the general factor, which also demonstrated small to moderate effect size associations for the same outcomes.

Significant inverse associations were found for Positive Experiences with negative and disorganized schizotypy, suspiciousness, and depression, as well as positive associations with well-being, resilience and self-esteem (small effect sizes). Paternal Adversity presented a small negative association with resilience, Maternal Adversity showed a small positive association with negative and disorganized schizotypy, and Role Reversal exhibited a positive correlation with positive schizotypy and resilience.

The model explained 22% of the variance in the P-factor, 17.3% in Positive Mental Health, and 29.1% in functioning. For the individual scales, it explained between 4.6 and 20.4% of the variance in psychopathology symptoms and between 11.4 and 18.7% for the positive mental health indicators. Correlations were closely comparable for the analogous regression analyses of the Early Exposome and its specific dimensions with both outcome domains and individual measures.

## 4 Discussion

To our knowledge, this is the first investigation integrating both positive and adverse early experiences within a multifactorial exposome framework and testing their impact not only on psychopathology, but also on positive mental health outcomes and functioning. The bifactor model applied revealed a general factor and four specific factors: Positive Experiences, Paternal Adversity, Maternal Adversity and Role Reversal, which presented distinct patterns of associations with psychopathology, well-being and functioning. While the general factor captured the overarching negative impact of adversity on all psychological outcomes, Positive Experiences emerged as a robust predictor of improved well-being and as a protective factor against psychopathology, highlighting the critical importance of peer and social support during formative years. Importantly, experiences of maternal adversity showed a negative impact on individuals’ psychopathology, whereas paternal adversity was slightly related to poorer functioning. Overall, these findings underscore the intricate nature of early environmental influences, illustrating that the dynamic interplay between negative and positive experiences originating from diverse figures plays a pivotal role in shaping pathways of risk and resilience during early development.

### 4.1 Modeling early experiences

Unlike dimensional adversity models, that differentiate by the type of experience (e.g., threat or deprivation; McLaughlin and Sheridan, 2016), our analyses revealed separate dimensions for paternal and maternal adversity. Therefore, it seems that grouping experiences by the source of adversity in combination with the nature may allow for a better understanding of how different relational contexts influence developmental outcomes. This finding reflects the complexity of early environmental influences and the potential co-existing roles of different figures in a child’s life (e.g., one parent may be a source of adversity, while the other may provide protection). This aligns with evidence that mothers and fathers often play distinct roles in child’s development, reflecting unique patterns of caregiving and adversity. Research suggests that within a family unit, fathers are often more involved in a child’s social development (Yaacob, 2006), whereas mothers frequently assume the primary caregiving role (Bornstein et al., 2018). This translates into more time spent with children (Cui et al., 2018), which increases the likelihood of mothers being involved in conflicts with their offspring (Bornstein, 2007) and results in a more direct exposure to maternal stress or adversity (Gryczkowski et al., 2010). The predominance of female participants in our sample (76%) might also be relevant to interpreting broader gender-based differences in parenting dynamics (although the study still included nearly 300 male participants). For example, maternal adversity has been shown to exert a stronger influence on psychopathology in female offspring, whereas paternal adversity has a greater impact on males (Oshio and Umeda, 2016). Similarly, research has found that children’s behaviors often correlate more strongly with the parenting style of the same-gender parent (Hoeve et al., 2011; Long et al., 2018). It is relevant to note that most factor-analytic research has not included measures that distinguish maternal and paternal behaviors (e.g., PBI), which may account for the lack of differentiation of these figure-related factors in other studies. Our results, concurrent with longstanding clinical observations, suggest that the differentiation of maternal and paternal caregiving (or other caregiving configurations in contemporary society) provides a more accurate understanding of early relational contexts and how these dynamics uniquely shape psychological outcomes. However, further research with more gender-balanced samples is necessary to confirm and extend these findings.

### 4.2 Associations between the early exposome and psychopathology, positive mental health and functioning

As expected, the general exposome factor—reflecting adversity—was associated with elevations in all psychopathology measures as well as diminished positive mental health and functioning. This trans-syndromic effect indicates broad, undifferentiated effects, as expected from an overarching score. The strongest association was with decreased functioning followed by lower well-being and depressive symptoms. These findings support the predictive value of scores capturing early adversities’ cumulative effects in forecasting the risks of various psychopathological outcomes (Morgan et al., 2020; Khan et al., 2022), functioning (McGinnis et al., 2022) and positive mental health indicators (Keinan et al., 2012). Additionally, this approach refines traditional cumulative scores, as modeling latent dimensions into factors has been shown to yield more accurate predictions (Brumley et al., 2019; Gizdic et al., 2023).

Positive Experiences was associated with better positive mental health and showed a large inverse association with functioning and trans-syndromic psychopathology. The nature of our sample—primarily college students with relatively low trauma exposure and possibly a higher range positive experiences—might have contributed to stronger effects for this factor compared to others. This factor comprised both peer and family experiences; however, family-related items (e.g., “My family was a source of strength and help”), lost prominence as they loaded higher onto the general score. Thus, Positive Experiences predominantly reflected variance related to peer support, especially in school contexts. These findings align with developmental research on the significance of peer relationships in late childhood and adolescence (e.g., Wang and Hu, 2021), school connectedness (Goetschius et al., 2023), and close friendships (Ku et al., 2024). Furthermore, Positive Experiences included emotional support items (e.g., “I felt loved”), highlighting the strong connection between social networks and feeling emotionally supported. This aligns with a longitudinal study by Sheinbaum et al. (2024) reporting emotional neglect to be associated with loneliness and lower social support. This may also explain the inverse association of Positive Experiences with negative psychotic symptoms, as neglect has been consistently associated with negative features over and above other adversity dimensions (Alameda et al., 2021; Bailey et al., 2018).

The Paternal and Maternal Adversity factors included inverse loadings of some positive experiences (items from the PBI care subscale) but were predominantly characterized by adversity (antipathy, psychological abuse, and overprotection). Notably, Maternal Adversity included more severe items absent in Paternal Adversity (e.g., “Said she wanted me dead,” “Deprived me of light, food or company”) and was more heavily loaded with negative items, whereas Paternal Adversity included more positive items. This may have influenced the detection of associations with psychopathology but not positive mental health. These findings align with studies showing maternal adversity, including maternal alcohol abuse (Long et al., 2018) or mental disorders (Shih et al., 2023; Pilowsky et al., 2014) has a greater impact on offspring psychopathology (including anxiety, depression, and other behavioral issues) than paternal adversity. Conversely, Paternal Adversity was associated with poorer functioning, consistent with studies suggesting fathers’ predominant role in social development, social status, and skills (Leidy et al., 2013; Feldman, 2023), and showed a slight positive association with resilience, supporting evidence that father involvement in caregiving fosters resilience in children’s development (Feldman, 2023).

Role Reversal was not associated with the main outcome domains and showed the fewest associations with individual measures, likely because, apart from role reversal items, this factor included low-endorsed items of parental violence and physical neglect, representing severe adversity. Interestingly, it showed a small positive association with resilience, which might be related to the premature adoption of adult roles—problem-solving, coping with difficulties, and resolving the dissonance of becoming the caretaker—and aligns with the potential positive outcomes of distress through resilience, as conceptualized by post-traumatic growth (Black and Wright, 2012), a term describing positive psychological adaptations after overcoming challenging situations (Kadri et al., 2025).

Effect sizes were larger for the general factor than for the specific factors, as expected in bifactor models, where the general factor absorbs much of the shared variance across items and the specific factors reflect residual, highly specific variance. As a result, their correlations with external outcomes are constrained, yielding smaller effect sizes. Alternative models, like the correlated traits model applied to exposome subdomains by Pries et al. (2022), distribute variance differently, often producing stronger associations. In this approach, item variance is fully allocated to each factor and factors can correlate, capturing both within- and between-factor variance. While this can increase effect sizes, it reduces interpretability because nonspecific variance is included in each factor (Reise, 2012). In contrast, the smaller associations for specific factors in our bifactor model are statistically significant and theoretically meaningful, revealing what each construct contributes beyond the general factor—a central aim of our study. Both approaches have trade-offs, and model choice depends on research goals (Henry et al., 2021). In our case, the bifactor model provides a clearer test of cumulative versus domain-specific influences. More broadly, these trade-offs underscore the need for further research on balancing specificity and generality in modeling complex phenomena like early experiences.

### 4.3 Challenges in the operationalization of positive and negative experiences

Despite efforts to include a substantial number of items capturing positive experiences, the higher proportion of negative items (60 negative vs. 39 positive) likely drove the direction of the general factor toward adversity (Fabrigar and Wegener, 2012), with positive items loading inversely on this factor. Consequently, the Early Exposome scores do not have a bipolar nature but rather indicate the presence and absence of adversity across a unipolar dimension. A relevant example to consider the validity of this commonly used approach is that of positive and negative affect. These two valences are typically conceptualized as separate dimensions rather than opposite ends of a single spectrum (Watson et al., 1988). Similarly, it might be argued that decreasing the score of negative environmental experiences due to the existence of positive ones is not a proper way of modeling them, as it is well-established that they can be experienced simultaneously and separately, and the effect of positive experiences probably extends beyond compensating the level of adversity experienced. In this regard, methodologies such as cluster or latent class analysis could help identify unique profiles of early experiences, revealing distinct patterns of adversity and resilience that inform our understanding of individual developmental trajectories.

### 4.4 Strengths and limitations

A key strength of this study is the assessment of a wide range of positive and negative early life experiences—as well as the trans-syndromic approach to examine their impact on psychopathology—in a large sample of young adults. Studying young adults is particularly suited for examining these associations, as this developmental stage marks a peak period for the onset of psychopathology and offers key prevention opportunities (Cicchetti, 2023). Unlike most studies focusing primarily on symptoms, we also examined functioning and positive mental health outcomes. Furthermore, bifactor modeling of early experiences provides a novel operationalization that reduces the divide between cumulative and specificity approaches by capturing fine-grained variance within specific factors and item-level associations reflecting commonalities of early experiences.

A limitation is the cross-sectional design and reliance on retrospective self-reports to assess early experiences. However, concerns about self-report reliability have been challenged by evidence showing high corroboration rates for abuse reports, even among psychiatric patients (74–82%; Read et al., 2005) and research suggests interview-based methods are not inherently more valid or reliable than self-reports (Linscott and van Os, 2013). While the social environment cannot be reduced to single aspects (Gudi-Mindermann et al., 2023), we focused on social interactions as they connect the individual to the external world and play a fundamental role in health and development. In line with Colomina et al. (2018), we emphasize psychosocial environmental exposures, particularly early life experiences that shape neural circuits and influence susceptibility to environmental factors. However, future research should expand to other social dimensions, such as socioeconomic circumstances and sociodemographic characteristics.

The sample, composed of young adults with mostly middle-to-high socioeconomic status (64.7%) from two universities in Barcelona and an overrepresentation of women—a common challenge in voluntary participation as women are generally more likely to volunteer (Lobato et al., 2014)—may limit generalizability. Nevertheless, the study included a substantial number of male participants. Our findings provide insights into source-specific environmental factors; however, given the exploratory nature and sample composition, replication in more diverse populations is needed to further confirm the factorial structure and associations, as the restricted sample may attenuate effects due to limited range.

## 5 Conclusion

Our findings underscore the need to develop and incorporate measures that capture the full spectrum of environmental experiences, adverse and positive. Acknowledging the coexistence and dynamic interplay of supportive and adverse experiences will help to better understand the pathways of risk and resilience to mental health and will probably partially account for the heterogeneity of outcomes related to adversity exposures (McLaughlin and Gabard-Durnam, 2022). Results also highlight the importance of assessing different relational contexts (including relationships with peers and parental figures) in the study of environmental factors. Overall, accounting for the multifaceted realities of early development is crucial to inform preventive interventions and clinical practices aimed at promoting resilience and well-being in individuals who have faced childhood adversity.

## Data Availability

The raw data supporting the conclusions of this article will be made available by the authors, without undue reservation.

## References

[ref1] Alameda L. Christy A. Rodriguez V. Salazar De Pablo G. Thrush M. Shen Y.. (2021). Association between specific childhood adversities and symptom dimensions in people with psychosis: systematic review and meta-analysis. Schizophr. Bull. 47, 975–985. doi: 10.1093/schbul/sbaa199, PMID: 33836526 PMC8266673

[ref3] Bailey T. Alvarez-Jimenez M. Garcia-Sanchez A. M. Hulbert C. Barlow E. Bendall S. (2018). Childhood trauma is associated with severity of hallucinations and delusions in psychotic disorders: a systematic review and meta-analysis. Schizophr. Bull. 44, 1111–1122. doi: 10.1093/schbul/sbx161, PMID: 29301025 PMC6101549

[ref4] Ballard E. D. Van Eck K. Musci R. J. Hart S. R. Storr C. L. Breslau N.. (2015). Latent classes of childhood trauma exposure predict the development of behavioral health outcomes in adolescence and young adulthood. Psychol. Med. 45, 3305–3316. doi: 10.1017/S0033291715001300, PMID: 26149665

[ref5] Bandoli G. Campbell-Sills L. Kessler R. C. Heeringa S. G. Nock M. K. Rosellini A. J.. (2017). Childhood adversity, adult stress, and the risk of major depression or generalized anxiety disorder in US soldiers: a test of the stress sensitization hypothesis. Psychol. Med. 47, 2379–2392. doi: 10.1017/S0033291717001064, PMID: 28443533 PMC5595661

[ref6] Barzilay R. Pries L. K. Moore T. M. Gur R. E. van Os J. Rutten B. P. F.. (2022). Exposome and trans-syndromal developmental trajectories toward psychosis. *Biol. Psychiatry Glob.* Open Sci. 2, 197–205. doi: 10.1016/j.bpsgos.2022.05.001, PMID: 36325037 PMC9616341

[ref7] Beck A. T. Epstein N. Brown G. Steer R. A. (1988). An inventory for measuring clinical anxiety: psychometric properties. J. Consult. Clin. Psychol. 56, 893–897. doi: 10.1037/0022-006X.56.6.893, PMID: 3204199

[ref8] Beck A. T. Ward C. H. Mendelson M. Mock J. Erbaugh J. (1979). Beck depression inventory. New York, United States: Psychological Corporation.

[ref9] Bernstein D. P. Stein J. A. Newcomb M. D. Walker E. Pogge D. Ahluvalia T.. (2003). Development and validation of a brief screening version of the childhood trauma questionnaire. Child Abuse Negl. 27, 169–190. doi: 10.1016/s0145-2134(02)00541-0 12615092

[ref10] Bethell C. Jones J. Gombojav N. Linkenbach J. Sege R. (2019). Positive childhood experiences and adult mental and relational health in a statewide sample: associations across adverse childhood experiences levels. JAMA Pediatr. 173:e193007. doi: 10.1001/jamapediatrics.2019.3007, PMID: 31498386 PMC6735495

[ref11] Black B. P. Wright P. (2012). Posttraumatic growth and transformation as outcomes of perinatal loss. Illness Crisis Loss 20, 225–237. doi: 10.2190/IL.20.3.b

[ref12] Bornstein M. H. (2007). “Parenting science and practice” in Handbook of child psychology. eds. Damon W. Lerner R. M. Renninger K. A. Siegel I. E. (Hoboken, NJ: Wiley).

[ref13] Bornstein M. H. Putnick D. L. Suwalsky J. T. D. (2018). Parenting cognitions → parenting practices → child adjustment? The standard model. Dev. Psychopathol. 30, 399–416. doi: 10.1017/S0954579417000931, PMID: 28625208 PMC5823787

[ref14] Brumley L. D. Brumley B. P. Jaffee S. R. (2019). Comparing cumulative index and factor analytic approaches to measuring maltreatment in the national longitudinal study of adolescent to adult health. Child Abus. Negl. 87, 65–76. doi: 10.1016/j.chiabu.2018.08.014 30146090

[ref15] Campbell-Sills L. Stein M. B. (2007). Psychometric analysis and refinement of the Connor-Davidson resilience scale (CD-RISC): validation of a 10-item measure of resilience. J. Trauma. Stress. 20, 1019–1028. doi: 10.1002/jts.20271, PMID: 18157881

[ref16] Carver C. S. Johnson S. L. Timpano K. R. (2017). Toward a functional view of the P factor in psychopathology. Clin. Psychol. Sci. 5, 880–889. doi: 10.1177/2167702617710037, PMID: 29057170 PMC5646702

[ref17] Caspi A. Houts R. M. Belsky D. W. Goldman-Mellor S. J. Harrington H. Israel. (2014). The p factor: one general psychopathology factor in the structure of psychiatric disorders? Clin. Psychol. Sci. 2, 119–137. doi: 10.1177/2167702613497473 25360393 PMC4209412

[ref18] Catalan A. Angosto V. Díaz A. Valverde C. de Artaza M. G. Sesma E.. (2017). Relation between psychotic symptoms, parental care and childhood trauma in severe mental disorders. Psychiatry Res. 251, 78–84. doi: 10.1016/j.psychres.2017.02.017, PMID: 28189941

[ref19] Cecil C. A. Viding E. Fearon P. Glaser D. McCrory E. J. (2017). Disentangling the mental health impact of childhood abuse and neglect. Child Abuse Negl. 63, 106–119. doi: 10.1016/j.chiabu.2016.11.024, PMID: 27914236

[ref20] Chapman L. J. Chapman J. P. (1983). Infrequency scale for personality measures. UIUC: T. R. Kwapil.

[ref21] Cicchetti D. (2023). “A multiple levels of analysis developmental psychopathology perspective on adolescence and young adulthood” in APA handbook of adolescent and young adult development. eds. Crockett L. J. Carlo G. Schulenberg J. E. (Washington D.C., WA: American Psychological Association).

[ref22] Cohen J. (1992). A power primer. Psychol. Bull. 112, 155–159. doi: 10.1037/0033-2909.112.1.155, PMID: 19565683

[ref23] Colomina M. T. Sánchez-Santed F. Conejo N. M. Collado P. Salvador A. Gallo. (2018). The psychoexposome: a holistic perspective beyond health and disease. Psicothema 30, 5–7. doi: 10.7334/psicothema2017.244 29363463

[ref24] Crush E. Arseneault L. Moffitt T. E. Danese A. Caspi A. Jaffee S. R.. (2018). Protective factors for psychotic experiences amongst adolescents exposed to multiple forms of victimization. J. Psychiatr. Res. 104, 32–38. doi: 10.1016/j.jpsychires.2018.06.011, PMID: 29929082 PMC6109202

[ref25] Cui N. Deatrick J. A. Liu J. (2018). Maternal and paternal physical abuse: unique and joint associations with child behavioral problems. Child Abuse Negl. 76, 524–532. doi: 10.1016/j.chiabu.2017.05.003, PMID: 28532982 PMC6298424

[ref26] Dominguez A. Rivas I. Koch S. Binter A. C. Iñíguez C. Estarlich M.. (2023). Windows of susceptibility of prenatal and childhood exposure to air pollution and lung function at 6–8 years in the Spanish INMA (Infancia y Medio Ambiente) birth cohort. Env Res. 285:122585. doi: 10.1016/j.envres.2025.122585, PMID: 40816673

[ref27] Erzin G. Guloksuz S. (2021). The exposome paradigm to understand the environmental origins of mental disorders. Alpha Psychiatry 22, 171–176. doi: 10.5152/alphapsychiatry.2021.21307, PMID: 36424935 PMC9590645

[ref29] Evans G. W. Li D. Whipple S. S. (2013). Cumulative risk and child development. Psychol. Bull. 139, 1342–1396. doi: 10.1037/a0031808, PMID: 23566018

[ref30] Fabrigar L. R. Wegener D. T. (2012). Exploratory factor analysis. Oxford, United Kingdom: Oxford University Press.

[ref31] Feldman R. (2023). Father contribution to human resilience. Dev. Psychopathol. 35, 2402–2419. doi: 10.1017/S0954579423000354, PMID: 37039132

[ref32] Gizdic A. Sheinbaum T. Kwapil T. R. Barrantes-Vidal N. (2023). Empirically-derived dimensions of childhood adversity and cumulative risk: associations with measures of depression, anxiety, and psychosis-spectrum psychopathology. Eur. J. Psychotraumatol. 14:2222614. doi: 10.1080/20008066.2023.2222614, PMID: 37377079 PMC10308870

[ref33] Goetschius L. G. McLoyd V. C. Hein T. C. Mitchell C. Hyde L. W. Monk C. S. (2023). School connectedness as a protective factor against childhood exposure to violence and social deprivation: a longitudinal study of adaptive and maladaptive outcomes. Dev. Psychopathol. 35, 1219–1234. doi: 10.1017/S0954579421001140, PMID: 34779377 PMC10037103

[ref34] Gross G. M. Kwapil T. R. Raulin M. L. Silvia P. J. Barrantes-Vidal N. (2018). The multidimensional schizotypy scale-brief: scale development and psychometric properties. Psychiatry Res. 261, 7–13. doi: 10.1016/j.psychres.2017.12.033, PMID: 29272752

[ref35] Gryczkowski M. R. Jordan S. S. Mercer S. H. (2010). Differential relations between mothers' and fathers' parenting practices and child externalizing behavior. J. Child Fam. Stud. 19, 539–546. doi: 10.1007/s10826-009-9326-2 28952046

[ref36] Gudi-Mindermann H. White M. Roczen J. Riedel N. Dreger S. Bolte G. (2023). Integrating the social environment with an equity perspective into the exposome paradigm: a new conceptual framework of the social Exposome. Environ. Res. 233:116485. doi: 10.1016/j.envres.2023.116485, PMID: 37352954

[ref37] Guloksuz S. van Os J. Rutten B. P. F. (2018). The Exposome paradigm and the complexities of environmental research in psychiatry. JAMA Psychiatry 75, 985–986. doi: 10.1001/jamapsychiatry.2018.1211, PMID: 29874362

[ref38] Heinonen E. Knekt P. Härkänen T. Virtala E. Lindfors O. (2018). Associations of early childhood adversities with mental disorders, psychological functioning, and suitability for psychotherapy in adulthood. Psychiatry Res. 264, 366–373. doi: 10.1016/j.psychres.2018.04.011, PMID: 29677619

[ref39] Henry L. M. Gracey K. Shaffer A. Ebert J. Kuhn T. Watson K. H.. (2021). Comparison of three models of adverse childhood experiences: associations with child and adolescent internalizing and externalizing symptoms. J. Abnorm. Psychol. 130, 9–25. doi: 10.1037/abn0000644, PMID: 33271039 PMC8687696

[ref40] Hoeve M. Dubas J. S. Gerris J. R. van der Laan P. H. Smeenk W. (2011). Maternal and paternal parenting styles: unique and combined links to adolescent and early adult delinquency. J. Adolesc. 34, 813–827. doi: 10.1016/j.adolescence.2011.02.004, PMID: 21397317

[ref41] Hostinar C. E. Swartz J. R. Alen N. V. Guyer A. E. Hastings P. D. (2023). The role of stress phenotypes in understanding childhood adversity as a transdiagnostic risk factor for psychopathology. J. Psychopathol. Clin. Sci. 132, 277–286. doi: 10.1037/abn0000619, PMID: 37126060 PMC10153067

[ref42] Huang C. X. Halfon N. Sastry N. Chung P. J. Schickedanz A. (2023). Positive childhood experiences and adult health outcomes. Pediatrics 152:e2022060951. doi: 10.1542/peds.2022-060951, PMID: 37337829 PMC10312234

[ref43] Humphreys K. L. LeMoult J. Wear J. G. Piersiak H. A. Lee A. Gotlib I. H. (2020). Child maltreatment and depression: a meta-analysis of studies using the childhood trauma questionnaire. Child Abuse Negl. 102:104361. doi: 10.1016/j.chiabu.2020.104361, PMID: 32062423 PMC7081433

[ref001] ISGlobal. (2020). HELIX — Novel tools for integrating early-life environmental exposures and child health across Europe. Barcelona: ISGlobal. Available at: https://www.isglobal.org/en/-/helix-the-human-early-life-exposome-novel-tools-for-integrating-early-life-environmental-exposures-and-child-health-across-europe

[ref002] ISGlobal. (2021). CityExposomeCat — An exposome approach to urban health: Individualized environmental exposure assessment in an adults population cohort study (GCAT). Barcelona: ISGlobal. Available at: https://www.isglobal.org/en/-/cityexposomecat-exposome-approach-to-urban-health

[ref44] Kadri A. Gracey F. Leddy A. (2025). What factors are associated with posttraumatic growth in older adults? A systematic review. Clin. Gerontol. 48, 4–21. doi: 10.1080/07317115.2022.2034200, PMID: 35138231

[ref45] Keinan G. Shrira A. Shmotkin D. (2012). The association between cumulative adversity and mental health: considering dose and primary focus of adversity. Qual. Life Res. 21, 1149–1158. doi: 10.1007/s11136-011-0035-0, PMID: 21983715

[ref46] Khan M. K. Khan G. Mufti A. A. (2022). Association of childhood trauma exposure with adult psychiatric disorders and functional outcomes. Glob. Drug Des. Dev. Rev. 7, 26–32. doi: 10.31703/gdddr.2022(VII-I).04 PMC6324370 30646356

[ref47] Khomenko S. Burov A. Dzhambov A. M. de Hoogh K. Helbich M. Mijling B.. (2023). Health burden and inequities of urban environmental stressors in Sofia, Bulgaria. Environ. Res. 279:121782. doi: 10.1016/j.envres.2025.121782 40345423

[ref48] Kolb B. Mychasiuk R. Muhammad A. Gibb R. (2013). Brain plasticity in the developing brain. Prog. Brain Res. 207, 35–64. doi: 10.1016/B978-0-444-63327-9.00005-9, PMID: 24309250

[ref49] Ku B. S. Ren J. Compton M. T. Druss B. G. Guo S. Walker E. F. (2024). The association between neighborhood-level social fragmentation and distressing psychotic-like experiences in early adolescence: the moderating role of close friends. Psychol. Med. 54, 2172–2180. doi: 10.1017/S0033291724000278, PMID: 38362835 PMC11327384

[ref50] Lacey R. E. Minnis H. (2020). Practitioner review: twenty years of research with adverse childhood experience scores – advantages, disadvantages and applications to practice. J. Child Psychol. Psychiatry 61, 116–130. doi: 10.1111/jcpp.13135, PMID: 31609471

[ref51] Leidy M. S. Schofield T. J. Parke R. D. (2013). “Fathers' contributions to children's social development” in Handbook of father involvement: Multidisciplinary perspectives. eds. Cabrera N. J. Tamis-LeMonda C. S. (New York, NY: Routledge/Taylor and Francis Group).

[ref52] Li M. Cassis T. D'Arcy C. Low N. Meng X. (2020). Development and validation of a brief form of the childhood adversities questionnaire among a population of mood disorders. J. Interpers. Violence 373, 1903–1928. doi: 10.1177/0886260520933037 32618218

[ref53] Linscott R. J. van Os J. (2013). An updated and conservative systematic review and meta-analysis of epidemiological evidence on psychotic experiences in children and adults: on the pathway from proneness to persistence to dimensional expression across mental disorders. Psychol. Med. 43, 1133–1149. doi: 10.1017/S0033291712001626, PMID: 22850401

[ref54] Lobato L. Bethony J. M. Pereira F. B. Grahek S. L. Diemert D. Gazzinelli M. F. (2014). Impact of gender on the decision to participate in a clinical trial: a cross-sectional study. BMC Public Health 14:156. doi: 10.1186/1471-2458-14-1156 25377601 PMC4232621

[ref55] Long E. C. Lönn S. L. Sundquist J. Sundquist K. Kendler K. S. (2018). The role of parent and offspring sex on risk for externalizing psychopathology in offspring with parental alcohol use disorder: a national Swedish study. Soc. *Psychiatry Psychiatr. Epidemiol.* 53, 1381–1389. doi: 10.1007/s00127-018-1563-5, PMID: 30019183 PMC6252126

[ref56] Masten A. S. (2006). Developmental psychopathology: pathways to the future. Int. J. Behav. Dev. 30, 47–54. doi: 10.1177/0165025406059974

[ref57] McGinnis E. W. Sheridan M. Copeland W. E. (2022). Impact of dimensions of early adversity on adult health and functioning: a 2-decade, longitudinal study. Dev. Psychopathol. 34, 527–538. doi: 10.1017/S095457942100167X, PMID: 35074038 PMC9309184

[ref58] McGrath J. J. Lim C. C. W. Plana-Ripoll O. Holtz Y. Agerbo E. Momen N. C.. (2020). Comorbidity within mental disorders: a comprehensive analysis based on 145 990 survey respondents from 27 countries. *Epidemiol. Psychiatr.* Sci. 29:e153. doi: 10.1017/S2045796020000633, PMID: 32782057 PMC7443806

[ref59] McLaughlin K. A. Colich N. L. Rodman A. M. Weissman D. G. (2020). Mechanisms linking childhood trauma exposure and psychopathology: a transdiagnostic model of risk and resilience. BMC Med. 18, 1–11. doi: 10.1186/s12916-020-01561-6 32238167 PMC7110745

[ref60] McLaughlin K. A. Gabard-Durnam L. (2022). Experience-driven plasticity and the emergence of psychopathology: a mechanistic framework integrating development and the environment into the research domain criteria (RDoC) model. J. Psychopathol. Clin. Sci. 131, 575–587. doi: 10.1037/abn0000598, PMID: 35901389 PMC9346621

[ref61] McLaughlin K. A. Sheridan A. M. (2016). Beyond cumulative risk: a dimensional approach to childhood adversity. Curr. Dir. Psychol. Sci. 25, 239–245. doi: 10.1177/0963721416655883 27773969 PMC5070918

[ref62] McLaughlin K. A. Sheridan M. A. Humphreys K. L. Belsky J. Ellis B. J. (2021). The value of dimensional models of early experience: thinking clearly about concepts and categories. Perspect. Psychol. Sci. 16, 1463–1472. doi: 10.1177/1745691621992346, PMID: 34491864 PMC8563369

[ref63] Miller A. B. Sheridan M. A. Hanson J. L. McLaughlin K. A. Bates J. E. Lansford J. E.. (2018). Dimensions of deprivation and threat, psychopathology, and potential mediators: a multi-year longitudinal analysis. J. Abnorm. Psychol. 127, 160–170. doi: 10.1037/abn0000331, PMID: 29528670 PMC5851283

[ref64] Moore T. M. Reise S. P. Depaoli S. Haviland M. G. (2015). Iteration of partially specified target matrices: applications in exploratory and Bayesian confirmatory factor analysis. Multivar. Behav. Res. 50, 149–161. doi: 10.1080/00273171.2014.973990, PMID: 26609875 PMC4665092

[ref65] Moore T. M. Visoki E. Argabright S. T. Didomenico G. E. Sotelo I. Wortzel J. D.. (2022). Modeling environment through a general exposome factor in two independent adolescent cohorts. Exposome. 2:osac010. doi: 10.1093/exposome/osac010, PMID: 36606125 PMC9798749

[ref66] Morgan C. Gayer-Anderson C. Beards S. Hubbard K. Mondelli V. Di Forti M.. (2020). Threat, hostility and violence in childhood and later psychotic disorder: population-based case-control study. Br. J. Psychiatry 217, 575–582. doi: 10.1192/bjp.2020.133 32778182 PMC7525109

[ref67] Narayan A. J. Rivera L. M. Bernstein R. E. Harris W. W. Lieberman A. F. (2018). Positive childhood experiences predict less psychopathology and stress in pregnant women with childhood adversity: a pilot study of the benevolent childhood experiences (BCEs) scale. Child Abuse Negl. 78, 19–30. doi: 10.1016/j.chiabu.2017.09.022, PMID: 28992958

[ref68] Oshio T. Umeda M. (2016). Gender-specific linkages of parents’ childhood physical abuse and neglect with children’s problem behaviour: evidence from Japan. BMC Public Health 16:403. doi: 10.1186/s12889-016-3072-3, PMID: 27179941 PMC4867086

[ref69] Parker G. Tupling H. Brown L. (1979). A parental bonding instrument. Br. J. Med. Psychol. 52, 1–10. doi: 10.1037/t06510-000

[ref70] Pfaltz M. C. Halligan S. L. Haim-Nachum S. Sopp M. R. Åhs F. Bachem R.. (2022). Social functioning in individuals affected by childhood maltreatment: establishing a research agenda to inform interventions. Psychother. Psychosom. 91, 238–251. doi: 10.1159/000523667 35381589 PMC9393832

[ref71] Pietrek C. Elbert T. Weierstall R. Müller O. Rockstroh B. (2013). Childhood adversities in relation to psychiatric disorders. Psychiatry Res. 206, 103–110. doi: 10.1016/j.psychres.2012.11.003, PMID: 23261184

[ref72] Pilowsky D. J. Wickramaratne P. Poh E. Hernandez M. Batten L. A. Flament M. F.. (2014). Psychopathology and functioning among children of treated depressed fathers and mothers. J. Affect. Disord. 164, 107–111. doi: 10.1016/j.jad.2014.04.012, PMID: 24856562 PMC4152938

[ref73] Pries L. K. Moore T. M. Visoki E. Sotelo I. Barzilay R. Guloksuz S. (2022). Estimating the association between exposome and psychosis as well as general psychopathology: results from the ABCD study. Biol. Psychiatry Glob. Open Sci. 2, 283–291. doi: 10.1016/j.bpsgos.2022.05.005, PMID: 36325038 PMC9616253

[ref74] Raine A. (1991). The SPQ: a scale for the assessment of schizotypal personality based on DSM-III-R criteria. Schizophr. Bull. 17, 555–564. doi: 10.1093/schbul/17.4.555, PMID: 1805349

[ref75] Read J. van Os J. Morrison A. P. Ross C. A. (2005). Childhood trauma, psychosis and schizophrenia: a literature review with theoretical and clinical implications. Acta Psychiatr. Scand. 112, 330–350. doi: 10.1111/j.1600-0447.2005.00634.x, PMID: 16223421

[ref76] Redican E. Rawers C. McElroy E. Hyland P. Karatzias T. Ben-Ezra M.. (2023). Development and initial validation of a short form of the memories of home and family scale. Advers. Resil. Sci. 4, 235–244. doi: 10.1007/s42844-023-00097-x, PMID: 37361561 PMC10148702

[ref77] Reise S. P. (2012). The rediscovery of bifactor measurement models. Multivar. Behav. Res. 47, 667–696. doi: 10.1080/00273171.2012.715555, PMID: 24049214 PMC3773879

[ref78] Rosenberg M. (1965). Society and the adolescent self-image. New Jersey, United States: Princeton University Press.

[ref79] Samson J. A. Newkirk T. R. Teicher M. H. (2024). Practitioner review: neurobiological consequences of childhood maltreatment – clinical and therapeutic implications for practitioners. J. Child Psychol. Psychiatry 65, 369–380. doi: 10.1111/jcpp.13883, PMID: 37609790

[ref80] Schäfer J. L. McLaughlin K. A. Manfro G. G. Pan P. Rohde L. A. Miguel E. C.. (2023). Threat and deprivation are associated with distinct aspects of cognition, emotional processing, and psychopathology in children and adolescents. Dev. Sci. 26:e13267. doi: 10.1111/desc.13267, PMID: 35417607 PMC10028496

[ref81] Sheinbaum T. Gizdic A. Kwapil T. R. Barrantes-Vidal N. (2024). A longitudinal study of the impact of childhood adversity dimensions on social and psychological factors and symptoms of psychosis, depression, and anxiety. Schizophr. Res. 270, 102–110. doi: 10.1016/j.schres.2024.05.016, PMID: 38889654

[ref82] Shih E. W. Ahmad S. I. Bush N. R. Roubinov D. Tylavsky F. Graff C.. (2023). A path model examination: maternal anxiety and parenting mediate the association between maternal adverse childhood experiences and children's internalizing behaviors. Psychol. Med. 53, 112–122. doi: 10.1017/S0033291721001203, PMID: 34001294 PMC9290334

[ref83] Shonkoff J. P. Phillips D. A. (2000). From neurons to neighborhoods: The science of early childhood development. Washington, United States: National Academies Press. 25077268

[ref84] Sideli L. Murray R. M. Schimmenti A. Corso M. La Barbera D. Trotta A.. (2020). Childhood adversity and psychosis: a systematic review of bio-psycho-social mediators and moderators. Psychol. Med. 50, 1761–1782. doi: 10.1017/S0033291720002172, PMID: 32624020

[ref85] Smith G. T. Atkinson E. A. Davis H. A. Riley E. N. Oltmanns J. R. (2020). The general factor of psychopathology. Annu. Rev. Clin. Psychol. 16, 75–98. doi: 10.1146/annurev-clinpsy-071119-115848, PMID: 32040926

[ref86] Smith K. E. Pollak S. D. (2021). Rethinking concepts and categories for understanding the neurodevelopmental effects of childhood adversity. Perspect. Psychol. Sci. 16, 67–93. doi: 10.1177/1745691620920725, PMID: 32668190 PMC7809338

[ref87] Snyder H. R. Hankin B. L. (2017). All models are wrong, but the p factor model is useful: reply to Widiger and Oltmanns (2017 and Bonifay, lane, and Reise 2017). Clin. Psychol. Sci. 5, 187–189. doi: 10.1177/2167702616659389, PMID: 28286706 PMC5343281

[ref88] Stefanis N. C. Hanssen M. Smirnis N. K. Avramopoulos D. A. Evdokimidis I. K. Stefanis. (2002). Evidence that three dimensions of psychosis have a distribution in the general population. Psychol. Med. 32, 347–358. doi: 10.1017/S0033291701005141 11866327

[ref89] Teicher M. H. Samson J. A. Anderson C. M. Ohashi K. (2016). The effects of childhood maltreatment on brain structure, function and connectivity. Nat. Rev. Neurosci. 17, 652–666. doi: 10.1038/nrn.2016.111, PMID: 27640984

[ref90] Tennant R. Hiller L. Fishwick R. Platt S. Joseph S. Weich S.. (2007). The Warwick-Edinburgh mental well-being scale (WEMWBS): development and UK validation. Health Qual. Life Outcomes 5, 1–13. doi: 10.1186/1477-7525-5-63, PMID: 18042300 PMC2222612

[ref91] Thakkar K. N. McCleery A. Minor K. S. Lee J. Humpston C. S. Chopik W. J.. (2023). Moving from risk to resilience in psychosis research. Nat. Rev. Psychol. 2, 537–555. doi: 10.1038/s44159-023-00205-9

[ref92] Tyrer P. Nur U. Crawford M. Karlsen S. MacLean C. Rao B.. (2005). The social functioning questionnaire: a rapid and robust measure of perceived functioning. Int. J. Soc. Psychiatry 51, 265–275. doi: 10.1177/0020764005057391, PMID: 28095167

[ref93] Wang G. Hu W. (2021). Peer relationships and college students’ cooperative tendencies: roles of interpersonal trust and social value orientation. Front. Psychol. 12:656412. doi: 10.3389/fpsyg.2021.656412, PMID: 34305721 PMC8301073

[ref94] Warkentin S. Márquez S. Vespalcová H. Knox B. Gascon M. Güil-Oumrait N.. (2022). Dietary patterns and exposure to non-persistent endocrine-disrupting chemicals during pregnancy. Environ. Int. 202:109612. doi: 10.1016/j.envint.2025.109612 PMC12364790 40578114

[ref95] Watson D. Clark L. Tellegen A. (1988). Development and validation of brief measures of positive and negative affect: the PANAS scales. J. Pers. Soc. Psychol. 54, 1063–1070. doi: 10.1037/0022-3514.54.6.1063, PMID: 3397865

[ref96] Watts A. L. Greene A. L. Bonifay W. Fried E. I. (2024). A critical evaluation of the p-factor literature. Nat. Rev. Psychol. 3, 108–122. doi: 10.1038/s44159-023-00260-2

[ref97] Wild C. P. (2005). Complementing the genome with an “exposome”: the outstanding challenge of environmental exposure measurement in molecular epidemiology. Cancer Epidemiol. Biomarkers Prev. 14, 1847–1850. doi: 10.1158/1055-9965.EPI-05-0456, PMID: 16103423

[ref98] Yaacob M. J. B. (2006). Parent-adolescent relationships and its association to adolescents’ self-esteem. Malaysian J. Med. Sci. 13, 21–24. Available at: https://pmc.ncbi.nlm.nih.gov/articles/PMC3347898/PMC3347898 22589586

