# Supplementary material for: Integration of positive environmental factors and differentiation of parental figures in social exposome research
Source: Front Psychol. 2025 Oct 27;16:1655172. doi: 10.3389/fpsyg.2025.1655172 (PMC12597736; doi:10.3389/fpsyg.2025.1655172)
Supplement: Supplementary file 1 [file Data_Sheet_1.docx]

**SUPPLEMENTARY MATERIALS**

Index of Contents

[**Table S1.** Environmental items included in initial analysis 2](#_Toc201850616)

[**Table S2**. Descriptive statistics for study variables 5](#_Toc201850617)

[**Table S3.** Pearson correlations for study variables 6](#_Toc201850618)

[**Table S4.** Factor loadings of the first EFA for general Early Exposome items 6](#_Toc201850619)

[**Table S5.** Factor loadings of the second EFA for general Early Exposome items 10](#_Toc201850620)

[**Table S6.** Loadings of third EFA for Early Exposome items differentiating parental figures 11](#_Toc201850621)

[**Table S7.** Factor loadings of the fourth EFA for all remaining Early Exposome items 13](#_Toc201850622)

[**Fig S1**. Scree plot of the final early environmental items 15](#_Toc201850623)

[**Table S8.** Factor loadings of the confirmatory factor analysis of Early Exposome items 16](#_Toc201850624)

[**Table S9.** Bifactor indices for the Early Exposome model 18](#_Toc201850625)

[**Table S10.** Factor loadings of the EFA of P-factor items 18](#_Toc201850626)

[**Table S11**. Factor loadings of the confirmatory factor analysis of P-factor items 21](#_Toc201850627)

[**Table S12.** Bifactor indices for the P-factor model 24](#_Toc201850628)

[**Table S13.** Factor loadings of the exploratory factor analysis of the Positive Mental Health items 24](#_Toc201850629)

[**Table S14.** Factor loadings of the confirmatory factor analysis of the Positive Mental Health items 25](#_Toc201850630)

[**Table S15.** Bifactor indices for the Positive Mental Health model 26](#_Toc201850631)

[**Table S16.** Correlations Early Exposome with the main outcome domains 26](#_Toc201850632)

[**Table S17.** Correlations Early Exposome with individual measures 27](#_Toc201850633)

[**Supplementary Methods** 28](#_Toc201850634)

[Iterative exploratory factor analyses for environmental data reduction 28](#_Toc201850635)

[Bifactor modeling of the early exposome 28](#_Toc201850636)

[Environmental measures 29](#_Toc201850637)

[Phenotypic measures 30](#_Toc201850638)

[*Psychopathology measures* 30](#_Toc201850639)

[Positive mental health measures 31](#_Toc201850640)

[Functioning 31](#_Toc201850641)

[**Supplementary References** 31](#_Toc201850642)

# **Table S1.** Environmental items included in initial analysis.

| **Item** | | | **Content** |  |
| --- | --- | --- | --- | --- |
| **Emotional Abuse** | | | |  |
| CTQ_3 | Family called me things like “stupid”, “lazy” or “ugly” | | | |
| CTQ_8 | Thought parents wished I had never been born | | | |
| CTQ_14 | Family said hurtful or insulting things | | | |
| CTQ_19 | Felt that someone in my family hated me | | | |
| CTQ_26 | Was emotionally abused | | | |
| **Emotional Neglect** | | | | |
| CTQ_5 | Family member made me feel valued | | | |
| CTQ_7 | Felt loved | | | |
| CTQ_13 | People in family looked out for each other | | | |
| CTQ_20 | Family felt close | | | |
| CTQ_29 | Family was source of strength | | | |
| **Physical Neglect** | | | | |
| CTQ_1 | Not enough food to eat | | | |
| CTQ_2 | Family member took care of me | | | |
| CTQ_4 | Parents too drunk or too high | | | |
| CTQ_6 | Wore dirty clothes | | | |
| CTQ_27 | Someone take to doctor if I needed it | | | |
| **Physical Abuse** | | | | |
| CTQ_9 | Got hit by family member so hard that I had to go to the doctor | | | |
| CTQ_11 | Family member hit so hard bruises or marks | | | |
| CTQ_12 | Punished with belt, board, cord or hard object | | | |
| CTQ_16 | Think physically abused | | | |
| CTQ_18 | Someone hit so hard someone noticed (teacher, doctor, neighbor) | | | |
| **Sexual Abuse** | | | | |
| CTQ_21 | Someone tried to touch/make touch them in a sexual way | | | |
| CTQ_22 | Someone threatened to hurt/tell lies about me unless I did something sexual with them | | | |
| CTQ_24 | Someone tried to make me do/watch sexual things | | | |
| CTQ_25 | Someone molested me | | | |
| CTQ_28 | I believe I was sexually abused | | | |
| **Role Reversal** | | | | |
| RR_1 | Lot of responsibility at home | | | |
| RR_2 | Expected to do a lot of housework | | | |
| RR_3 | Had to look after younger siblings | | | |
| RR_4 | Missed out seeing friends because of home responsibilities | | | |
| RR_5 | Parent/s look to you for help as a child | | | |
| RR_6 | Parent/s confided their problems in you | | | |
| RR_8 | Parent/s rely for emotional support as a child | | | |
| RR_9 | Felt concerned and worried about parents | | | |
| **Paternal Antipathy** | | | | |
| ANTIPATHY_F1 | Was difficult to please | | | |
| ANTIPATHY_F2 | Was critical of me | | | |
| ANTIPATHY_F3 | Made me feel I was a nuisance | | | |
| ANTIPATHY_F4 | Picked on me unfairly | | | |
| ANTIPATHY_F5 | Was there if I needed | | | |
| ANTIPATHY_F6 | Did not like me as much as my siblings | | | |
| **Maternal Antipathy** | | | | |
| ANTIPATHY_M1 | Was difficult to please | | | |
| ANTIPATHY_M2 | Made me feel unwanted | | | |
| ANTIPATHY_M3 | Was critical | | | |
| ANTIPATHY_M4 | Made me feel I was a nuisance | | | |
| ANTIPATHY_M5 | Picked on me unfairly | | | |
| ANTIPATHY_M6 | Did not like me as much as my siblings | | | |
| **Paternal Psychological Abuse** | | | | |
| PA_P1 | Undermined my confidence | | | |
| PA_P2 | Played on my fears | | | |
| PA_P3 | Liked to see me suffer | | | |
| PA_P4 | Humiliated me, put me down. | | | |
| PA_P5 | Shamed me in front of others | | | |
| PA_P6 | Was rejecting | | | |
| PA_P7 | Took away things I cherished | | | |
| PA_P8 | Made me feel guilty, so I would do what I was told | | | |
| **Maternal Psychological Abuse** | | | | |
| PA_M1 | Undermined my confidence | | | |
| PA_M2 | Confuse me by telling contradictory things | | | |
| PA_M3 | Played on my fears | | | |
| PA_M4 | Humiliated me, put me down | | | |
| PA_M5 | Shamed me in front of others | | | |
| PA_M6 | Was rejecting | | | |
| PA_M7 | Deprivation of light, food, or company | | | |
| PA_M8 | Made me feel guilty, so I would do what I was told | | | |
| PA_M9 | Threatened to hurt loved ones to get what she wanted | | | |
| PA_M10 | Forced me to steal or break the law | | | |
| PA_M11 | Said wanted me dead | | | |
| **Discord** | | | | |
| Discord_1 | Periods relatives (not parents) stopped talking to each other/argued a lot | | | |
| Discord_2 | Periods parents stopped talking to each other/argued a lot | | | |
| Discord_3 | Parents raised their voices | | | |
| Discord_4 | Parents intense arguments throwing things | | | |
| Discord_5 | Siblings and self often present/involved in parent's arguments | | | |
| **Violence** | | | | |
| Violence_1 | | Violence between parent throwing things/physical contact | | |
| Violence_2 | | Violence with consequences hospital, police, social services | | |
| **Paternal Care** | | | | |
| PBI_CARE_P1 | Spoke to me in a warm and friendly voice | | | |
| PBI_CARE_P2 | Did not help me as much as I needed | | | |
| PBI_CARE_P9 | Seemed emotionally cold to me | | | |
| PBI_CARE_P3 | Appeared to understand my problems and worries | | | |
| PBI_CARE_P4 | Was affectionate to me | | | |
| PBI_CARE_P5 | Enjoyed talking things over with me | | | |
| PBI_CARE_P6 | Frequently smiled at me | | | |
| PBI_CARE_P10 | Did not seem to understand what I needed or wanted | | | |
| PBI_CARE_P11 | Made me feel I wasn’t wanted | | | |
| PBI_CARE_P7 | Could make me feel better when I was upset | | | |
| PBI_CARE_P8 | Did not talk with me very much | | | |
| PBI_CARE_P12 | Did not praise me | | | |
| **Maternal Care** | | | | |
| PBI_CARE_M1 | Spoke to me in a warm and friendly voice | | | |
| PBI_CARE_M2 | Did not help me as much as I needed | | | |
| PBI_CARE_M9 | Seemed emotionally cold to me | | | |
| PBI_CARE_M3 | Appeared to understand my problems and worries | | | |
| PBI_CARE_M4 | Was affectionate to me | | | |
| PBI_CARE_M5 | Enjoyed talking things over with me | | | |
| PBI_CARE_M6 | Frequently smiled at me | | | |
| PBI_CARE_M10 | Did not seem to understand what I needed or wanted | | | |
| PBI_CARE_M11 | Made me feel I wasn’t wanted | | | |
| PBI_CARE_M7 | Could make me feel better when I was upset | | | |
| PBI_CARE_M8 | Did not talk with me very much | | | |
| PBI_CARE_M12 | Did not praise me | | | |
| **Paternal Overprotection** | | | | |
| PBI_OVERPR_P1 | Let me do things I liked doing | | | |
| PBI_OVERPR_P2 | Liked me to make my own decisions | | | |
| PBI_OVERPR_P7 | Did not want me to grow up | | | |
| PBI_OVERPR_P8 | Tried to control everything I did | | | |
| PBI_OVERPR_P9 | Invaded my privacy | | | |
| PBI_OVERPR_P10 | Tended to baby me | | | |
| PBI_OVERPR_P3 | Let me decide things for myself | | | |
| PBI_OVERPR_P11 | Tried to make me feel dependent on her/him | | | |
| PBI_OVERPR_P12 | Felt I could not look after myself unless she/he was around | | | |
| PBI_OVERPR_P4 | Gave me as much freedom as I wanted | | | |
| PBI_OVERPR_P5 | Let me go out as often as I wanted | | | |
| PBI_OVERPR_P13 | Was overprotective of me | | | |
| PBI_OVERPR_P6 | Let me dress in any way I pleased | | | |
| **Maternal Overprotection** | | | | |
| PBI_OVERPR_M1 | Let me do things I liked doing | | | |
| PBI_OVERPR_M2 | Liked me to make my own decisions | | | |
| PBI_OVERPR_M7 | Did not want me to grow up | | | |
| PBI_OVERPR_M8 | Tried to control everything I did | | | |
| PBI_OVERPR_M9 | Invaded my privacy | | | |
| PBI_OVERPR_M10 | Tended to baby me | | | |
| PBI_OVERPR_M3 | Let me decide things for myself | | | |
| PBI_OVERPR_M11 | Tried to make me feel dependent on her/him | | | |
| PBI_OVERPR_M12 | Felt I could not look after myself unless she/he was around | | | |
| PBI_OVERPR_M4 | Gave me as much freedom as I wanted | | | |
| PBI_OVERPR_M5 | Let me go out as often as I wanted | | | |
| PBI_OVERPR_M13 | Was overprotective of me | | | |
| PBI_OVERPR_M6 | Let me dress in any way I pleased | | | |
| **Support** | | | | |
| Support1 | Adults could go to with problems/discuss feelings | | | |
| Support2 | Peers to discuss problems and feelings | | | |
| **Family Environment** | | | | |
| FAMILYPROT1 | Visible rooms of the house clean | | | |
| FAMILYPROT2 | Ways to have fun at home | | | |
| FAMILYPROT3 | Lived in a happy home | | | |
| FAMILYPROT4 | Home overly chaotic or noisy | | | |
| **Positive Childhood Experiences** | | | | |
| PCE1 | Capable of discussing feelings with family | | | |
| PCE2 | Supported by family during tough times | | | |
| PCE3 | Enjoyed community traditions | | | |
| PCE4 | Sense of belonging in high school | | | |
| PCE5 | Felt supported by friends | | | |
| PCE6 | At least two caring adults (not parents) | | | |
| PCE7 | Felt safe and protected by an adult at home | | | |
| **Benevolent Childhood Experiences** | | | | |
| BCE1 | At least one caregiver with whom feel safe | | | |
| BCE2 | At least one good friend | | | |
| BCE3 | Beliefs that gave you comfort | | | |
| BCE_4.1 | Liked school | | | |
| BCE_4.2 | Liked high school | | | |
| BCE5 | At least one teacher that cared | | | |
| BCE6 | Good neighbors | | | |
| BCE7 | Adult who provided support or advice | | | |
| BCE8 | Opportunities to have a good time | | | |
| BCE9 | Like yourself or feel comfortable with yourself | | | |
| BCE10 | Had a predictable home routine | | | |

CTQ: Childhood Trauma Questionnaire; RR: Role Reversal; PA_P: Psychological Abuse Father; PA_M: Psychological Abuse Mother; PBI_CARE_F: Paternal care; PBI_CARE_M: Maternal care; PBI_OVERPR_F: Overprotection father; PBI_OVERPR_M: Overprotection mother; FAMILY_PROT: Family protective factors; PCE: Positive Childhood Experiences; BCE: Benevolent Childhood Experiences.

# **Table S2.** Descriptive statistics for study variables (n=1181).

|  |  | *Descriptive statistics* | |  |
| --- | --- | --- | --- | --- |
|  |  | M (SD) | Range | Reliability (α) |
| **Early Exposome** | **F1 Positive Experiences** | -0.04 (-0.77) | -2.86 - 1.91 | - |
|  | **F2 Paternal Adversity** | 0.06 (0.82) | -3.14 - 2.73 | - |
|  | **F3 Maternal Adversity** | 0.03 (0.84) | -3.38 - 2.70 | - |
|  | **F4 Role Reversal** | 0.04 (0.82) | -2.25 - 2.50 | - |
|  | **Early Exposome Factor** | 0.02 (0.91) | -2.22 - 2.77 | - |
| **Environmental Measures** | **Childhood Adversity** |  |  | 0.89 |
|  | **Emotional Abuse** | 8.83(4.30) | 5.00 – 25.00 | 0.83 |
|  | **Physical Abuse** | 5.64(1.64) | 5.00 – 20.00 | 0.73 |
|  | **Sexual Abuse** | 5.91(2.76) | 5.00 – 25.00 | 0.92 |
|  | **Emotional Neglect** | 10.41(4.28) | 5.00 – 25.00 | 0.87 |
|  | **Physical Neglect** | 6.26(1.92) | 5.00 – 19.00 | 0.45 |
|  | **Discord** | 5.54(3.92) | 0.00 – 15.00 | 0.40 |
|  | **Violence** | 0.29(0.76) | 0.00 – 6.00 | 0.55 |
|  | **Role Reversal** | 11.61(7.09) | 0.00 – 32.00 | 0.79 |
|  | **Paternal Care** | 23.09(8.36) | 0.00 – 36.00 | 0.88 |
|  | **Maternal Care** | 26.62(7.34) | 0.00 – 36.00 | 0.92 |
|  | **Paternal Overprotection** | 13.38(5.64) | 2.00 – 34.00 | 0.86 |
|  | **Maternal Overprotection** | 14.33(7.31) | 0.00 – 38.00 | 0.88 |
|  | **Paternal Antipathy** | 2.29(2.80) | 0.00 – 12.00 | 0.74 |
|  | **Maternal Antipathy** | 1.74(2.77) | 0.00 – 12.00 | 0.81 |
|  | **Paternal Psychological Abuse** | 2.05(3.19) | 0.00 – 16.00 | 0.82 |
|  | **Maternal Psychological Abuse** | 3.17(3.65) | 0.00 – 17.00 | 0.78 |
|  | **Childhood Positive Experiences** |  |  |  |
|  | **Support** | 1.50(0.68) | 0.00 – 2.00 | 0.40 |
|  | **Family Protective Factors** | 4.18(0.73) | 1.25 – 5.00 | 0.73 |
|  | **Positive Childhood Experiences** | 5.28(1.87) | 0.00 – 7.00 | 0.62 |
|  | **Benevolent Childhood Experiences** | 7.60(1.86) | 0.00 – 10.00 | 0.75 |
| **Main Outcome Domains** | **P-factor** | 0.02 (0.92) | -2.52 - 2.59 | - |
|  | **Positive Mental Health** | 0.00 (0.94) | -2.66 - 3.12 | - |
|  | **Functioning** | 8.64 (3.75) | 0.00 - 21.00 | 0.61 |
| **Individual Outcome Measures** | **Positive Schizotypy** | 2.74 (2.68) | 0.00 - 12.00 | 0.77 |
|  | **Negative Schizotypy** | 2.11 (2.14) | 0.00 - 12.00 | 0.70 |
|  | **Disorganized Schizotypy** | 3.56 (3.54) | 0.00 - 12.00 | 0.88 |
|  | **Psychotic-like Experiences (PLE)** | 30.22 (6.31) | 20.00 - 58.00 | 0.84 |
|  | **Suspiciousness** | 3.30 (2.27) | 0.00 - 8.00 | 0.77 |
|  | **Ideas of Reference** | 3.08 (2.33) | 0.00 - 9.00 | 0.75 |
|  | **Depression** | 13.76 (10.14) | 0.00 - 56.00 | 0.91 |
|  | **Anxiety** | 11.72 (9.12) | 0.00 - 49.00 | 0.90 |
|  | **Well-being** | 48.48 (9.51) | 15.00 - 117.00 | 0.81 |
|  | **Self-esteem** | 18.15 (5.91) | 2.00 - 30.00 | 0.88 |
|  | **Resilience** | 27.29 (6.99) | 3.00 - 41.00 | 0.85 |

# **Table S3.** Pearson correlations for study variables.

|  | **2** | **3** | **4** | **5** | **6** | **7** | **8** |
| --- | --- | --- | --- | --- | --- | --- | --- |
| 1. **F1 Positive Experiences** | -.01 | .05 | .06^*^ | -.05 | -.18^**^ | .17^**^ | -.21^**^ |
| 1. **F2 Paternal Adversity** |  | -.06^*^ | -.07^*^ | .11^**^ | .08^**^ | -.06^*^ | .12^**^ |
| 1. **F3 Maternal Adversity** |  |  | .03 | .10^**^ | .13^**^ | -.07^*^ | .06^*^ |
| 1. **F4 Role Reversal** |  |  |  | .03 | .04 | .05 | -.02 |
| 1. **Early Exposome Factor** |  |  |  |  | **.42^**^** | **-.38^**^** | ***.50^**^*** |
| 1. **P-factor** |  |  |  |  |  | ***-.66^**^*** | ***.61^**^*** |
| 1. **Positive Mental Health** |  |  |  |  |  |  | ***-.60^**^*** |
| 1. **Functioning** |  |  |  |  |  |  |  |

|  | **9** | **10** | **11** | **12** | **13** | **14** | **15** | **16** | **17** | **18** | **19** |
| --- | --- | --- | --- | --- | --- | --- | --- | --- | --- | --- | --- |
| 1. **F1 Positive Experiences** | -.03 | -.16^**^ | -.15^**^ | -.06^*^ | -.18^**^ | -.04 | -.16^**^ | -.12^**^ | .16^**^ | .17^**^ | .16^**^ |
| 1. **F2 Paternal Adversity** | .02 | .01 | .07^*^ | .06^*^ | .06^*^ | .06^*^ | .06 | .01 | -.06^*^ | -.04 | -.10^**^ |
| 1. **F3 Maternal Adversity** | .10^**^ | .04 | .12^**^ | .08^**^ | .09^**^ | .08^**^ | .07^*^ | .05 | -.03 | -.03 | -.04 |
| 1. **F4 Role Reversal** | .12^**^ | .00 | .02 | .09^**^ | .04 | .05 | .04 | .07^*^ | .05 | .04 | .11^**^ |
| 1. **Early Exposome Factor** | .18^**^ | .23^**^ | **.36^**^** | .23^**^ | **.32^**^** | .19^**^ | **.42^**^** | **.34^**^** | **-.41^**^** | **-.35^**^** | -.27^**^ |
| 1. **P-factor** | **.48^**^** | **.31^**^** | ***.86^**^*** | ***.64^**^*** | ***.75^**^*** | ***.62^**^*** | ***.73^**^*** | ***.59^**^*** | ***-.59^**^*** | ***-.61^**^*** | **-.49^**^** |
| 1. **Positive Mental Health** | -.19^**^ | -.14^**^ | ***-.53^**^*** | -.25^**^ | **-.42^**^** | **-.30^**^** | ***-.73^**^*** | ***-.54^**^*** | ***.76^**^*** | ***.90^**^*** | ***.73^**^*** |
| 1. **Functioning** | -.21^**^ | -.28^**^ | ***-.50^**^*** | -.29^**^ | **-.45^**^** | -.29^**^ | ***-.62^**^*** | **-.45^**^** | ***.64^**^*** | ***.53^**^*** | **.45^**^** |
| 1. **Positive Schizotypy** |  | .10^**^ | **.36^**^** | ***.74^**^*** | **.43^**^** | ***.59^**^*** | .30^**^ | **.36^**^** | -.14^**^ | -.18^**^ | -.09^**^ |
| 1. **Negative Schizotypy** |  |  | **.31^**^** | .18^**^ | .22^**^ | .11^**^ | .23^**^ | .09^**^ | -.27^**^ | -.15^**^ | -.12^**^ |
| 1. **Disorganized Schizotypy** |  |  |  | **.47^**^** | ***.53^**^*** | **.42^**^** | ***.59^**^*** | **.50^**^** | **-.49^**^** | **-.48^**^** | **-.41^**^** |
| 1. **Psychotic-like Experiences** |  |  |  |  | ***.55^**^*** | ***.65^**^*** | **.38^**^** | **.42^**^** | -.20^**^ | -.25^**^ | -.14^**^ |
| 1. **Suspiciousness** |  |  |  |  |  | ***.56^**^*** | **.*50^**^*** | **.45^**^** | **-.40^**^** | **-.41^**^** | -.30^**^ |
| 1. **Ideas of Reference** |  |  |  |  |  |  | **.35^**^** | **.36^**^** | -.27^**^ | -.30^**^ | -.20^**^ |
| 1. **Depression** |  |  |  |  |  |  |  | ***.69^**^*** | ***-.69^**^*** | ***-.65^**^*** | ***-.54^**^*** |
| 1. **Anxiety** |  |  |  |  |  |  |  |  | **-.50^**^** | **-.47^**^** | **-.42^**^** |
| 1. **Well-being** |  |  |  |  |  |  |  |  |  | ***.60^**^*** | ***.59^**^*** |
| 1. **Self-esteem** |  |  |  |  |  |  |  |  |  |  | ***.58^**^*** |
| 1. **Resilience** |  |  |  |  |  |  |  |  |  |  |  |

Moderate correlations are in bold (*r* ≥ .30), large correlations (*r* => .50) are in bold and italics (Cohen, 1988).

# **Table S4.** Factor loadings of the first exploratory factor analysis of the initial collection of Early Exposome items (n = 1.181).

|  | | | | | |  |  |  |
| --- | --- | --- | --- | --- | --- | --- | --- | --- |
| **Item** | **Content** | **FAC1** | **FAC2** | **FAC3** | **FAC4** | **FAC 5** | **FAC 6** | **FAC 7** |
| PCE2_R | Supported by family during tough times | **-0.39** | -0.29 | 0.00 | 0.06 | -0.17 | 0.05 | 0.34 |
| CTQ_3 | Family called me things like “stupid”, “lazy” or “ugly” | **0.36** | 0.30 | 0.21 | -0.01 | 0.18 | -0.03 | 0.10 |
| CTQ_8 | Thought parents wished I had never been born | **0.33** | 0.22 | 0.28 | -0.08 | 0.13 | -0.06 | 0.00 |
| ANT_MOTHER1 | Was difficult to please | **0.74** | 0.04 | 0.01 | -0.02 | -0.03 | -0.16 | 0.05 |
| ANT_MOTHER2 | Made me feel unwanted | **0.74** | -0.03 | 0.06 | -0.05 | 0.09 | 0.00 | -0.16 |
| ANT_MOTHER3 | Was critical | **0.82** | 0.02 | 0.00 | -0.09 | 0.03 | -0.16 | 0.12 |
| ANT_MOTHER4 | Made me feel I was a nuisance | **0.76** | 0.10 | 0.18 | -0.11 | 0.02 | 0.04 | 0.01 |
| ANT_MOTHER5 | Picked on me unfairly | **0.72** | -0.10 | 0.21 | -0.05 | 0.06 | 0.05 | 0.10 |
| ANT_MOTHER6 | Did not like me as much as my siblings | **0.56** | 0.07 | 0.04 | -0.13 | 0.09 | 0.02 | -0.13 |
| PA_M1 | Undermined my confidence | **0.73** | 0.10 | -0.02 | 0.04 | -0.01 | -0.06 | 0.12 |
| PA_M2 | Confuse me by telling contradictory things | **0.61** | 0.01 | 0.08 | 0.12 | -0.01 | -0.06 | 0.10 |
| PA_M3 | Played on my fears | **0.73** | 0.01 | 0.06 | 0.05 | 0.10 | -0.05 | 0.20 |
| PA_M4 | Humiliated me, put me down | **0.77** | 0.00 | 0.16 | -0.07 | 0.07 | -0.01 | 0.17 |
| PA_M5 | Shamed me in front of others | **0.57** | 0.00 | 0.05 | 0.09 | 0.09 | -0.03 | 0.07 |
| PA_M6 | Was rejecting | **0.73** | 0.05 | 0.05 | -0.10 | -0.01 | 0.01 | -0.13 |
| PA_M7 | Deprivation of light, food, or company | **0.33** | 0.01 | 0.23 | 0.06 | 0.31 | 0.07 | 0.05 |
| PA_M8 | Made me feel guilty, so I would do what I was told | **0.71** | -0.05 | 0.08 | 0.10 | 0.03 | -0.05 | 0.14 |
| PA_M9 | Threatened to hurt loved ones to get what she wanted | **0.55** | -0.24 | 0.34 | -0.09 | 0.09 | 0.16 | 0.06 |
| PA_M11 | Said wanted me dead | **0.57** | -0.14 | 0.37 | -0.08 | 0.12 | -0.05 | -0.01 |
| CARE_M1 | Spoke in a warm and friendly voice | **-0.71** | -0.02 | -0.03 | -0.05 | 0.04 | -0.06 | 0.17 |
| CARE_M2 | Not helped me as much as I needed | **0.45** | 0.14 | 0.12 | -0.03 | 0.06 | 0.10 | -0.22 |
| CARE_M3 | Understood my problems and worries | **-0.71** | -0.09 | 0.06 | -0.04 | -0.04 | -0.03 | 0.16 |
| CARE_M4 | Was affectionate to me | **-0.62** | -0.10 | -0.06 | -0.03 | 0.11 | -0.09 | 0.27 |
| CARE_M5 | Enjoyed talking things over with me | **-0.73** | -0.12 | 0.07 | 0.01 | 0.08 | -0.09 | 0.17 |
| CARE_M6 | Frequently smiled at me | **-0.66** | -0.10 | -0.02 | 0.00 | 0.06 | 0.00 | 0.19 |
| CARE_M7 | Made me feel better when I was upset | **-0.58** | -0.06 | 0.10 | -0.04 | 0.01 | -0.11 | 0.20 |
| CARE_M8 | Did not talk with me very much | **0.60** | 0.13 | -0.01 | 0.01 | -0.10 | 0.09 | -0.27 |
| CARE_M9 | Emotionally cold to me | **0.66** | 0.04 | 0.06 | 0.04 | -0.07 | 0.08 | -0.22 |
| CARE_M10 | Did not understand what I needed or wanted | **0.70** | 0.12 | -0.07 | 0.08 | 0.04 | -0.01 | -0.13 |
| CARE_M11 | Made me feel I wasn’t wanted | **0.74** | 0.09 | 0.04 | -0.07 | 0.03 | 0.05 | -0.17 |
| CARE_M12 | Did not praise me | **0.63** | 0.19 | -0.05 | 0.00 | -0.02 | 0.02 | -0.18 |
| OVERPR_M2 | Liked me to make my own decisions | **-0.61** | -0.07 | 0.10 | -0.32 | -0.01 | -0.02 | -0.13 |
| OVERPR_M8 | Tried to control everything I did | **0.58** | -0.08 | -0.14 | 0.40 | 0.07 | -0.02 | 0.26 |
| OVERPR_M9 | Invaded my privacy | **0.60** | -0.06 | -0.07 | 0.38 | 0.06 | -0.06 | 0.18 |
| OVERPR_M10 | Tended to baby me | **0.41** | -0.03 | -0.11 | 0.34 | 0.02 | -0.03 | 0.21 |
| OVERPR_M3 | Let me decide things for myself | **-0.53** | -0.05 | 0.20 | -0.39 | -0.05 | 0.01 | -0.16 |
| OVERPR_M11 | Tried to make me feel dependent on her | **0.54** | 0.01 | 0.01 | 0.34 | 0.05 | 0.06 | 0.17 |
| OVERPR_M6 | Let me dress in any way I pleased | **-0.43** | 0.07 | 0.15 | -0.42 | -0.06 | 0.01 | -0.04 |
| Support1R | Adults could go to with problems/discuss feelings | -0.34 | **-0.37** | 0.04 | 0.03 | -0.14 | -0.10 | 0.33 |
| PCE6_R | At least two caring adults (not parents) | -0.18 | **-0.26** | -0.11 | 0.03 | -0.12 | 0.12 | 0.24 |
| CTQ_19 | Felt that someone in my family hated me | 0.20 | **0.31** | 0.26 | -0.04 | 0.20 | -0.06 | 0.06 |
| CTQ_29 | Family was source of strength | -0.32 | **-0.36** | -0.20 | -0.05 | -0.14 | -0.02 | 0.27 |
| ANT_FATHER1 | Was difficult to please | -0.02 | **0.71** | 0.07 | 0.14 | 0.02 | -0.14 | 0.05 |
| ANT_FATHER2 | Was critical of me | -0.01 | **0.76** | 0.04 | 0.10 | 0.02 | -0.18 | 0.06 |
| ANT_FATHER3 | Made me feel I was a nuisance | 0.03 | **0.77** | 0.14 | -0.05 | 0.02 | -0.11 | 0.06 |
| ANT_FATHER4 | Picked on me unfairly | -0.05 | **0.61** | 0.19 | 0.14 | 0.01 | -0.03 | 0.03 |
| ANT_FATHER5 | Was there if I needed | -0.03 | **0.46** | -0.04 | -0.15 | 0.01 | -0.01 | -0.05 |
| ANT_FATHER6 | Did not like me as much as my siblings | 0.11 | **0.55** | -0.02 | 0.06 | -0.06 | 0.03 | -0.12 |
| PA_P1 | Undermined my confidence | -0.05 | **0.64** | 0.11 | 0.17 | -0.01 | -0.16 | 0.07 |
| PA_P2 | Played on my fears | -0.03 | **0.56** | 0.18 | 0.25 | 0.07 | 0.01 | 0.05 |
| PA_P3 | Liked to see me suffer | -0.03 | **0.56** | 0.17 | 0.13 | -0.07 | -0.23 | 0.08 |
| PA_P4 | Humiliated me, put me down. | 0.01 | **0.70** | 0.19 | 0.10 | -0.04 | -0.17 | 0.17 |
| PA_P5 | Shamed me in front of others | 0.08 | **0.45** | 0.10 | 0.16 | 0.00 | -0.19 | 0.15 |
| PA_P6 | Was rejecting | 0.08 | **0.74** | 0.10 | -0.04 | 0.04 | -0.14 | 0.15 |
| PA_P7 | Took away things I cherished | 0.05 | **0.43** | 0.15 | 0.26 | 0.03 | -0.21 | 0.12 |
| PA_P8 | Made me feel guilty, so I would do what I was told | -0.05 | **0.45** | 0.24 | 0.28 | 0.05 | -0.11 | 0.13 |
| CARE_P1 | Spoke in a warm and friendly voice | 0.11 | **-0.81** | -0.01 | -0.10 | -0.04 | -0.09 | 0.07 |
| CARE_P2 | Did not help me as much as I needed | 0.15 | **0.55** | -0.02 | -0.05 | 0.04 | -0.02 | -0.08 |
| CARE_P3 | Understood my problems and worries | -0.04 | **-0.73** | 0.08 | -0.05 | -0.09 | 0.00 | 0.09 |
| CARE_P4 | Was affectionate to me | -0.06 | **-0.81** | 0.04 | 0.07 | 0.02 | -0.09 | 0.04 |
| CARE_P5 | Enjoyed talking things over with me | -0.03 | **-0.82** | 0.11 | 0.00 | 0.06 | 0.07 | 0.02 |
| CARE_P6 | Frequently smiled at me | -0.04 | **-0.78** | 0.02 | -0.07 | 0.06 | -0.09 | 0.05 |
| CARE_P7 | Made me feel better when I was upset | -0.02 | **-0.71** | 0.03 | 0.01 | 0.00 | -0.07 | 0.09 |
| CARE_P8 | Did not talk with me very much | 0.08 | **0.78** | -0.10 | -0.04 | 0.05 | 0.06 | 0.00 |
| CARE_P9 | Emotionally cold to me | 0.08 | **0.78** | -0.11 | -0.01 | 0.05 | 0.06 | -0.03 |
| CARE_P10 | Did not understand what I needed or wanted | 0.07 | **0.66** | 0.03 | 0.16 | 0.01 | -0.06 | -0.08 |
| CARE_P11 | Made me feel I wasn’t wanted | -0.01 | **0.76** | 0.09 | 0.01 | -0.02 | 0.00 | -0.06 |
| CARE_P12 | Did not praise me | 0.07 | **0.78** | -0.11 | 0.00 | 0.02 | 0.00 | -0.08 |
| BCE10_R | Had a predictable home routine | -0.10 | 0.01 | **-0.46** | 0.11 | -0.09 | 0.36 | 0.15 |
| CTQ_1 | Not enough food to eat | -0.03 | -0.11 | **0.50** | 0.06 | 0.26 | 0.11 | -0.31 |
| CTQ_4 | Parents too drunk or to high | -0.08 | 0.13 | **0.52** | -0.09 | 0.17 | 0.09 | 0.01 |
| CTQ_6 | Wore dirty clothes | 0.01 | -0.16 | **0.45** | 0.00 | 0.15 | 0.01 | -0.22 |
| CTQ_9 | Got hit by family member so hard that I had to go to the doctor | 0.09 | 0.13 | **0.35** | 0.04 | 0.15 | -0.16 | 0.15 |
| CTQ_11 | Family member hit so hard bruises or marks | 0.19 | 0.23 | **0.34** | -0.02 | 0.14 | 0.05 | 0.11 |
| CTQ_12 | Punished with belt, board, cord or hard object | 0.15 | 0.11 | **0.30** | 0.12 | 0.08 | 0.10 | 0.08 |
| CTQ_13 | People in family looked out for each other | -0.21 | -0.28 | **-0.32** | -0.05 | -0.07 | 0.01 | 0.23 |
| CTQ_14 | Family said hurtful or insulting things | 0.20 | 0.29 | **0.38** | 0.02 | 0.20 | -0.01 | 0.15 |
| CTQ_16 | Think physically abused | 0.17 | 0.24 | **0.32** | 0.04 | 0.29 | 0.02 | 0.10 |
| CTQ_20 | Family felt close | -0.18 | -0.29 | **-0.31** | -0.06 | -0.02 | 0.03 | 0.25 |
| RR_1R | Lot of responsibility at home | 0.02 | -0.15 | **0.54** | 0.21 | -0.01 | 0.06 | -0.34 |
| RR_2R | Expected to do a lot of housework | 0.08 | -0.13 | **0.48** | 0.20 | -0.03 | 0.06 | -0.35 |
| RR_3R | Had to look after younger siblings | 0.08 | -0.11 | **0.40** | 0.16 | 0.00 | 0.08 | -0.25 |
| RR_4R | Missed out seeing friends because of home responsibilities | 0.09 | -0.11 | **0.42** | 0.29 | 0.02 | 0.07 | -0.25 |
| RR_5R | Parent/s look to you for help as a child | 0.04 | -0.22 | **0.54** | 0.10 | 0.03 | 0.00 | -0.08 |
| RR_6R | Parent/s confided their problems in you | -0.03 | -0.22 | **0.55** | 0.02 | 0.01 | -0.08 | 0.10 |
| RR_8R | Parent/s rely for emotional support as a child | 0.20 | -0.01 | **0.48** | 0.15 | -0.03 | -0.07 | 0.09 |
| RR_9R | Felt concerned and worried about parents | -0.01 | -0.07 | **0.53** | 0.10 | 0.06 | -0.09 | 0.06 |
| FAMPROTEC_1 | Visible rooms of the house clean | 0.05 | 0.05 | **-0.38** | -0.03 | -0.07 | -0.01 | 0.32 |
| FAMPROTEC_2 | Ways to have fun at home | -0.22 | -0.29 | **-0.31** | -0.06 | -0.07 | 0.05 | 0.26 |
| FAMPROTEC_3 | Lived in a happy home | -0.18 | -0.33 | **-0.45** | -0.07 | -0.05 | 0.02 | 0.18 |
| FAMPROTEC_4 | Home overly chaotic or noisy | -0.10 | -0.08 | **-0.45** | -0.04 | -0.01 | -0.03 | 0.08 |
| OVERPR_M4 | Gave me as much freedom as I wanted | -0.05 | 0.08 | **0.26** | -0.22 | -0.13 | 0.08 | -0.25 |
| Discord_1 | Periods relatives (not parents) stopped talking to each other/argued a lot | 0.13 | 0.02 | **0.29** | 0.11 | 0.08 | -0.01 | 0.00 |
| Discord_2 | Periods parents stopped talking to each other/argued a lot | 0.16 | 0.16 | **0.56** | 0.08 | 0.01 | 0.01 | 0.12 |
| Discord_3 | Parents raised their voices | 0.18 | 0.18 | **0.47** | 0.07 | 0.05 | -0.01 | 0.18 |
| Discord_4 | Parents intense arguments throwing things | 0.07 | 0.19 | **0.70** | -0.04 | -0.03 | 0.06 | 0.12 |
| Discord_5 | Siblings and self often present/involved in parent's arguments | 0.11 | 0.13 | **0.53** | 0.00 | 0.01 | 0.00 | 0.18 |
| Violence_1 | Violence between parent throwing things/physical contact | -0.06 | 0.11 | **0.50** | -0.14 | -0.02 | 0.09 | 0.10 |
| Violence_2 | Violence with consequences hospital, police, social services | -0.15 | 0.26 | **0.60** | -0.18 | 0.14 | 0.11 | -0.03 |
| OVERPR_P1 | Let me do things I liked doing | 0.12 | -0.31 | -0.01 | **-0.59** | -0.04 | -0.04 | 0.19 |
| OVERPR_P2 | Liked me to make my own decisions | 0.06 | -0.38 | -0.01 | **-0.57** | -0.01 | -0.06 | 0.10 |
| OVERPR_P7 | Did not want me to grow up | 0.06 | 0.01 | 0.12 | **0.44** | -0.09 | 0.03 | -0.01 |
| OVERPR_P8 | Tried to control everything I did | -0.13 | 0.19 | 0.09 | **0.71** | -0.03 | 0.05 | -0.04 |
| OVERPR_P9 | Invaded my privacy | -0.03 | 0.10 | 0.13 | **0.64** | 0.01 | 0.01 | -0.03 |
| OVERPR_P10 | Tended to baby me | 0.08 | 0.11 | 0.03 | **0.57** | -0.13 | -0.02 | -0.01 |
| OVERPR_P3 | Let me decide things for myself | 0.04 | -0.35 | 0.02 | **-0.57** | -0.03 | 0.01 | 0.03 |
| OVERPR_P11 | Tried to make me feel dependent on him | -0.05 | 0.22 | 0.17 | **0.59** | -0.07 | 0.05 | 0.03 |
| OVERPR_P12 | Felt I could not look after myself unless she/he was around | 0.09 | 0.07 | 0.09 | **0.53** | -0.07 | 0.09 | 0.01 |
| OVERPR_P4 | Gave me as much freedom as I wanted | 0.24 | 0.07 | 0.09 | **-0.32** | -0.08 | 0.10 | 0.00 |
| OVERPR_P5 | Let me go out as often as I wanted | 0.12 | -0.02 | 0.08 | **-0.65** | -0.08 | -0.01 | 0.10 |
| OVERPR_P13 | Was overprotective of me | -0.08 | -0.10 | 0.08 | **0.69** | -0.02 | 0.00 | 0.03 |
| OVERPR_P6 | Let me dress in any way I pleased | -0.03 | -0.09 | -0.01 | **-0.57** | -0.06 | -0.05 | 0.15 |
| OVERPR_M1 | Let me do things I liked doing | -0.33 | 0.02 | 0.12 | **-0.46** | -0.10 | -0.04 | 0.06 |
| OVERPR_M7 | Did not want me to grow up | 0.25 | 0.03 | 0.09 | **0.28** | 0.03 | 0.04 | 0.17 |
| OVERPR_M12 | Felt I could not look after myself unless she/he was around | 0.29 | 0.10 | -0.07 | **0.31** | 0.05 | -0.01 | 0.22 |
| OVERPR_M5 | Let me go out as often as I wanted | -0.13 | 0.12 | 0.18 | **-0.49** | -0.16 | -0.02 | -0.09 |
| OVERPR_M13 | Was overprotective of me | 0.29 | -0.09 | -0.13 | **0.49** | 0.04 | -0.07 | 0.29 |
| CTQ_18 | Someone hit so hard someone noticed (teacher, doctor, neighbor) | 0.09 | 0.11 | 0.26 | -0.05 | **0.29** | 0.15 | 0.01 |
| CTQ_21 | Someone tried to touch/make touch them in a sexual way | -0.07 | -0.04 | -0.08 | 0.03 | **0.93** | 0.08 | 0.00 |
| CTQ_22 | Someone threatened to hurt/tell lies about me unless I did something sexual with them | 0.02 | -0.13 | 0.09 | 0.00 | **0.74** | -0.11 | 0.02 |
| CTQ_24 | Someone tried to make me do sexual things or watch sexual things | -0.13 | -0.05 | -0.02 | 0.01 | **0.93** | 0.10 | 0.00 |
| CTQ_25 | Someone molested me | -0.03 | 0.03 | -0.02 | -0.02 | **0.91** | 0.03 | -0.01 |
| CTQ_26 | Was emotionally abused | 0.25 | 0.26 | 0.32 | 0.04 | **0.35** | -0.08 | 0.12 |
| CTQ_28 | I believe that I was sexually abused | -0.02 | 0.02 | -0.05 | -0.08 | **0.87** | -0.08 | 0.06 |
| Support2R | Peers to discuss problems and feelings | -0.09 | -0.03 | -0.04 | -0.09 | 0.06 | **0.56** | 0.39 |
| BCE1_R | Caregiver provided sense of safety | -0.17 | -0.15 | -0.04 | 0.03 | -0.05 | **0.36** | 0.25 |
| BCE2_R | At least one good friend | -0.12 | 0.10 | -0.04 | -0.15 | 0.02 | **0.62** | 0.31 |
| BCE_41R | Liked school | -0.12 | -0.07 | -0.08 | -0.03 | -0.13 | **0.53** | 0.08 |
| BCE_42R | Liked high school | -0.16 | -0.02 | 0.02 | -0.02 | -0.21 | **0.60** | 0.18 |
| BCE5_R | At least one teacher that cared | 0.00 | -0.12 | 0.00 | -0.05 | -0.08 | **0.44** | 0.25 |
| BCE6 | Good neighbors | 0.01 | -0.12 | -0.21 | -0.02 | 0.02 | **0.39** | 0.18 |
| PCE4_R | Sense of belonging in high school | -0.19 | -0.03 | 0.06 | -0.09 | -0.27 | **0.35** | 0.32 |
| PCE5_R | Felt supported by friends | -0.10 | 0.03 | 0.00 | -0.12 | -0.13 | **0.62** | 0.31 |
| PCE7_R | Felt safe and protected by an adult at home | -0.30 | -0.23 | -0.26 | 0.10 | -0.19 | **0.34** | 0.28 |
| BCE9 | Like yourself or feel comfortable with yourself | -0.16 | -0.13 | -0.07 | -0.07 | -0.20 | **0.21** | 0.21 |
| CTQ_27 | Someone take to doctor if I needed it | -0.17 | -0.10 | -0.14 | 0.05 | -0.07 | **0.18** | 0.15 |
| PA_M10 | Forced me to steal or break the law | 0.10 | -0.03 | 0.06 | 0.08 | 0.07 | **1.30** | -0.11 |
| BCE3 | Beliefs that gave you comfort | 0.03 | -0.13 | 0.02 | 0.04 | -0.08 | 0.12 | **0.22** |
| BCE7_R | Adult who provided support or advice | -0.14 | -0.20 | 0.02 | -0.02 | -0.09 | 0.09 | **0.38** |
| PCE1_R | Capable of discussing feelings with family | -0.31 | -0.30 | 0.10 | 0.00 | -0.12 | -0.01 | **0.41** |
| PCE3_R | Enjoyed community traditions | -0.21 | -0.25 | 0.00 | -0.01 | -0.17 | 0.18 | **0.31** |
| BCE8 | Opportunities to have a good time | -0.05 | -0.05 | -0.07 | -0.21 | -0.23 | 0.32 | **0.38** |
| CTQ_2 | Family member took care/protected me | -0.21 | -0.19 | -0.29 | 0.03 | -0.04 | 0.08 | **0.37** |
| CTQ_5 | Family member made feel important or special | -0.36 | -0.26 | 0.03 | -0.04 | -0.08 | -0.02 | **0.38** |
| CTQ_7 | Felt loved | -0.34 | -0.36 | -0.16 | 0.00 | -0.10 | 0.05 | **0.36** |
|  |  |  |  |  |  |  |  |  |

Highest factor loadings for a given factor are bolded; Cross-loadings are underlined.

# **Table S5.** Factor loadings of the second exploratory factor analysis for general Early Exposome items not specifying parental figures (n = 1.181).

|  | | | | | |  |  |
| --- | --- | --- | --- | --- | --- | --- | --- |
| **Item** | **Content** | **FAC1** | **FAC2** | **FAC3** | **FAC4** | **FAC5** | **FAC6** |
| CTQ_20 | Family felt close | **0,42** | -0,40 | 0,14 | 0,02 | 0,01 | -0,06 |
| CTQ_13 | People in family looked out for each other | **0,44** | -0,35 | 0,08 | -0,02 | -0,04 | -0,10 |
| FAMILYPROT2 | Ways to have fun at home | **0,47** | -0,29 | 0,13 | 0,03 | -0,09 | -0,12 |
| CTQ_29 | Family was source of strength | **0,62** | -0,23 | 0,06 | -0,07 | -0,13 | -0,08 |
| PCE7_R | Felt safe and protected by an adult at home | **0,62** | -0,18 | 0,12 | -0,12 | -0,07 | -0,11 |
| CTQ_2 | Family member took care of me | **0,52** | -0,14 | 0,09 | 0,04 | -0,09 | -0,19 |
| PCE6_R | At least two caring adults (not parents) | **0,39** | -0,12 | 0,26 | 0,02 | -0,11 | 0,05 |
| CTQ_7 | Felt loved | **0,61** | -0,12 | 0,19 | 0,00 | -0,14 | -0,11 |
| PCE2_R | Supported by family during tough times | **0,73** | -0,05 | 0,11 | -0,07 | -0,02 | 0,00 |
| BCE7_R | Adult who provided support or advice | **0,48** | -0,03 | 0,27 | 0,02 | 0,09 | 0,02 |
| CTQ_27 | Had the perfect childhood. | **0,34** | 0,01 | 0,03 | -0,05 | -0,06 | -0,21 |
| BCE1_R | Caregiver provided sense of safety | **0,49** | 0,04 | 0,14 | -0,01 | -0,01 | -0,03 |
| CTQ_5 | Family member made me feel important | **0,64** | 0,04 | 0,11 | 0,02 | -0,12 | -0,05 |
| Support1R | Adults could go to with problems/discuss feelings | **0,77** | 0,05 | -0,03 | -0,07 | -0,01 | -0,12 |
| PCE1_R | Capable of discussing feelings with family | **0,74** | 0,08 | 0,11 | -0,03 | 0,04 | -0,07 |
| CTQ_8 | Thought parents wished I had never been born | **-0,28** | 0,22 | -0,07 | 0,08 | 0,23 | 0,05 |
| RR_6R | Parent/s confided their problems in you | **0,49** | 0,40 | -0,22 | 0,05 | -0,08 | 0,37 |
| FAMILYPROT3 | Lived in a happy home | 0,40 | **-0,53** | 0,11 | 0,03 | -0,07 | -0,08 |
| BCE10_R | Had a predictable home routine | 0,11 | **-0,37** | 0,19 | -0,13 | 0,15 | -0,24 |
| FAMILYPROT4 | Home overly chaotic or noisy | 0,11 | **-0,34** | -0,03 | 0,00 | -0,09 | -0,24 |
| Discord_1_R | Family members stopped talking/argued a lot | -0,04 | **0,31** | -0,10 | 0,08 | 0,00 | 0,09 |
| RR_9R | Felt concerned and worried about parents | 0,19 | **0,37** | -0,13 | 0,08 | -0,04 | 0,31 |
| RR_8R | Parent/s rely for emotional support as a child | 0,12 | **0,46** | -0,20 | 0,00 | -0,06 | 0,27 |
| Violence_1_R | Violence between parents, throwing thigs or physical contact | 0,04 | **0,54** | 0,14 | -0,06 | 0,10 | -0,01 |
| CTQ_4 | Parents too drunk or to high | -0,15 | **0,61** | 0,18 | 0,13 | 0,01 | 0,02 |
| Discord_5_R | Siblings and I involved in arguments | 0,06 | **0,64** | 0,00 | -0,04 | 0,17 | 0,00 |
| Discord_3_R | Parents raised voice during arguments | -0,07 | **0,67** | -0,02 | 0,01 | 0,09 | -0,07 |
| Discord_2_R | Parents stopped talking/argued a lot | -0,07 | **0,69** | -0,02 | -0,01 | 0,07 | 0,01 |
| Violence_2_R | Consequences of violence between parents, like calling the police, hospital | -0,02 | **0,78** | 0,06 | 0,10 | 0,05 | -0,04 |
| Discord_4_R | Tension in the home | 0,01 | **0,82** | 0,04 | -0,07 | 0,12 | 0,01 |
| BCE6_R | Had good neighbors | 0,17 | -0,22 | **0,23** | 0,04 | 0,07 | -0,06 |
| BCE_4,1R | Enjoyed primary school | 0,02 | -0,08 | **0,53** | -0,06 | -0,05 | 0,06 |
| BCE9_R | Felt good with oneself | 0,13 | -0,07 | **0,48** | -0,08 | -0,11 | 0,03 |
| PCE3_R | Enjoyed community traditions | 0,38 | -0,06 | **0,47** | -0,05 | 0,06 | 0,03 |
| BCE3_R | Beliefs that comforted | 0,18 | -0,04 | **0,25** | -0,02 | 0,12 | 0,04 |
| BCE8_R | Opportunities to have fun | 0,06 | 0,00 | **0,70** | -0,07 | -0,03 | -0,09 |
| BCE_4,2R | Enjoy secondary school | -0,03 | 0,01 | **0,74** | -0,07 | -0,08 | 0,09 |
| BCE5_R | At least one teacher that cared | 0,17 | 0,04 | **0,37** | 0,03 | -0,11 | 0,01 |
| BCE2_R | At least one good friend | 0.00 | 0.04 | **0.75** | 0.15 | 0.00 | -0.01 |
| Support2R | Peers to discuss problems and feelings | 0.19 | 0.06 | **0.58** | 0.16 | 0.03 | -0.10 |
| PCE5_R | Felt supported by friends | 0.00 | 0.07 | **0.77** | -0.01 | -0.03 | 0.00 |
| PCE4_R | Sense of belonging in high school | 0.08 | 0.08 | **0.77** | -0.13 | -0.02 | 0.07 |
| CTQ_22 | Someone threatened to hurt/tell lies about me unless I did something sexual with them | 0.09 | -0.04 | -0.07 | **0.85** | 0.06 | 0.14 |
| CTQ_24 | Someone tried to make me do sexual things or watch sexual things | 0.05 | -0.03 | 0.04 | **0.95** | 0.02 | 0.01 |
| CTQ_25 | Someone molested me | -0.03 | -0.01 | -0.07 | **0.89** | 0.07 | -0.04 |
| CTQ_21 | Someone tried to touch/make touch them in a sexual way | -0.01 | 0.00 | 0.02 | **0.97** | -0.08 | -0.03 |
| CTQ_28 | I believe that I was sexually abused | -0.08 | 0.00 | 0.02 | **0.96** | 0.00 | -0.05 |
| CTQ_18 | Someone hit so hard someone noticed (teacher, doctor, neighbour) | -0.10 | -0.08 | 0.08 | 0.16 | **0.56** | 0.15 |
| CTQ_11 | Family member hit so hard bruises or marks | 0.02 | -0.01 | -0.02 | -0.06 | **0.94** | 0.04 |
| CTQ_12 | Punished with belt, board, cord or hard object | -0.06 | 0.00 | 0.13 | -0.06 | **0.72** | 0.07 |
| CTQ_9 | Got hit by family member so hard that I had to go to the doctor | 0.13 | 0.02 | -0.09 | 0.04 | **0.81** | 0.05 |
| CTQ_16 | Think physically abused | -0.05 | 0.11 | -0.06 | 0.13 | **0.77** | -0.07 |
| CTQ_3 | Family called me things like “stupid”, “lazy” or “ugly” | -0.28 | 0.19 | -0.08 | 0.09 | **0.36** | -0.02 |
| CTQ_14 | Family said hurful or insulting things | -0.12 | 0.25 | -0.06 | 0.06 | **0.55** | -0.01 |
| CTQ_19 | Felt that someone in my family hated me | -0.18 | 0.27 | -0.15 | 0.09 | **0.34** | -0.09 |
| CTQ_26 | Was emotionally abused | -0.16 | 0.29 | -0.15 | 0.26 | **0.33** | -0.02 |
| FAMILYPROT1 | Visible rooms of the house clean | 0.19 | -0.26 | 0.01 | -0.08 | 0.19 | -0.29 |
| RR_4R | Missed out seeing friends because of home responsibilities | -0.08 | -0.15 | 0.01 | -0.01 | 0.20 | **0.74** |
| RR_3R | Had to look after younger siblings | -0.08 | -0.09 | 0.06 | 0.01 | 0.10 | **0.66** |
| RR_2R | Expected to do a lot of housework | -0.13 | -0.03 | 0.04 | -0.02 | 0.01 | **0.80** |
| RR_1R | Lot of responsibility at home | -0.05 | 0.01 | 0.02 | 0.00 | 0.00 | **0.83** |
| RR_5R | Parent/s look to you for help as a child | 0.28 | 0.19 | -0.13 | 0.05 | 0.02 | **0.56** |
| CTQ_1 | Not enough food to eat | -0.12 | 0.22 | 0.09 | 0.30 | -0.07 | **0.46** |
| CTQ_6 | Wore dirty clothes | -0.11 | 0.22 | 0.03 | 0.17 | -0.12 | **0.36** |
| Highest factor loadings for a given factor are bolded; Cross-loadings are underlined. | | | | | | | |

# **Table S6.** Factor loadings of the third exploratory factor analysis for Early Exposome items differentiating maternal and paternal figures (n = 1.181).

|  | | | | | |  | |  |
| --- | --- | --- | --- | --- | --- | --- | --- | --- |
| **Item** | **Content** | **FAC1** | **FAC2** | **FAC3** | **FAC4** | | **FAC5** | |
| ANTIPATHY_M1 | Was difficult to please | **0.63** | -0.01 | -0.13 | 0.22 | | 0.28 | |
| ANTIPATHY_M2 | Made me feel unwanted | **0.91** | -0.13 | 0.08 | -0.10 | | 0.02 | |
| ANTIPATHY_M3 | Was critical | **0.73** | -0.15 | -0.14 | 0.20 | | 0.37 | |
| ANTIPATHY_M4 | Made me feel I was a nuisance | **0.85** | 0.03 | -0.04 | 0.01 | | 0.07 | |
| ANTIPATHY_M5 | Picked on me unfairly | **0.72** | -0.09 | -0.07 | 0.15 | | 0.01 | |
| ANTIPATHY_M6 | Did not like me as much as my siblings | **0.69** | 0.08 | -0.05 | -0.09 | | -0.03 | |
| PA_M1 | Undermined my confidence | **0.61** | 0.02 | -0.05 | 0.26 | | 0.19 | |
| PA_M2 | Confuse me by telling contradictory things | **0.49** | 0.04 | -0.01 | 0.27 | | 0.09 | |
| PA_M3 | Played on my fears | **0.61** | -0.03 | -0.05 | 0.31 | | 0.14 | |
| PA_M4 | Humiliated me, put me down | **0.75** | -0.08 | -0.07 | 0.16 | | 0.15 | |
| PA_M5 | Shamed me in front of others | **0.52** | -0.05 | 0.02 | 0.22 | | 0.14 | |
| PA_M6 | Was rejecting | **0.82** | -0.01 | -0.03 | -0.04 | | 0.02 | |
| PA_M7 | Deprivation of light, food, or company | **0.48** | 0.02 | 0.05 | 0.09 | | 0.05 | |
| PA_M8 | Made me feel guilty, so I would do what I was told | **0.59** | -0.04 | -0.01 | 0.30 | | 0.09 | |
| PA_M9 | Threatened to hurt loved ones to get what she wanted | **0.56** | -0.06 | -0.14 | 0.16 | | -0.21 | |
| PA_M11 | Said wanted me dead | **0.75** | -0.09 | -0.03 | -0.04 | | 0.00 | |
| CARE_M1 | Spoke in a warm and friendly voice | **-0.74** | -0.07 | -0.07 | -0.04 | | 0.12 | |
| CARE_M2 | Not helped me as much as I needed | **0.62** | 0.19 | 0.05 | -0.11 | | -0.12 | |
| CARE_M3 | Understood my problems and worries | **-0.68** | -0.12 | -0.02 | -0.13 | | 0.05 | |
| CARE_M4 | Was affectionate to me | **-0.76** | -0.10 | -0.13 | 0.13 | | 0.12 | |
| CARE_M5 | Enjoyed talking things over with me | **-0.71** | -0.16 | -0.01 | -0.06 | | 0.14 | |
| CARE_M6 | Frequently smiled at me | **-0.73** | -0.11 | -0.04 | 0.03 | | 0.04 | |
| CARE_M7 | Made me feel better when I was upset | **-0.53** | -0.16 | -0.01 | -0.10 | | 0.18 | |
| CARE_M8 | Did not talk with me very much | **0.67** | 0.17 | 0.09 | -0.08 | | -0.16 | |
| CARE_M9 | Emotionally cold to me | **0.80** | 0.02 | 0.15 | -0.12 | | -0.10 | |
| CARE_M10 | Did not understand what I needed or wanted | **0.66** | 0.17 | 0.02 | 0.17 | | -0.01 | |
| CARE_M11 | Made me feel I wasn’t wanted | **0.87** | 0.04 | 0.05 | -0.09 | | -0.05 | |
| CARE_M12 | Did not praise me | **0.69** | 0.16 | 0.07 | -0.03 | | -0.04 | |
| ANTIPATHY_F4 | Picked on me unfairly | 0.11 | **0.38** | 0.28 | -0.12 | | 0.25 | |
| ANTIPATHY_F5 | Was there if I needed | 0.03 | **0.40** | -0.05 | -0.13 | | 0.05 | |
| ANTIPATHY_F6 | Did not like me as much as my siblings | 0.18 | **0.50** | 0.16 | -0.11 | | -0.03 | |
| PA_P6 | Was rejecting | 0.13 | **0.49** | 0.02 | 0.00 | | 0.47 | |
| CARE_P1 | Spoke in a warm and friendly voice | 0.07 | **-0.81** | -0.13 | 0.01 | | 0.00 | |
| CARE_P2 | Did not help me as much as I needed | 0.14 | **0.62** | -0.08 | 0.04 | | 0.03 | |
| CARE_P3 | Understood my problems and worries | -0.04 | **-0.72** | -0.06 | -0.05 | | -0.07 | |
| CARE_P4 | Was affectionate to me | -0.01 | **-0.87** | 0.10 | -0.05 | | 0.01 | |
| CARE_P5 | Enjoyed talking things over with me | 0.04 | **-0.75** | 0.02 | -0.04 | | -0.20 | |
| CARE_P6 | Frequently smiled at me | 0.00 | **-0.83** | -0.06 | -0.05 | | 0.04 | |
| CARE_P7 | Made me feel better when I was upset | -0.03 | **-0.73** | -0.01 | 0.02 | | 0.00 | |
| CARE_P8 | Did not talk with me very much | -0.01 | **0.88** | -0.10 | 0.12 | | -0.04 | |
| CARE_P9 | Emotionally cold to me | 0.01 | **0.85** | -0.05 | 0.10 | | 0.01 | |
| CARE_P10 | Did not understand what I needed or wanted | 0.12 | **0.58** | 0.20 | 0.00 | | 0.16 | |
| CARE_P11 | Made me feel I wasn’t wanted | 0.09 | **0.66** | 0.13 | -0.12 | | 0.11 | |
| CARE_P12 | Did not praise me | 0.05 | **0.77** | 0.00 | 0.01 | | 0.07 | |
| PA_P2 | Played on my fears | 0.13 | 0.31 | **0.37** | -0.06 | | 0.24 | |
| PBI_OVERPR_P1X | Let me do things I liked doing | 0.07 | -0.31 | **-0.57** | -0.10 | | 0.07 | |
| PBI_OVERPR_P2X | Liked me to make my own decisions | -0.01 | -0.26 | **-0.63** | -0.04 | | 0.03 | |
| PBI_OVERPR_P7X | Did not want me to grow up | 0.09 | -0.06 | **0.49** | 0.05 | | -0.05 | |
| PBI_OVERPR_P8X | Tried to control everything I did | -0.06 | 0.00 | **0.79** | 0.07 | | 0.03 | |
| PBI_OVERPR_P9X | Invaded my privacy | 0.12 | -0.14 | **0.76** | 0.00 | | 0.09 | |
| PBI_OVERPR_P10X | Tended to baby me | 0.00 | 0.05 | **0.56** | 0.19 | | -0.05 | |
| PBI_OVERPR_P3X | Let me decide things for myself | 0.01 | -0.19 | **-0.60** | -0.10 | | -0.07 | |
| PBI_OVERPR_P11X | Tried to make me feel dependent on him | 0.03 | 0.04 | **0.69** | 0.02 | | 0.05 | |
| PBI_OVERPR_P12X | Felt I could not look after myself unless he was around | 0.10 | -0.06 | **0.60** | 0.10 | | -0.07 | |
| PBI_OVERPR_P4X | Gave me as much freedom as I wanted | 0.18 | 0.30 | **-0.38** | 0.02 | | -0.19 | |
| PBI_OVERPR_P5X | Let me go out as often as I wanted | 0.11 | 0.08 | **-0.62** | -0.16 | | -0.01 | |
| PBI_OVERPR_P13X | Was overprotective of me | -0.09 | -0.22 | **0.71** | 0.16 | | -0.04 | |
| PBI_OVERPR_P6X | Let me dress in any way I pleased | -0.08 | -0.06 | **-0.56** | -0.12 | | 0.07 | |
| PBI_OVERPR_M1X | Let me do things I liked doing | -0.21 | -0.04 | -0.32 | **-0.34** | | 0.09 | |
| PBI_OVERPR_M2X | Liked me to make my own decisions | -0.32 | -0.12 | -0.11 | **-0.52** | | 0.01 | |
| PBI_OVERPR_M7X | Did not want me to grow up | 0.00 | 0.18 | 0.06 | **0.47** | | -0.07 | |
| PBI_OVERPR_M8X | Tried to control everything I did | 0.16 | 0.01 | 0.07 | **0.72** | | 0.02 | |
| PBI_OVERPR_M9X | Invaded my privacy | 0.28 | 0.00 | 0.11 | **0.61** | | 0.08 | |
| PBI_OVERPR_M10X | Tended to baby me | 0.02 | 0.10 | 0.07 | **0.63** | | -0.06 | |
| PBI_OVERPR_M3X | Let me decide things for myself | -0.18 | -0.06 | -0.16 | **-0.58** | | -0.02 | |
| PBI_OVERPR_M11X | Tried to make me feel dependent on her | 0.22 | 0.23 | 0.07 | **0.60** | | -0.17 | |
| PBI_OVERPR_M12X | Felt I could not look after myself unless she was around | -0.03 | 0.22 | 0.11 | **0.52** | | -0.08 | |
| PBI_OVERPR_M4X | Gave me as much freedom as I wanted | 0.24 | 0.17 | -0.02 | **-0.43** | | -0.18 | |
| PBI_OVERPR_M5X | Let me go out as often as I wanted | 0.09 | 0.09 | -0.27 | **-0.48** | | -0.01 | |
| PBI_OVERPR_M13X | Was overprotective of me | -0.20 | 0.05 | 0.12 | **0.80** | | 0.01 | |
| PBI_OVERPR_M6X | Let me dress in any way I pleased | -0.21 | 0.03 | -0.24 | **-0.43** | | 0.02 | |
| ANTIPATHY_F1 | Was difficult to please | 0.07 | 0.43 | 0.23 | -0.06 | | **0.43** | |
| ANTIPATHY_F2 | Was critical of me | 0.11 | 0.39 | 0.25 | -0.11 | | **0.53** | |
| ANTIPATHY_F3 | Made me feel I was a nuisance | 0.20 | 0.45 | 0.10 | -0.18 | | **0.47** | |
| PA_P1 | Undermined my confidence | 0.05 | 0.31 | 0.24 | -0.05 | | **0.51** | |
| PA_P3 | Liked to see me suffer | 0.03 | 0.27 | 0.21 | -0.02 | | **0.50** | |
| PA_P4 | Humiliated me, put me down. | 0.11 | 0.32 | 0.22 | -0.07 | | **0.57** | |
| PA_P5 | Shamed me in front of others | 0.10 | 0.16 | 0.18 | 0.06 | | **0.50** | |
| PA_P7 | Took away things I cherished | 0.09 | 0.12 | 0.28 | 0.10 | | **0.54** | |
| PA_P8 | Made me feel guilty, so I would do what I was told | 0.12 | 0.09 | 0.38 | -0.05 | | **0.51** | |
| PA_M10 | Forced me to steal or break the law | 0.21 | -0.01 | 0.21 | -0.05 | | **-0.89** | |
|  |  |  |  |  |  | |  | |
| Highest factor loadings for a given factor are bolded; Cross-loadings are underlined. | | | | | |  | |  |

#

# **Table S7.** Factor loadings of the fourth exploratory factor analysis of all remaining Early Exposome items (n = 1.181).

|  | | | | | |
| --- | --- | --- | --- | --- | --- |
| **Item** | **Content** | **FAC1** | **FAC2** | **FAC3** | **FAC4** |
| Support1R | Adults could go to with problems/discuss feelings | **-0.33** | -0.23 | 0.33 | 0.01 |
| PBI_Mot_OVERPR | Subscale score | **0.56** | 0.17 | 0.15 | -0.08 |
| CTQ_3 | Family called me things like “stupid”, “lazy” or “ugly” | **0.35** | 0.30 | -0.06 | 0.22 |
| ANTIPATHY_M1 | Was difficult to please | **0.81** | 0.04 | 0.05 | 0.00 |
| ANTIPATHY_M2 | Made me feel unwanted | **0.80** | -0.12 | -0.14 | 0.04 |
| ANTIPATHY_M3 | Was critical | **0.90** | -0.02 | 0.07 | 0.02 |
| ANTIPATHY_M4 | Made me feel I was a nuisance | **0.80** | 0.05 | 0.03 | 0.13 |
| ANTIPATHY_M5 | Picked on me unfairly | **0.74** | -0.10 | 0.09 | 0.20 |
| ANTIPATHY_M6 | Did not like me as much as my siblings | **0.61** | -0.02 | -0.10 | 0.03 |
| PA_M1 | Undermined my confidence | **0.77** | 0.13 | 0.12 | -0.04 |
| PA_M2 | Confuse me by telling contradictory things | **0.60** | 0.09 | 0.08 | 0.08 |
| PA_M3 | Played on my fears | **0.71** | 0.06 | 0.09 | 0.08 |
| PA_M4 | Humiliated me, put me down | **0.78** | 0.00 | 0.10 | 0.15 |
| PA_M5 | Shamed me in front of others | **0.59** | 0.05 | 0.03 | 0.07 |
| PA_M6 | Was rejecting | **0.78** | -0.04 | -0.08 | 0.00 |
| PA_M7 | Deprivation of light, food, or company | **0.41** | 0.09 | 0.07 | 0.35 |
| PA_M8 | Made me feel guilty, so I would do what I was told | **0.71** | 0.05 | 0.12 | 0.08 |
| PA_M9 | Threatened to hurt loved ones to get what she wanted | **0.56** | -0.31 | 0.03 | 0.35 |
| PA_M11 | Said wanted me dead | **0.55** | -0.17 | -0.14 | 0.36 |
| CARE_M1 | Spoke in a warm and friendly voice | **-0.78** | 0.01 | 0.03 | 0.04 |
| CARE_M2 | Not helped me as much as I needed | **0.50** | 0.08 | -0.12 | 0.09 |
| CARE_M3 | Understood my problems and worries | **-0.76** | -0.03 | 0.10 | 0.08 |
| CARE_M4 | Was affectionate to me | **-0.73** | -0.04 | 0.05 | 0.05 |
| CARE_M5 | Enjoyed talking things over with me | **-0.80** | -0.02 | 0.06 | 0.16 |
| CARE_M6 | Frequently smiled at me | **-0.72** | -0.03 | 0.10 | 0.05 |
| CARE_M7 | Made me feel better when I was upset | **-0.63** | 0.01 | 0.07 | 0.12 |
| CARE_M8 | Did not talk with me very much | **0.67** | 0.03 | -0.12 | -0.11 |
| CARE_M9 | Emotionally cold to me | **0.76** | 0.00 | -0.03 | -0.03 |
| CARE_M10 | Did not understand what I needed or wanted | **0.74** | 0.11 | -0.07 | -0.07 |
| CARE_M11 | Made me feel I wasn’t wanted | **0.79** | -0.01 | -0.12 | -0.01 |
| CARE_M12 | Did not praise me | **0.70** | 0.11 | -0.10 | -0.13 |
| PBI_Fat_OVERPR | Subscale score | 0.05 | **0.53** | 0.08 | 0.02 |
| CTQ_19 | Felt that someone in my family hated me | 0.14 | **0.29** | -0.17 | 0.26 |
| ANTIPATHY_F1 | Was difficult to please | -0.01 | **0.82** | -0.02 | 0.02 |
| ANTIPATHY_F2 | Was critical of me | -0.02 | **0.86** | -0.06 | -0.01 |
| ANTIPATHY_F3 | Made me feel I was a nuisance | 0.01 | **0.80** | -0.02 | 0.11 |
| ANTIPATHY_F4 | Picked on me unfairly | -0.07 | **0.75** | 0.00 | 0.14 |
| ANTIPATHY_F5 | Was there if I needed | -0.07 | **0.36** | -0.13 | -0.06 |
| ANTIPATHY_F6 | Did not like me as much as my siblings | 0.11 | **0.52** | -0.12 | -0.11 |
| PA_P1 | Undermined my confidence | -0.05 | **0.78** | 0.02 | 0.07 |
| PA_P2 | Played on my fears | 0.00 | **0.75** | 0.07 | 0.14 |
| PA_P3 | Liked to see me suffer | -0.09 | **0.70** | -0.05 | 0.11 |
| PA_P4 | Humiliated me, put me down. | -0.03 | **0.84** | 0.07 | 0.14 |
| PA_P5 | Shamed me in front of others | 0.04 | **0.59** | 0.02 | 0.09 |
| PA_P6 | Was rejecting | 0.05 | **0.80** | 0.04 | 0.09 |
| PA_P7 | Took away things I cherished | 0.02 | **0.62** | -0.01 | 0.15 |
| PA_P8 | Made me feel guilty, so I would do what I was told | 0.01 | **0.70** | 0.18 | 0.26 |
| CARE_P1 | Spoke in a warm and friendly voice | 0.09 | **-0.86** | 0.02 | 0.03 |
| CARE_P2 | Did not help me as much as I needed | 0.12 | **0.51** | -0.12 | -0.02 |
| CARE_P3 | Understood my problems and worries | -0.05 | **-0.72** | 0.11 | 0.09 |
| CARE_P4 | Was affectionate to me | -0.07 | **-0.73** | 0.02 | 0.10 |
| CARE_P5 | Enjoyed talking things over with me | -0.03 | **-0.82** | 0.04 | 0.18 |
| CARE_P6 | Frequently smiled at me | -0.06 | **-0.79** | 0.00 | 0.12 |
| CARE_P7 | Made me feel better when I was upset | -0.03 | **-0.68** | 0.05 | 0.08 |
| CARE_P8 | Did not talk with me very much | 0.09 | **0.73** | -0.03 | -0.14 |
| CARE_P9 | Emotionally cold to me | 0.09 | **0.76** | -0.03 | -0.13 |
| CARE_P10 | Did not understand what I needed or wanted | 0.07 | **0.73** | -0.10 | -0.01 |
| CARE_P11 | Made me feel I wasn’t wanted | -0.04 | **0.75** | -0.11 | 0.02 |
| CARE_P12 | Did not praise me | 0.07 | **0.74** | -0.10 | -0.17 |
| Support2R | Peers to discuss problems and feelings | 0.09 | -0.02 | **0.69** | 0.04 |
| BCE1_R | Caregiver provided sense of safety | -0.10 | -0.11 | **0.42** | -0.01 |
| BCE2_R | At least one good friend | 0.06 | 0.07 | **0.68** | 0.01 |
| BCE_41R | Liked school | 0.02 | -0.10 | **0.44** | -0.10 |
| BCE_42R | Liked high school | 0.02 | 0.01 | **0.65** | 0.00 |
| BCE5_R | At least one teacher that cared | 0.12 | -0.09 | **0.51** | 0.01 |
| BCE7_R | Adult who provided support or advice | -0.07 | -0.08 | **0.52** | 0.03 |
| PCE1_R | Capable of discussing feelings with family | -0.27 | -0.14 | **0.49** | 0.10 |
| PCE2_R | Supported by family during tough times | -0.34 | -0.13 | **0.48** | -0.01 |
| PCE3_R | Enjoyed community traditions | -0.08 | -0.12 | **0.60** | -0.03 |
| PCE4_R | Sense of belonging in high school | -0.05 | 0.07 | **0.74** | 0.03 |
| PCE5_R | Felt supported by friends | 0.05 | 0.02 | **0.70** | 0.01 |
| PCE6_R | At least two caring adults (not parents) | -0.10 | -0.15 | **0.46** | -0.06 |
| PCE7_R | Felt safe and protected by an adult at home | -0.22 | -0.15 | **0.50** | -0.26 |
| BCE_8 | Opportunities to have a good time | 0.09 | -0.04 | **0.71** | -0.11 |
| BCE_9 | Like or felt comfortable with oneself | -0.06 | -0.08 | **0.49** | -0.08 |
| CTQ_2 | Family member took care/protected me | -0.15 | -0.10 | **0.44** | -0.25 |
| CTQ_5 | Family member made feel important | -0.35 | -0.14 | **0.41** | 0.06 |
| CTQ_7 | Felt loved | -0.30 | -0.27 | **0.44** | -0.13 |
| CTQ_13 | People in family looked out for each other | -0.18 | -0.25 | **0.29** | -0.28 |
| CTQ_20 | Family felt close | -0.14 | -0.27 | **0.32** | -0.24 |
| CTQ_27 | Someone take to doctor if I needed it | -0.12 | -0.06 | **0.26** | -0.19 |
| CTQ_29 | Family was source of strength | -0.31 | -0.29 | **0.34** | -0.18 |
| FAMILYPROT2 | Ways to have fun at home | -0.15 | -0.26 | **0.37** | -0.28 |
| BCE10_R | Had a predictable home routine | 0.00 | -0.04 | 0.31 | **-0.47** |
| CTQ_Physical_Abuse | Subscale score | 0.19 | 0.17 | 0.00 | **0.33** |
| CTQ_Sexual_Abuse | Subscale score | 0.04 | -0.09 | -0.15 | **0.29** |
| CECA_Discord | Subscale score | 0.13 | 0.23 | -0.02 | **0.50** |
| CTQ_1 | Not enough food to eat | 0.05 | -0.06 | -0.16 | **0.61** |
| CTQ_4 | Parents too drunk or to high | -0.14 | 0.13 | -0.11 | **0.55** |
| CTQ_6 | Wore dirty clothes | -0.02 | -0.12 | -0.20 | **0.53** |
| CTQ_14 | Family said hurful or insulting things | 0.19 | 0.34 | -0.03 | **0.37** |
| CTQ_26 | Was emotionally abused | 0.24 | 0.30 | -0.11 | **0.39** |
| RR_1R | Lot of responsibility at home | 0.07 | 0.03 | -0.05 | **0.57** |
| RR_2R | Expected to do a lot of housework | 0.13 | 0.01 | -0.07 | **0.49** |
| RR_3R | Had to look after younger siblings | 0.13 | 0.02 | 0.00 | **0.42** |
| RR_4R | Missed out seeing friends because of home responsibilities | 0.14 | 0.08 | -0.02 | **0.46** |
| RR_5R | Parent/s look to you for help as a child | 0.02 | -0.07 | 0.01 | **0.59** |
| RR_6R | Parent/s confided their problems in you | -0.07 | -0.06 | 0.08 | **0.57** |
| RR_8R | Parent/s rely for emotional support as a child | 0.14 | 0.15 | 0.03 | **0.47** |
| RR_9R | Felt concerned and worried about parents | -0.06 | 0.08 | -0.01 | **0.56** |
| FAMILYPROT3 | Lived in a happy home | -0.13 | -0.34 | 0.28 | **-0.39** |
| FAMILYPROT4 | Home overly chaotic or noisy | -0.10 | -0.14 | 0.06 | **-0.39** |
| CECA_Violence | Subscale score | -0.08 | 0.10 | 0.03 | **0.44** |
|  |  |  |  |  |  |

Highest factor loadings for a given factor are bolded; Cross-loadings are underlined.

#

# **Figure S1.** Scree plot of eigenvalues of the mixed correlation matrix of the final 99 early environmental items


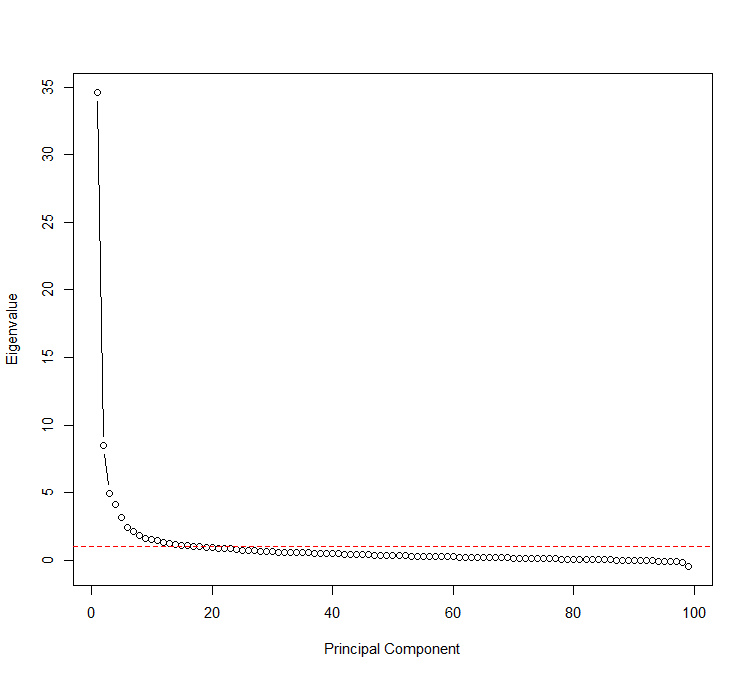


One method to determine the optimal number of factors to retain in analysis is to visually inspect the scree plot of eigenvalues and identify the 'elbow' point, which marks where the eigenvalues transition from a steep decline to a more gradual, linear decrease. The exact location of the elbow is often unclear, requiring the researcher to identify breaks that signal significant drops between eigenvalues. In this case, a break is observed between the 5^th^ and 6^th^ eigenvalues, preceding a clear linear trend, suggesting the retention of 4 or 5 factors. Parallel analysis suggested a five-factor structure; however, examination of the fifth factor revealed that it contained only four items, all exhibiting high cross-loadings and one of the items did not meet the threshold of .30. Since the goal of the analysis is to achieve a conceptually sound and interpretable solution, while ensuring robust data interpretation, four factors were retained. This decision strikes a balance between theoretical relevance and practical application.

# **Table S8.** Factor loadings of the confirmatory factor analysis of the optimized collection of Early Exposome items (n = 1.181).

|  | | | | | | | |  |
| --- | --- | --- | --- | --- | --- | --- | --- | --- |
| **Item** | **Content** | **Early Exposome** | **FAC1**  **Positive**  **Experiences** | **FAC2**  **Paternal**  **Aversity** | **FAC3**  **Maternal**  **Adversity** | **FAC4**  **Role Reversal** | | |
| PCE4_R | Sense of belonging in high school | -0.46 | 0.72 |  |  | |  | |
| BCE_8 | Opportunities to have a good time | -0.54 | 0.56 |  | 0.08 | |  | |
| PCE5_R | Felt supported by friends | -0.40 | 0.66 |  |  | |  | |
| Support2R | Peers to discuss problems and feelings | -0.40 | 0.52 |  |  | |  | |
| BCE2_R | At least one good friend | -0.33 | 0.68 |  |  | |  | |
| BCE_42R | Liked high school | -0.41 | 0.66 |  |  | |  | |
| PCE3_R | Enjoyed community traditions | -0.61 | 0.38 |  |  | |  | |
| BCE7_R | Adult who provided support or advice | -0.49 | 0.29 |  |  | |  | |
| PCE7_R | Felt safe and protected by an adult at home | -0.85 | 0.10 |  |  | |  | |
| PCE1_R | Capable of discussing feelings with family | -0.70 | 0.16 |  |  | |  | |
| PCE2_R | Supported by family during tough times | -0.79 | 0.13 |  |  | |  | |
| BCE5_R | At least one teacher that cared | -0.35 | 0.34 |  |  | |  | |
| BCE_9 | Like or felt comfortable with oneself | -0.51 | 0.39 |  |  | |  | |
| CTQ_7 | Felt loved | -0.90 | 0.08 |  |  | |  | |
| PCE6_R | At least two caring adults (not parents) | -0.58 | 0.23 |  |  | |  | |
| BCE_41R | Liked school | -0.42 | 0.37 |  |  | |  | |
| CTQ_5 | Family member made me feel important | -0.73 | 0.07 |  |  | |  | |
| CTQ_2 | Family member took care of me | -0.70 | 0.07 |  |  | |  | |
| BCE1_R | Caregiver provided sense of safety | -0.49 | 0.18 |  |  | |  | |
| FAMILYPROT2 | Ways to have fun at home | -0.80 | 0.01 |  |  | |  | |
| CTQ_29 | Family was source of strength | -0.89 | -0.05 |  |  | |  | |
| Support1R | Adults could go to with problems/discuss feelings | -0.76 | 0.05 |  |  | |  | |
| CTQ_20 | Family felt close | -0.73 | 0.01 |  |  | |  | |
| FAMILYPROT3 | Lived in a happy home | -0.83 | -0.05 |  |  | | -0.13 | |
| CARE_P1 | Spoke in a warm and friendly voice | -0.52 |  | -0.67 |  | |  | |
| PA_P4 | Humiliated me, put me down. | 0.61 |  | 0.58 |  | |  | |
| ANT_FATHER2 | Was critical of me | 0.62 |  | 0.61 |  | |  | |
| ANT_FATHER1 | Was difficult to please | 0.59 |  | 0.56 |  | |  | |
| PA_P6 | Was rejecting | 0.63 |  | 0.53 |  | |  | |
| ANT_FATHER3 | Made me feel I was a nuisance | 0.65 |  | 0.54 |  | |  | |
| CARE_P5 | Enjoyed talking things over with me | -0.52 |  | -0.70 |  | |  | |
| CARE_P6 | Frequently smiled at me | -0.54 |  | -0.66 |  | |  | |
| PA_P1 | Undermined my confidence | 0.53 |  | 0.53 |  | |  | |
| PA_P2 | Played on my fears | 0.57 |  | 0.47 |  | |  | |
| ANT_FATHER4 | Picked on me unfairly | 0.54 |  | 0.50 |  | |  | |
| CARE_P9 | Emotionally cold to me | 0.56 |  | 0.62 |  | |  | |
| PA_P8 | Made me feel guilty, so I would do what I was told | 0.52 |  | 0.42 |  | |  | |
| CARE_P11 | Made me feel I wasn’t wanted | 0.59 |  | 0.52 |  | |  | |
| CARE_P4 | Was affectionate to me | -0.48 |  | -0.52 |  | |  | |
| CARE_P12 | Did not praise me | 0.57 |  | 0.58 |  | |  | |
| CARE_P8 | Did not talk with me very much | 0.53 |  | 0.64 |  | |  | |
| CARE_P10 | Did not understand what I needed or wanted | 0.66 |  | 0.49 |  | |  | |
| CARE_P3 | Understood my problems and worries | -0.60 |  | -0.55 |  | |  | |
| PA_P3 | Liked to see me suffer | 0.58 |  | 0.55 |  | |  | |
| CARE_P7 | Made me feel better when I was upset | -0.52 |  | -0.52 |  | |  | |
| PA_P7 | Took away things I cherished | 0.55 |  | 0.38 |  | |  | |
| PA_P5 | Shamed me in front of others | 0.49 |  | 0.38 |  | |  | |
| PBI_Fat_OVERPR | Subscale score | 0.37 |  | 0.25 |  | |  | |
| ANT_FATHER6 | Did not like me as much as my siblings | 0.51 |  | 0.36 |  | |  | |
| CARE_P2 | Did not help me as much as I needed | 0.56 |  | 0.37 |  | |  | |
| ANT_FATHER5 | Was there if I needed | 0.25 |  | 0.28 |  | |  | |
| ANT_MOTHER3 | Was critical | 0.56 |  |  | 0.65 | |  | |
| ANT_MOTHER1 | Was difficult to please | 0.57 |  |  | 0.57 | |  | |
| ANT_MOTHER_4 | Made me feel I was a nuisance | 0.66 |  |  | 0.55 | |  | |
| PA_M4 | Humiliated me, put me down | 0.54 |  |  | 0.57 | |  | |
| PA_M1 | Undermined my confidence | 0.54 |  |  | 0.52 | |  | |
| ANT_MOTHER5 | Picked on me unfairly | 0.46 |  |  | 0.56 | |  | |
| CARE_M5 | Enjoyed talking things over with me | -0.57 |  |  | -0.57 | |  | |
| CARE_M1 | Spoke in a warm and friendly voice | -0.56 |  |  | -0.57 | |  | |
| PA_M8 | Made me feel guilty, so I would do what I was told | 0.49 |  |  | 0.52 | |  | |
| CARE_M9 | Emotionally cold to me | 0.55 |  |  | 0.60 | |  | |
| PA_M3 | Played on my fears | 0.52 |  |  | 0.51 | |  | |
| CARE_M11 | Made me feel I wasn’t wanted | 0.65 |  |  | 0.54 | |  | |
| ANT_MOTHER2 | Made me feel unwanted | 0.61 |  |  | 0.62 | |  | |
| PA_M6 | Was rejecting | 0.58 |  |  | 0.53 | |  | |
| CARE_M4 | Was affectionate to me | -0.59 |  |  | -0.59 | |  | |
| CARE_M3 | Understood my problems and worries | -0.63 |  |  | -0.50 | |  | |
| CARE_M10 | Did not understand what I needed or wanted | 0.66 |  |  | 0.49 | |  | |
| CARE_M6 | Frequently smiled at me | -0.59 |  |  | -0.56 | |  | |
| CARE_M12 | Did not praise me | 0.61 |  |  | 0.48 | |  | |
| PA_M2 | Confuse me by telling contradictory things | 0.48 |  |  | 0.40 | |  | |
| PBI_Mot_OVERPR | Subscale score | 0.39 |  |  | 0.32 | |  | |
| CARE_M8 | Did not talk with me very much | 0.56 |  |  | 0.50 | |  | |
| PA_M5 | Shamed me in front of others | 0.48 |  |  | 0.39 | |  | |
| CARE_M7 | Made me feel better when I was upset | -0.46 |  |  | -0.43 | |  | |
| PA_M9 | Threatened to hurt loved ones to get what she wanted | 0.14 |  |  | 0.72 | | 0.44 | |
| ANT_MOTHER6 | Did not like me as much as my siblings | 0.51 |  |  | 0.41 | |  | |
| PA_M11 | Said wanted me dead | 0.50 |  |  | 0.49 | | 0.28 | |
| CARE_M2 | Not helped me as much as I needed | 0.58 |  |  | 0.32 | |  | |
| PA_M7 | Deprivation of light, food, or company | 0.46 |  |  | 0.31 | | 0.23 | |
| CTQ_3 | Family called me things like “stupid”, “lazy” or “ugly” | 0.71 |  |  | 0.10 | |  | |
| RR_5R | Parent/s look to you for help as a child | 0.20 |  |  |  | | 0.64 | |
| RR_1R | Lot of responsibility at home | 0.36 |  |  |  | | 0.85 | |
| CTQ_1 | Not enough food to eat | 0.39 |  |  |  | | 0.49 | |
| RR_6R | Parent/s confided their problems in you | 0.06 |  |  |  | | 0.63 | |
| RR_9R | Felt concerned and worried about parents | 0.26 |  |  |  | | 0.49 | |
| RR_2R | Expected to do a lot of housework | 0.39 |  |  |  | | 0.82 | |
| CECA_Discord | Subscale score | 0.53 |  |  |  | | 0.26 | |
| CTQ_4 | Parents too drunk or to high | 0.36 |  |  |  | | 0.30 | |
| CTQ_6 | Wore dirty clothes | 0.27 |  |  |  | | 0.41 | |
| RR_8R | Parent/s rely for emotional support as a child | 0.43 |  |  |  | | 0.47 | |
| RR_4R | Missed out seeing friends because of home responsibilities | 0.40 |  |  |  | | 0.57 | |
| BCE10_R | Had a predictable home routine | -0.48 |  |  |  | | -0.31 | |
| RR_3R | Had to look after younger siblings | 0.30 |  |  |  | | 0.48 | |
| CECA_Violence | Subscale score | 0.19 |  |  |  | | 0.24 | |
| FAMILYPROT4 | Home overly chaotic or noisy | -0.43 |  |  |  | | -0.26 | |
| CTQ_26 | Was emotionally abused | 0.74 |  |  |  | | 0.12 | |
| CTQ_14 | Family said hurful or insulting things | 0.68 |  |  |  | | 0.11 | |
| CTQ_Phys_Abuse | Subscale score | 0.40 |  |  |  | | 0.08 | |
|  |  |  |  |  |  | |  | |

|  | Explained Common Variance  (ECV SS) | Explained Common Variance  (ECV SG) | Explained Common Variance  (ECV GS) | Omega  ω | Omega-Hierarchical (Omega H) | H | Factor Determinacy (FD) |
| --- | --- | --- | --- | --- | --- | --- | --- |
| Early Exposome Factor | 0.57 | 0.57 | 0.57 | 0.86 | 0.20 | 0.98 | 0.98 |
| F1 Positive Experiences | 0.25 | 0.06 | 0.75 | 0.96 | 0.16 | 0.84 | 0.93 |
| F2 Paternal Adversity | 0.48 | 0.14 | 0.52 | 0.91 | 0.35 | 0.92 | 0.96 |
| F3 Maternal Adversity | 0.44 | 0.15 | 0.52 | 0.92 | 0.44 | 0.92 | 0.96 |
| F4 Role Reversal | 0.46 | 0.08 | 0.45 | 0.87 | 0.55 | 0.90 | 0.96 |

# **Table S9.** Bifactor indices for the Early Exposome model

ECV: Explained Common Variance; ECV_SS: ECV of a specific factor relative to itself; ECV_SG: ECV of a specific factor relative to the general factor; ECV_GS: ECV of the general factor relative to a specific factor.

# **Table S10.** Factor loadings of the exploratory factor analysis of the collection of P-factor items using iterative target rotation (*n* = 1.181).

|  | | | | | | | | | | |  |
| --- | --- | --- | --- | --- | --- | --- | --- | --- | --- | --- | --- |
| **Item** | **Content** | | **FAC1**  **Affective Dysregulation** | **FAC2**  **Social/Cognitive Malfunctioning** | | **FAC3**  **Positive Schizotypy** | **FAC4**  **Negative**  **Schizotypy** | | |  |  |
| BAI_11 | Feeling of choking | | **0.83** | -0.14 | | 0.03 | -0.04 | | |  |  |
| BAI_15 | Difficulty in breathing | | **0.79** | -0.11 | | 0.00 | -0.11 | | |  |  |
| BAI_13 | Shaky / unsteady | | **0.75** | 0.01 | | 0.00 | -0.13 | | |  |  |
| BAI_19 | Faint / lightheaded | | **0.71** | -0.14 | | 0.06 | -0.11 | | |  |  |
| BAI_9 | Terrified or afraid | | **0.70** | -0.01 | | 0.11 | 0.00 | | |  |  |
| BAI_7 | Heart pounding / racing | | **0.69** | -0.04 | | 0.07 | -0.09 | | |  |  |
| BAI_4 | Unable to relax | | **0.66** | 0.08 | | -0.01 | -0.04 | | |  |  |
| BAI_10 | Nervous | | **0.66** | 0.06 | | 0.06 | -0.13 | | |  |  |
| BAI_8 | Unsteady | | **0.65** | 0.03 | | 0.09 | -0.05 | | |  |  |
| BAI_17 | Scared | | **0.62** | -0.03 | | 0.12 | -0.05 | | |  |  |
| BDI_1 | Sadness | | **0.62** | 0.21 | | -0.14 | 0.00 | | |  |  |
| BAI_12 | Hands trembling | | **0.62** | -0.02 | | 0.05 | -0.08 | | |  |  |
| BAI_14 | Fear of losing control | | **0.62** | 0.06 | | 0.13 | -0.01 | | |  |  |
| BAI_6 | Dizzy or lightheaded | | **0.61** | -0.05 | | 0.10 | -0.08 | | |  |  |
| BDI_20 | Tiredness or Fatigue | | **0.60** | 0.11 | | -0.04 | 0.04 | | |  |  |
| BAI_5 | Fear of worst happening | | **0.57** | 0.17 | | 0.04 | -0.17 | | |  |  |
| BDI_15 | Loss of Energy | | **0.56** | 0.20 | | -0.13 | 0.09 | | |  |  |
| BAI_3 | Wobbliness in legs | | **0.53** | 0.04 | | 0.08 | -0.01 | | |  |  |
| BDI_18 | Changes in Appetite | | **0.52** | 0.05 | | 0.01 | 0.01 | | |  |  |
| BDI_11 | Agitation | | **0.52** | 0.00 | | 0.03 | -0.04 | | |  |  |
| BDI_10 | Crying | | **0.51** | 0.15 | | -0.07 | -0.09 | | |  |  |
| BDI_17 | Irritability | | **0.50** | 0.17 | | -0.06 | 0.00 | | |  |  |
| BAI_1 | Numbness or tingling | | **0.50** | -0.09 | | 0.12 | 0.09 | | |  |  |
| BDI_4 | Loss of pleasure | | **0.49** | 0.15 | | -0.12 | 0.20 | | |  |  |
| BDI_9 | Suicidal Thoughts | | **0.49** | 0.29 | | -0.04 | 0.15 | | |  |  |
| BDI_6 | Punishment Feeling | | **0.48** | 0.13 | | 0.09 | 0.05 | | |  |  |
| BDI_21 | Loss of Interest in Sex | | **0.47** | -0.03 | | -0.09 | 0.18 | | |  |  |
| BAI_18 | Indigestion | | **0.47** | -0.04 | | 0.03 | -0.13 | | |  |  |
| BDI_19 | Concentration Difficulties | | **0.46** | 0.26 | | -0.05 | 0.03 | | |  |  |
| BDI_12 | Loss of Interest | | **0.44** | 0.20 | | -0.13 | 0.20 | | |  |  |
| BDI_7 | Self-Dislike | | **0.44** | 0.38 | | -0.22 | -0.01 | | |  |  |
| BDI_5 | Guilty Feeling | | **0.43** | 0.31 | | -0.05 | -0.02 | | |  |  |
| BDI_14 | Worthlessness | | **0.43** | 0.40 | | -0.08 | 0.07 | | |  |  |
| BDI_2 | Pessimism | | **0.41** | 0.26 | | -0.11 | 0.12 | | |  |  |
| BAI_21 | Hot / cold sweats | | **0.39** | 0.04 | | 0.08 | 0.02 | | |  |  |
| BDI_16 | Changes in Sleep | | **0.38** | 0.10 | | 0.03 | 0.10 | | |  |  |
| BDI_8 | Self-Criticalness | | **0.38** | 0.26 | | -0.02 | -0.02 | | |  |  |
| BAI_16 | Fear of dying | | **0.38** | 0.05 | | 0.08 | -0.03 | | |  |  |
| BDI_13 | Indecisiveness | | **0.36** | 0.30 | | -0.03 | -0.01 | | |  |  |
| BAI_20 | Face flushed | | **0.35** | -0.01 | | 0.16 | -0.08 | | |  |  |
| BAI_2 | Feeling hot | | **0.24** | 0.01 | | 0.19 | 0.08 | | |  |  |
| SPQ_16 | When people talking to each other, wonder if they are talking about you | | -0.09 | **0.75** | | 0.11 | -0.14 | | |  |  |
| SPQ_19 | Feel that people are talking about you | | -0.10 | **0.72** | | 0.17 | -0.19 | | |  |  |
| SPQ_7 | Others have it in for me | | 0.06 | **0.69** | | 0.03 | -0.02 | | |  |  |
| MSS2_9D | Difficult to get my thoughts in order | | 0.16 | **0.68** | | 0.00 | -0.01 | | |  |  |
| MSS2_11D | Can not organize thoughts | | 0.17 | **0.67** | | -0.05 | 0.05 | | |  |  |
| SPQ_3 | Other people have got it in for you | | 0.03 | **0.65** | | 0.06 | 0.00 | | |  |  |
| SPQ_1 | I am sure I am being talked about behind my back | | -0.13 | **0.65** | | 0.22 | -0.17 | | |  |  |
| MSS2_13D | Thoughts are almost always hard to follow | | 0.04 | **0.64** | | 0.06 | 0.06 | | |  |  |
| MSS2_17D | Thoughts and behaviors are almost always disorganized | | 0.16 | **0.63** | | -0.05 | 0.12 | | |  |  |
| MSS2_19D | Difficulty organizing what supposed to be doing | | 0.06 | **0.62** | | -0.02 | 0.02 | | |  |  |
| MSS2_12D | Thoughts hazy and unclear wish that could reach up and put them into place | | 0.18 | **0.58** | | 0.11 | 0.02 | | |  |  |
| MSS2_16D | Trouble following conversations with others | | 0.04 | **0.57** | | -0.04 | 0.25 | | |  |  |
| SPQ_17 | Do you sometimes feel that other people are watching you | | -0.12 | **0.55** | | 0.37 | -0.18 | | |  |  |
| SPQ_15 | When shopping feeling that other people are taking notice of you | | -0.16 | **0.55** | | 0.32 | 0.00 | | |  |  |
| MSS2_18D | Thoughts and behaviors feel random and unfocused | | 0.11 | **0.54** | | 0.10 | 0.18 | | |  |  |
| MSS2_20D | I often feel so mixed up that I have difficulty functioning | | 0.24 | **0.53** | | 0.08 | 0.06 | | |  |  |
| MSS2_21D | Have difficulty following what someone is saying | | 0.12 | **0.52** | | 0.02 | 0.18 | | |  |  |
| SPQ_2 | Concerned that friends or coworkers not loyal or trustworthy | | 0.02 | **0.51** | | 0.17 | -0.21 | | |  |  |
| MSS2_15D | People find my conversations confusing or hard to follow | | -0.06 | **0.50** | | 0.10 | 0.30 | | |  |  |
| SPQ_4 | I feel I have to be on my guard even with friends. | | 0.12 | **0.49** | | 0.17 | 0.09 | | |  |  |
| CAPE_5 | Conspiracy against you | | -0.03 | **0.49** | | 0.34 | 0.01 | | |  |  |
| CAPE_11 | People look at you oddly because of your appearance | | 0.04 | **0.47** | | 0.27 | 0.02 | | |  |  |
| BDI_3 | Past Failures | | 0.30 | **0.47** | | -0.09 | 0.00 | | |  |  |
| SPQ_8 | Keep an eye out to stop people from taking advantage | | 0.04 | **0.43** | | 0.29 | 0.03 | | |  |  |
| CAPE_1 | People drop hints about you or say things with a double meaning | | -0.07 | **0.40** | | 0.39 | -0.10 | | |  |  |
| SPQ_5 | Pick up hidden threats/put-downs from what people say/do | | -0.01 | **0.39** | | 0.32 | -0.05 | | |  |  |
| MSS2_14D | I find that I am very often confused about what is going on around me | | 0.18 | **0.39** | | 0.26 | 0.09 | | |  |  |
| SPQ_12 | People drop hints about you or say things with a double meaning | | -0.07 | **0.39** | | 0.38 | -0.16 | | |  |  |
| MSS_6P | Messages in the way things are arranged, like furniture | | -0.04 | -0.02 | | **0.75** | -0.05 | | |  |  |
| SPQ_13 | Noticed a common event or object to be a special sign for you | | -0.03 | -0.01 | | **0.74** | -0.11 | | |  |  |
| MSS_7P | There are secret signs in the world if you know how to look for them | | -0.04 | -0.02 | | **0.70** | -0.05 | | |  |  |
| SPQ_14 | See special meanings in advertisements, shop windows, or in the way things are arranged around | | -0.06 | 0.04 | | **0.66** | -0.08 | | |  |  |
| MSS_4P | I have sometimes felt that strangers were reading my mind. | | 0.00 | 0.04 | | **0.65** | 0.07 | | |  |  |
| CAPE_8 | Communicate telepathically | | 0.04 | -0.06 | | **0.64** | -0.02 | | |  |  |
| MSS_1P | I believe that dreams have magical properties | | 0.02 | -0.07 | | **0.64** | -0.09 | | |  |  |
| MSS_3P | Momentary feeling that someone's place has been taken by a look-alike | | 0.07 | -0.07 | | **0.64** | 0.07 | | |  |  |
| SPQ_9 | Things on the TV or newspaper have a special meaning for you | | -0.03 | 0.02 | | **0.62** | -0.01 | | |  |  |
| CAPE_12 | Thoughts in your head are being taken away from you | | 0.05 | 0.08 | | **0.60** | 0.08 | | |  |  |
| MSS_13P | Experiences with seeing the future, ESP or a sixth sense | | 0.18 | -0.12 | | **0.60** | 0.03 | | |  |  |
| CAPE_16 | Under the control of some force or power other than yourself | | 0.14 | 0.07 | | **0.59** | 0.11 | | |  |  |
| CAPE_2 | Things in magazines or on TV were written especially for you | | -0.10 | 0.07 | | **0.58** | 0.00 | | |  |  |
| MSS_14P | Worry that someone or something is controlling my behavior | | 0.04 | 0.17 | | **0.55** | 0.11 | | |  |  |
| MSS_8P | Wonder if there is a small group of people controlling everyone else's behavior | | -0.05 | 0.07 | | **0.55** | 0.12 | | |  |  |
| CAPE_6 | Destined to be someone very important | | -0.12 | 0.06 | | **0.52** | -0.03 | | |  |  |
| CAPE_10 | Believe in the power of witchcraft, voodoo or the occult | | 0.14 | -0.08 | | **0.51** | -0.08 | | |  |  |
| CAPE_7 | You are a very special or unusual person | | -0.14 | 0.03 | | **0.51** | 0.00 | | |  |  |
| CAPE_20 | See objects, people or animals that other people cannot see | | 0.16 | -0.12 | | **0.51** | 0.23 | | |  |  |
| CAPE_4 | Being persecuted in some way | | 0.05 | 0.16 | | **0.50** | 0.03 | | |  |  |
| CAPE_19 | A double has taken the place of a family member, friend or acquaintance | | -0.06 | 0.02 | | **0.50** | 0.15 | | |  |  |
| CAPE_17 | Hear voices when you are alone | | 0.22 | -0.02 | | **0.49** | 0.23 | | |  |  |
| MSS_2P | Some people make me aware of them just by thinking about me | | -0.05 | 0.07 | | **0.48** | -0.17 | | |  |  |
| CAPE_14 | Thoughts so vivid that you were worried other people would hear | | 0.13 | 0.13 | | **0.48** | 0.07 | | |  |  |
| CAPE_3 | Some people are not what they seem to be | | -0.07 | 0.31 | | **0.44** | 0.06 | | |  |  |
| MSS_12P | Feels like someone is touching me when no one is there | | 0.32 | -0.10 | | **0.44** | 0.07 | | |  |  |
| CAPE_13 | Thoughts in your head are not your own | | 0.14 | 0.23 | | **0.44** | 0.13 | | |  |  |
| MSS_11P | Wondered if my body was really my own | | 0.16 | 0.16 | | **0.43** | 0.10 | | |  |  |
| CAPE_15 | Hear your own thoughts being echoed back to you | | 0.05 | 0.22 | | **0.43** | 0.05 | | |  |  |
| CAPE_18 | Hear voices talking to each other when you are alone | | 0.28 | -0.12 | | **0.43** | 0.32 | | |  |  |
| MSS_10P | Hear people talking only to discover that there was no one there | | 0.16 | 0.09 | | **0.42** | 0.19 | | |  |  |
| CAPE_9 | Electrical devices can influence the way you think | | -0.02 | 0.05 | | **0.41** | 0.02 | | |  |  |
| SPQ_10 | People notice me when I go out for a meal or to see a film | | -0.10 | 0.38 | | **0.38** | 0.01 | | |  |  |
| MSS_9P | I often worry that other people are out to get me. | | 0.01 | 0.37 | | **0.38** | 0.00 | | |  |  |
| SPQ_6 | It is best not to let other people know too much about you | | 0.04 | 0.28 | | **0.32** | 0.15 | | |  |  |
| MSS2_2N | Spending time with close friends and family is important | | -0.08 | 0.01 | | 0.04 | **0.75** | | |  |  |
| MSS2_8N | Important to have close relationships with other people | | 0.10 | -0.02 | | 0.04 | **0.71** | | |  |  |
| MSS_17N | Do not have many thoughts or emotions | | -0.08 | 0.00 | | -0.08 | **0.65** | | |  |  |
| MSS2_6N | Not interested in being emotionally close with others | | -0.05 | 0.11 | | 0.04 | **0.61** | | |  |  |
| MSS_16N | Emotions seemed flat regardless of what is going on around | | -0.11 | 0.15 | | 0.07 | **0.61** | | |  |  |
| MSS2_1N | Having close friends is not as important as people say | | -0.03 | -0.09 | | 0.08 | **0.59** | | |  |  |
| MSS_15N | Noticed that I rarely feel strong positive or negative emotions | | -0.17 | 0.09 | | -0.02 | **0.55** | | |  |  |
| MSS2_3N | Rather be with another person than alone | | 0.03 | -0.02 | | 0.12 | **0.52** | | |  |  |
| MSS2_4N | Desire to be connected with other people | | -0.07 | -0.15 | | -0.11 | **0.51** | | |  |  |
| MSS_19N | Preferred to be disconnected from the world | | 0.04 | 0.19 | | 0.19 | **0.51** | | |  |  |
| MSS_18N | Few things have been exciting or interesting to me | | 0.02 | 0.34 | | -0.09 | **0.47** | | |  |  |
| MSS2_5N | Little interest in dating or being in a romantic relationship. | | -0.03 | 0.02 | | 0.16 | **0.35** | | |  |  |
| MSS2_7N | Not many things ever really enjoyed doing | | 0.26 | 0.32 | | -0.17 | **0.35** | | |  |  |
|  |  | |  |  | |  |  | | |  |  |
|  | | Inter-factor correlations resulting from EFA | | | | | | | | | |
|  | |  | F1 | | F2 | | | F3 | F4 | | |
|  | | FAC 1 | - | | - | | | - | - | | |
|  | | FAC 2 | 0.57 | | - | | | - | - | | |
|  | | FAC 3 | 0.30 | | 0.33 | | | - | - | | |
|  | | FAC 4 | 0.10 | | 0.28 | | | 0.01 | - | | |

Highest factor loadings for a given factor are bolded; Cross loadings are underlined.

# **Table S11.** Factor loadings of the confirmatory factor analysis of the collection of P-factor items

|  |  | | | | | | | |
| --- | --- | --- | --- | --- | --- | --- | --- | --- |
| **Item** | | **Content** | | **P-factor** | **FAC1** | **FAC2** | **FAC3** | **FAC4** |
| BAI_11 | | Feeling of choking | | 0.38 | 0.74 |  |  |  |
| BAI_15 | | Difficulty in breathing | | 0.33 | 0.74 |  |  |  |
| BAI_13 | | Shaky / unsteady | | 0.45 | 0.60 |  |  |  |
| BAI_19 | | Faint / lightheaded | | 0.33 | 0.58 |  |  |  |
| BAI_9 | | Terrified or afraid | | 0.52 | 0.58 |  |  |  |
| BAI_7 | | Heart pounding / racing | | 0.42 | 0.55 |  |  |  |
| BAI_4 | | Unable to relax | | 0.49 | 0.51 |  |  |  |
| BAI_10 | | Nervous | | 0.48 | 0.52 |  |  |  |
| BAI_8 | | Unsteady | | 0.50 | 0.48 |  |  |  |
| BAI_17 | | Scared | | 0.45 | 0.51 |  |  |  |
| BDI_1 | | Sadness | | 0.52 | 0.48 |  |  |  |
| BAI_12 | | Hands trembling | | 0.39 | 0.49 |  |  |  |
| BAI_14 | | Fear of losing control | | 0.55 | 0.45 |  |  |  |
| BAI_6 | | Dizzy or lightheaded | | 0.38 | 0.48 |  |  |  |
| BDI_20 | | Tiredness or Fatigue | | 0.48 | 0.50 |  |  |  |
| BAI_5 | | Fear of worst happening | | 0.50 | 0.48 |  |  |  |
| BDI_15 | | Loss of Energy | | 0.49 | 0.49 |  |  |  |
| BAI_3 | | Wobbliness in legs | | 0.43 | 0.39 |  |  |  |
| BDI_18 | | Changes in Appetite | | 0.39 | 0.39 |  |  |  |
| BDI_11 | | Agitation | | 0.34 | 0.40 |  |  |  |
| BDI_10 | | Crying | | 0.40 | 0.41 |  |  |  |
| BDI_17 | | Irritability | | 0.44 | 0.40 |  |  |  |
| BAI_1 | | Numbness or tingling | | 0.36 | 0.34 |  |  |  |
| BDI_4 | | Loss of pleasure | | 0.44 | 0.41 |  |  |  |
| BDI_9 | | Suicidal Thoughts | | 0.63 | 0.31 |  |  |  |
| BDI_6 | | Punishment Feeling | | 0.53 | 0.32 |  |  |  |
| BDI_21 | | Loss of Interest in Sex | | 0.27 | 0.35 |  |  |  |
| BAI_18 | | Indigestion | | 0.25 | 0.37 |  |  |  |
| BDI_19 | | Concentration Difficulties | | 0.53 | 0.35 |  |  |  |
| BDI_12 | | Loss of Interest | | 0.45 | 0.38 |  |  |  |
| BDI_7 | | Self-Dislike | | 0.53 | 0.36 | -0.07 |  |  |
| BDI_5 | | Guilty Feeling | | 0.55 | 0.30 |  |  |  |
| BDI_14 | | Worthlessness | | 0.65 | 0.31 |  |  |  |
| BDI_2 | | Pessimism | | 0.50 | 0.31 |  |  |  |
| BAI_21 | | Hot / cold sweats | | 0.35 | 0.29 |  |  |  |
| BDI_16 | | Changes in Sleep | | 0.39 | 0.28 |  |  |  |
| BDI_8 | | Self-Criticalness | | 0.48 | 0.29 |  |  |  |
| BAI_16 | | Fear of dying | | 0.34 | 0.28 |  |  |  |
| BDI_13 | | Indecisiveness | | 0.51 | 0.25 | -0.13 |  |  |
| BAI_20 | | Face flushed | | 0.31 | 0.23 |  |  |  |
| BAI_2 | | Feeling hot | | 0.32 | 0.12 |  |  |  |
| SPQ_16IOR | | When people talking to each other, wonder if they are talking about you | | 0.64 |  | 0.45 |  |  |
| SPQ_19IOR | | Feel that people are talking about you | | 0.62 |  | 0.52 |  |  |
| SPQ_7SUS | | Others have it in for me | | 0.70 |  | 0.19 |  |  |
| MSS2_9D | | Difficult to get my thoughts in order | | 0.81 |  | -0.30 |  |  |
| MSS2_11D | | No matter how hard I try, I can't organize my thoughts | | 0.79 |  | -0.31 |  |  |
| SPQ_3SUS | | Feel that other people have got it in for you | | 0.67 |  | 0.20 |  |  |
| SPQ_1SUS | | I am sure I am being talked about behind my back | | 0.56 |  | 0.38 |  |  |
| MSS2_13D | | Thoughts are almost always hard to follow | | 0.69 |  | -0.20 |  |  |
| MSS2_17D | | Thoughts and behaviors are almost always disorganized | | 0.76 |  | -0.35 |  |  |
| MSS2_19D | | Difficulty organizing what supposed to be doing | | 0.64 |  | -0.30 |  |  |
| MSS2_12D | | Thoughts hazy and unclear wish that could reach up and put them into place | | 0.80 |  | -0.31 |  |  |
| MSS2_16D | | Trouble following conversations with others | | 0.64 |  | -0.25 |  |  |
| SPQ_17IOR | | Feel that other people are watching you | | 0.53 |  | 0.49 | 0.28 |  |
| SPQ_15IOR | | When shopping get the feeling that other people are taking notice of you | | 0.52 |  | 0.50 | 0.24 |  |
| MSS2_18D | | Thoughts and behaviors feel random and unfocused | | 0.73 |  | -0.28 |  |  |
| MSS2_20D | | I often feel so mixed up that I have difficulty functioning | | 0.78 |  | -0.21 |  |  |
| MSS2_21D | | Have difficulty following what someone is saying | | 0.67 |  | -0.23 |  |  |
| SPQ_2SUS | | Concerned that friends or coworkers are not really loyal or trustworthy | | 0.53 |  | 0.27 |  |  |
| MSS2_15D | | People find my conversations to be confusing or hard to follow | | 0.55 |  | -0.11 |  | 0.29 |
| SPQ_4SUS | | I feel I have to be on my guard even with friends. | | 0.70 |  | 0.17 |  |  |
| CAPE_5 | | Conspiracy against you | | 0.59 |  | 0.21 | 0.24 |  |
| CAPE_11 | | People look at you oddly because of your appearance | | 0.65 |  | 0.24 |  |  |
| BDI_3 | | Past Failures | | 0.67 |  | -0.04 |  |  |
| SPQ_8SUS | | Keep an eye out to stop people from taking advantage | | 0.63 |  | 0.22 |  |  |
| CAPE_1 | | People drop hints about you or say things with a double meaning | | 0.47 |  | 0.26 | 0.31 |  |
| SPQ_5SUS | | Pick up hidden threats or put-downs from what people say or do | | 0.50 |  | 0.17 | 0.23 |  |
| MSS2_14D | | I find that I am very often confused about what is going on around me. | | 0.71 |  | -0.01 |  |  |
| SPQ_12IOR | | People drop hints about you or say things with a double meaning | | 0.49 |  | 0.37 |  |  |
| MSS_6P | | Messages in the way things are arranged, like furniture | | 0.24 |  |  | 0.79 |  |
| SPQ_13IOR | | Noticed a common event or object that seemed to be a special sign for you | | 0.25 |  |  | 0.73 |  |
| MSS_7P | | There are secret signs in the world if you know how to look for them | | 0.24 |  |  | 0.69 |  |
| SPQ_14IOR | | See special meanings in advertisements, shop windows, or in the way things are arranged around | | 0.24 |  |  | 0.71 |  |
| MSS_4P | | I have sometimes felt that strangers were reading my mind. | | 0.35 |  |  | 0.57 |  |
| CAPE_8 | | Communicate telepathically | | 0.26 |  |  | 0.58 |  |
| MSS_1P | | I believe that dreams have magical properties. | | 0.21 |  |  | 0.62 |  |
| MSS_3P | | Momentary feeling that someone's place has been taken by a look-alike. | | 0.31 |  |  | 0.54 |  |
| SPQ_9IOR | | Things on the TV or newspaper have a special meaning for you | | 0.25 |  |  | 0.65 |  |
| CAPE_12 | | Thoughts in your head are being taken away from you | | 0.43 |  |  | 0.50 |  |
| MSS_13P | | Experiences with seeing the future, ESP or a sixth sense | | 0.33 |  |  | 0.50 |  |
| CAPE_16 | | Under the control of some force or power other than yourself | | 0.50 |  |  | 0.46 |  |
| CAPE_2 | | Things in magazines or on TV were written especially for you | | 0.23 |  |  | 0.59 |  |
| MSS_14P | | Worry that someone or something is controlling my behavior | | 0.48 |  |  | 0.43 |  |
| MSS_8P | | Wonder if there is a small group of people controlling everyone else's behavior | | 0.31 |  |  | 0.46 |  |
| CAPE_6 | | Destined to be someone very important | | 0.15 |  |  | 0.58 |  |
| CAPE_10 | | Believe in the power of witchcraft, voodoo or the occult | | 0.25 |  |  | 0.50 |  |
| CAPE_7 | | You are a very special or unusual person | | 0.11 |  |  | 0.56 |  |
| CAPE_20 | | See objects, people or animals that other people cannot see | | 0.34 |  |  | 0.40 |  |
| CAPE_4 | | Being persecuted in some way | | 0.45 |  |  | 0.40 |  |
| CAPE_19 | | A double has taken the place of a family member, friend or acquaintance | | 0.25 |  |  | 0.41 |  |
| CAPE_17 | | Hear voices when you are alone | | 0.49 |  |  | 0.36 |  |
| MSS_2P | | Some people can make me aware of them just by thinking about me. | | 0.18 |  |  | 0.44 |  |
| CAPE_14 | | Thoughts so vivid that you were worried other people would hear them | | 0.49 |  |  | 0.38 |  |
| CAPE_3 | | Some people are not what they seem to be | | 0.45 |  | 0.25 | 0.36 |  |
| MSS_12P | | Feels like someone is touching me when no one is actually there | | 0.35 | 0.19 |  | 0.37 |  |
| CAPE_13 | | Thoughts in your head are not your own | | 0.58 |  |  | 0.32 |  |
| MSS_11P | | Wondered if my body was really my own | | 0.53 |  |  | 0.32 |  |
| CAPE_15 | | Hear your own thoughts being echoed back to you | | 0.47 |  |  | 0.34 |  |
| CAPE_18 | | Hear voices talking to each other when you are alone | | 0.45 |  |  | 0.31 |  |
| MSS_10P | | Hear people talking only to discover that there was no one there | | 0.49 |  |  | 0.32 |  |
| CAPE_9 | | Electrical devices such as computers can influence the way you think | | 0.23 |  |  | 0.34 |  |
| SPQ_10IOR | | People notice me when I go out for a meal or to see a film | | 0.44 |  | 0.48 | 0.30 |  |
| MSS_9P | | I often worry that other people are out to get me. | | 0.53 |  | 0.19 | 0.27 |  |
| SPQ_6SUS | | It is best not to let people know too much about you | | 0.50 |  |  | 0.23 |  |
| MSS2_2N | | Spending time with close friends and family is important | | 0.15 |  |  |  | 0.81 |
| MSS2_8N | | Important to have close relationships with other people | | 0.27 |  |  |  | 0.64 |
| MSS_17N | | Do not have many thoughts or emotions | | 0.03 |  |  |  | 0.67 |
| MSS2_6N | | Not interested in being emotionally close with others | | 0.22 |  |  |  | 0.68 |
| MSS_16N | | Emotions seemed flat regardless of what is going on around me | | 0.22 |  |  |  | 0.64 |
| MSS2_1N | | Having close friends is not as important as people say | | 0.08 |  |  |  | 0.58 |
| MSS_15N | | Noticed that I rarely feel strong positive or negative emotions | | 0.05 |  |  |  | 0.63 |
| MSS2_3N | | Rather be with another person than alone | | 0.21 |  |  |  | 0.49 |
| MSS2_4N | | Desire to be connected with other people | | -0.15 |  |  |  | 0.56 |
| MSS_19N | | Preferred to be disconnected from the world | | 0.45 |  |  |  | 0.47 |
| MSS_18N | | Few things have been exciting or interesting to me | | 0.39 |  |  |  | 0.57 |
| MSS2_5N | | Little interest in dating or being in a romantic relationship | | 0.18 |  |  |  | 0.32 |
| MSS2_7N | | Not many things ever really enjoyed doing | | 0.52 |  | -0.14 |  | 0.37 |
|  | |  | |  |  |  |  |  |
| FAC1: Affective Dysregulation; FAC2: Social/Cognitive Malfunction; FAC3: Positive schizotypy traits & experiences; FAC4: Negative schizotypy; Bifactor Confirmatory Factor Analysis was run utilizing the WLSMV estimator. | | | | | | | | |
|  | |  |  | | | | | |

|  | Explained Common Variance  (ECV SS) | Explained Common Variance  (ECV SG) | Explained Common Variance  (ECV GS) | Omega  ω | Omega-Hierarchical (Omega H) | H | Factor Determinacy (FD) |
| --- | --- | --- | --- | --- | --- | --- | --- |
| P-Factor | 0.52 | 0.52 | 0.52 | 0.98 | 0.78 | 0.98 | 0.99 |
| F1 Affective Dysreg. | 0.48 | 0.16 | 0.51 | 0.96 | 0.45 | 0.92 | 0.96 |
| F2 Social/Cognitive Malfunctioning | 0.16 | 0.06 | 0.78 | 0.96 | 0.01 | 0.77 | 0.93 |
| F3 Positive Schizotypy | 0.54 | 0.17 | 0.39 | 0.96 | 0.55 | 0.93 | 0.96 |
| F4 Negative Schizotypy | 0.78 | 0.09 | 0.21 | 0.89 | 0.77 | 0.89 | 0.95 |

# **Table S12.** Bifactor indices for the P-factor model

ECV: Explained Common Variance; ECV_SS: ECV of a specific factor relative to itself; ECV_SG: ECV of a specific factor relative to the general factor; ECV_GS: ECV of the general factor relative to a specific factor.

# **Table S13.** Factor loadings of the exploratory factor analysis of the collection of Positive Mental Health items (n = 1.181).

| **Item** | **Content** | | **F1** | | | **F2** | **F3** | | |  |  |
| --- | --- | --- | --- | --- | --- | --- | --- | --- | --- | --- | --- |
| WEWMBS_1 | Feeling optimistic about the future | | **0.48** | | | 0.19 | | | 0.12 |  |  |
| WEWMBS_2 | Feeling useful | | **0.46** | | | 0.37 | | | 0.06 |  |  |
| WEWMBS_3 | Feeling relaxed | | **0.70** | | | -0.07 | | | 0.00 |  |  |
| WEWMBS_4 | Feeling interested in other people | | **0.75** | | | -0.15 | | | -0.15 |  |  |
| WEWMBS_5 | Had energy to spare | | **0.75** | | | -0.13 | | | 0.03 |  |  |
| WEWMBS_6 | Dealing with problems well | | **0.58** | | | -0.08 | | | 0.31 |  |  |
| WEWMBS_7 | Thinking clearly | | **0.67** | | | -0.14 | | | 0.19 |  |  |
| WEWMBS_8 | Feeling good about self | | **0.61** | | | 0.27 | | | 0.02 |  |  |
| WEWMBS_9 | Feeling close to other people | | **0.85** | | | -0.03 | | | -0.20 |  |  |
| WEWMBS_10 | Feeling confident | | **0.55** | | | 0.23 | | | 0.12 |  |  |
| WEWMBS_11 | Make up own mind about things | | **0.33** | | | 0.05 | | | 0.29 |  |  |
| WEWMBS_12 | Feeling loved | | **0.67** | | | 0.11 | | | -0.20 |  |  |
| WEWMBS_13 | Interested in new things | | **0.59** | | | -0.09 | | | 0.07 |  |  |
| WEWMBS_14 | Feeling cheerful | | **0.84** | | | -0.04 | | | -0.02 |  |  |
| CDRISC_1 | Able to adapt when changes occur | | 0.05 | | | **0.73** | | | -0.03 |  |  |
| CDRISC_2 | Able to deal with whatever comes | | 0.14 | | | **0.62** | | | 0.02 |  |  |
| CDRISC_3 | Try to see the humorous side of things when faced with problems | | -0.27 | | | **0.84** | | | -0.09 |  |  |
| CDRISC_4 | Cope with stress makes stronger | | -0.27 | | | **0.84** | | | 0.05 |  |  |
| CDRISC_5 | Bounce back after illness. injury or other hardships | | 0.04 | | | **0.58** | | | 0.05 |  |  |
| CDRISC_6 | Able to achieve goals. even if there are obstacles | | 0.16 | | | **0.54** | | | 0.06 |  |  |
| CDRISC_7 | Stay focused and think clearly | | -0.11 | | | **0.91** | | | -0.10 |  |  |
| CDRISC_8 | Not easily discouraged by failure | | 0.14 | | | **0.43** | | | -0.01 |  |  |
| CDRISC_9 | Strong person when dealing with life’s challenges and difficulties | | 0.14 | | | **0.65** | | | 0.03 |  |  |
| CDRISC_10 | Able to handle unpleasant or painful feelings | | 0.06 | | | **0.76** | | | -0.03 |  |  |
| RSES_1 | Satisfied with self | | 0.00 | | | -0.09 | | | **0.72** |  |  |
| RSES_2 | Think one is not good at all | | -0.14 | | | 0.02 | | | **0.84** |  |  |
| RSES_3 | Think one has good qualities | | 0.12 | | | -0.04 | | | **0.57** |  |  |
| RSES_4 | Able to do things as well as most | | -0.07 | | | -0.04 | | | **0.70** |  |  |
| RSES_5 | Feel one doesn’t have much to be proud of | | 0.05 | | | -0.11 | | | **0.55** |  |  |
| RSES_6 | Feel useless at times | | -0.08 | | | 0.23 | | | **0.62** |  |  |
| RSES_7 | Feeling a person of worth | | -0.05 | | | -0.10 | | | **0.64** |  |  |
| RSES_8 | Could have more respect self | | 0.05 | | | 0.16 | | | **0.52** |  |  |
| RSES_9 | Inclined to think that one is a failure | | -0.11 | | | 0.07 | | | **0.79** |  |  |
| RSES_10 | Take positive attitude toward self | | 0.00 | | | 0.02 | | | **0.60** |  |  |
| Inter-factor correlations resulting from EFA | | | | | | | | | | | |
|  | | |  | | F1 | F2 | | | F3 | | |
|  | | | FAC 1 | | - | - | | | - | | |
|  | | | FAC 2 | | .60 | - | | | - | | |
|  | | | FAC 3 | | .59 | .59 | | | - | | |

Highest factor loadings for a given factor are bolded; Cross-loadings are underlined.

# **Table S14.** Factor loadings of the confirmatory factor analysis of the collection of the Positive Mental Health items (n=1181).

| Item | Content | FG  General | F1  Self-steem | F2  Well-being | F3  Resilience |
| --- | --- | --- | --- | --- | --- |
| RSES_1 | Satisfied with self | 0.67 | 0.44 |  |  |
| RSES_2 | Think one is not good at all | 0.80 | 0.02 |  |  |
| RSES_3 | Think one has good qualities | 0.42 | 0.74 |  |  |
| RSES_4 | Able to do things as well as most | 0.51 | 0.67 |  |  |
| RSES_5 | Feel one does not have much to be proud of | 0.64 | 0.13 |  |  |
| RSES_6 | Feel useless at times | 0.79 | -0.01 |  |  |
| RSES_7 | Feeling a person of worth | 0.59 | 0.59 |  |  |
| RSES_8 | Could have more respect self | 0.57 | 0.05 |  |  |
| RSES_9 | Inclined to think that one is a failure | 0.79 | 0.15 |  |  |
| RSES_10 | Take positive attitude toward self | 0.71 | 0.42 |  |  |
| WEWMBS_1 | Feeling optimistic about the future | 0.68 |  | 0.25 |  |
| WEWMBS_2 | Feeling useful | 0.78 |  | 0.22 |  |
| WEWMBS_3 | Feeling relaxed | 0.47 |  | 0.44 |  |
| WEWMBS_4 | Feeling interested in other people | 0.27 |  | 0.60 |  |
| WEWMBS_5 | Had energy to spare | 0.48 |  | 0.48 |  |
| WEWMBS_6 | Dealing with problems well | 0.65 |  | 0.44 |  |
| WEWMBS_7 | Thinking clearly | 0.56 |  | 0.49 |  |
| WEWMBS_8 | Feeling good about self | 0.77 |  | 0.36 |  |
| WEWMBS_9 | Feeling close to other people | 0.41 |  | 0.66 |  |
| WEWMBS_10 | Feeling confident | 0.78 |  | 0.33 |  |
| WEWMBS_11 | Make up own mind about things | 0.58 |  | 0.22 |  |
| WEWMBS_12 | Feeling loved | 0.45 |  | 0.46 |  |
| WEWMBS_13 | Interested in new things | 0.43 |  | 0.36 |  |
| WEWMBS_14 | Feeling cheerful | 0.59 |  | 0.57 |  |
| CDRISC_1 | Able to adapt when changes occur | 0.51 |  |  | 0.41 |
| CDRISC_2 | Able to deal with whatever comes | 0.55 |  |  | 0.58 |
| CDRISC_3 | Try to see the humorous side of things when faced with problems | 0.53 |  |  | 0.30 |
| CDRISC_4 | Cope with stress makes stronger | 0.47 |  |  | 0.43 |
| CDRISC_5 | Bounce back after illness. injury or other hardships | 0.39 |  |  | 0.29 |
| CDRISC_6 | Able to achieve goals. even if there are obstacles | 0.65 |  |  | 0.37 |
| CDRISC_7 | Stay focused and think clearly | 0.37 |  |  | 0.34 |
| CDRISC_8 | Not easily discouraged by failure | 0.61 |  |  | 0.25 |
| CDRISC_9 | Strong person when dealing with life’s challenges and difficulties | 0.60 |  |  | 0.54 |
| CDRISC_10 | Able to handle unpleasant or painful feelings | 0.50 |  |  | 0.32 |

Bifactor Confirmatory Factor Analysis was run utilizing the WLSMV estimator.

|  |  |  |  |  |  |  |  |  |
| --- | --- | --- | --- | --- | --- | --- | --- | --- |
|  | Explained Common Variance  (ECV SS) | Explained Common Variance  (ECV SG) | Explained Common Variance  (ECV GS) | Omega  ω | Omega-Hierarchical (Omega H) | | H | Factor Determinacy (FD) |
| Positive Mental Health | 0.66 | 0.66 | 0.66 | 0.96 | 0.84 | | 0.96 | 0.96 |
| F1 Well-being | 0.29 | 0.10 | 0.71 | 0.93 | 0.18 | | 0.75 | 0.90 |
| F2 Resilience | 0.36 | 0.15 | 0.64 | 0.94 | 0.33 | | 0.79 | 0.88 |
| F3 Self-steem | 0.36 | 0.09 | 0.64 | 0.88 | 0.31 | | 0.67 | 0.84 |

# **Table S15.** Bifactor indices for the Positive Mental Health model

ECV: Explained Common Variance; ECV_SS: ECV of a specific factor relative to itself; ECV_SG: ECV of a specific factor relative to the general factor; ECV_GS: ECV of the general factor relative to a specific factor.

# **Table S16**. Correlations between the Early Exposome, its specific dimensions and the P-factor, Positive Mental Health and functioning (*n* = 1181).

|  | Early Exposome | F1  Positive  Experiences | F2  Paternal Adversity | F3  Maternal  Adversity | F4  Role Reversal |
| --- | --- | --- | --- | --- | --- |
| P-factor | **.42^**^** | -.18^**^ | .08^**^ | .13^**^ | .04 |
| Positive Mental Health | **-.38^**^** | .17^**^ | -.06^*^ | -.07^*^ | .05 |
| Functioning | ***-.50^**^*** | .21^**^ | -.12^**^ | -.06^*^ | .02 |

Moderate correlations are in bold (*r* ≥ .30), large correlations (*r* ≥ .50) are in bold and italics (Cohen, 1988).

# **Table S17.** Correlations between the Early Exposome, its specific dimensions and psychopathology and positive mental health individual measures (*n* = 1181).

|  |  |  |  |  |  |
| --- | --- | --- | --- | --- | --- |
|  | Early Exposome | F1  Positive Experiences | F2  Paternal Adversity | F3  Maternal Adversity | F4  Role Reversal |
| Psychosis Extended Phenotype |  |  |  |  |  |
| MSS Positive | .18** | -.03 | .02 | .10** | .12** |
| MSS Negative | .23** | -.16** | .01 | .04 | -.00 |
| MSS Disorganized | **.36**** | -.15** | .07* | .12** | .02 |
| CAPE Positive | .23** | -.06* | .06* | .08** | .09** |
| Suspiciousness | **.32**** | -.18** | .06* | .09** | .04 |
| Ideas of Reference | .19** | -.04 | .06* | .08** | .05 |
| Affective Dysregulation |  |  |  |  |  |
| Depression | **.42**** | -.16** | .05 | .07* | .04 |
| Anxiety | **.34**** | -.12** | .01 | .05 | .07* |
| Positive Mental Health |  |  |  |  |  |
| Well-being | **-.41**** | .16** | -.06* | -.03 | .05 |
| Resilience | -.27** | .16** | -.10** | -.04 | .11** |
| Self-esteem | **-.35**** | .17** | -.04 | -.03 | .04 |

Moderate correlations are in bold (*r* ≥ .30), large correlations (*r* ≥ .50) are in bold and italics (Cohen, 1988).

**Supplementary Methods**

## *Iterative exploratory factor analyses for environmental data reduction*

As outlined by Moore et al. (2022), the data reduction analysis involves multiple iterations of the following process: (i) estimation of a mixed correlation matrix for all variables; (ii) extraction of a scree plot of descending eigenvalues and a parallel analysis from this matrix to determine the number of factors to retain in the subsequent EFA; (iii) estimation of an EFA model using oblimin rotation and unweighted least squares (ULS) factoring; and (iv) interpretation of the solution. Variables with loadings below .30 across all factors were removed. As described by Moore et al., if a factor exclusively consisted of items from the same measure, the composite score for that measure or subscale was calculated, and the individual items subsequently removed. Likewise, if items from a measure or subscale loaded onto a factor with similar factor loadings without significant cross-loadings onto other factors, individual items were replaced with their corresponding composite score.

This process was repeated until all redundant or irrelevant variables were eliminated, and the retained variables in the final dataset loaded at .30 or higher on a factor. Once this criterion was met, a final EFA analysis implementing iterated target rotation (ITR; Moore, Reise, Depaoli, & Haviland, 2015) was performed. ITR is a refined rotation algorithm that improves upon basic rotation methods (such as oblimin or promax) by using an iterative procedure aimed at achieving orthogonal factors, thereby enhancing interpretability and replicability (Moore, 2013).

In the ITR process, an initial factor solution, also employing oblimin rotation and ULS factoring, was obtained. This solution is then converted into a target matrix, with the estimation threshold set to .20 (Moore et al., 2022), meaning that all loadings above this threshold are freely estimated, while those below are fixed at zero. Subsequently, the target matrix was iteratively refined until convergence (for more details on the process, see Moore et al., 2013, 2015).

## *Bifactor modeling of the early exposome*

The factorial structure obtained in the final EFA employing ITR served as the basis for defining a Bifactor Confirmatory Factor Analysis (BCFA). Note that this model is quasi-confirmatory in nature, as its primary aim is to derive factor scores rather than to strictly confirm a pre-specified model (Moore et al., 2022; Reise, 2012). Factor analyses were performed with R (package psych; Revelle, 2020).

The BCFA was configured such that all items loaded on both a general factor and their specific factors, with cross-loadings also accounted for. Specific factors were not allowed to correlate with each other, or with the general factor. The analysis was conducted in MPlus 8 (Muthén & Muthén, 2017), using the weighted least squares mean- and variance-adjusted estimator (WLSMV) and specifying categorical and continuous variables.

Global fit of the BCFA model was assessed based on the comparative fit index (CFI), Tucker-Lewis index (TLI), root mean square error of approximation (RMSEA), and standardized root mean residual (SRMR). According to the criteria outlined by Brown (2015) and Schreiber, Nora, Stage, Barlow, and King (2006), CFI and TLI values of ≥ .90, RMSEA ≤ .08, and SRMR ≤ .10 indicate a good model fit, while CFI and TLI ≥ .95, RMSEA ≤ .06, and SRMR ≤ .08 represent an excellent fit. Additional bifactor model-based reliability indices such as explained common variance (ECV), factor determinacy, H index and omega (ω) were calculated with R package “Bifactor Indices Calculator” (Dueber, 2017). According to Gorsuch (1983), factor determinacy should be higher than .90 for the factor score estimates to be reliable. Based on the criteria from Rodriguez, Reise and Haviland (2016) and Dueber and Toland (2023), ω > .70 indicates adequate internal reliability, while H > .80 reflects good construct reliability.

## *Environmental measures*

The Childhood Trauma Questionnaire-Brief (CTQ-B; Bernstein et al., 2003) is a 28-item self-report that assesses the severity of sexual abuse, physical abuse, emotional abuse, physical neglect and emotional neglect, before the age of 18 years old.

The Childhood Experiences of Care and Abuse Questionnaire-3 Brief (CECA.Q-3; Li, Cassis, D’Arcy, Low & Meng, 2020) is a self-report questionnaire adapted from the CECA.Q interview (Bifulco, Bernazzani, Moran & Jacobs, 2005). We administered the following subscales to assess content less represented in the CTQ-B: support, discord, violence, role reversal, paternal antipathy, maternal antipathy, paternal psychological abuse and maternal psychological abuse (48 items in total).

The Parental Bonding Instrument (PBI; Parker, Tupling & Brown., 1979) is a 50-item questionnaire that assesses perceptions of maternal (25 items) and paternal (25 items) **care** (warmth and affection) and **overprotection** (control and intrusion) before the age of 16 years old.

The Benevolent Childhood Experiences Scale (BCE; Narayan, Rivera, Bernstein, Harris & Lieberman, 2018) includes 10 items assessing positive childhood experiences such as having a caregiver you felt safe with, enjoying school, and having opportunities for fun.

The Positive Childhood Experiences Scale (PCE; Bethell, Jones, Gombojav, Linkenbach & Sege., 2019) is a 7-item measure complementary to the BCE that assesses supportive experiences in childhood such as having friends and a sense of belonging in school.

Additionally, 4 items assessing family-level protective factors were included. These items assessed the state of the home, stimulation, happiness, and chaos. These items were derived by Crush et al. (2018) from the Coder's Impression Inventory, which is based on the Home Observation for Measurement of the Environment (HOME; Bradley & Caldwell, 1977) and the University of Washington Parenting Clinic Questionnaire (Parent–Child Observations) (Webster-Stratton, 1998).

## *Phenotypic measures*

## *Psychopathology measures*

The Multidimensional Schizotypy Scale-Brief (MSS-B; Gross, Kwapil, Raulin, Silvia & Barrantes-Vidal, 2018) was employed to assess the positive, negative, and disorganized dimensions of schizotypy. The Community Assessment of Psychic Experiences (CAPE; Stefanis et al., 2002) assessed psychotic-like experiences (PLE). A technical issue in the data collection software resulted in 4% of participants having missing data for items on the disorganized MSS subscale and the PLE. Thus, multiple imputation was conducted in accordance with Rubin's (1987) method. Trait paranoia was assessed using the Ideas of Reference and Suspiciousness subscales of the Schizotypal Personality Questionnaire (SPQ; Raine, 1991). Depressive symptoms were assessed using the Beck Depression Inventory (BDI; Beck, Ward, Mendelson, Mock & Erbaugh., 1979), a 21-item self-report measure that evaluates the severity symptoms experienced over the past week. Anxiety symptoms were measured with the Beck Anxiety Inventory (BAI; Beck, Epstein, Brown & Steer, 1988), a 21-item self-report questionnaire designed to assess the intensity of anxiety symptoms.

### Positive mental health measures

The Rosenberg Self-Esteem Scale (RSES; Rosenberg, 1965) is a 10-item questionnaire that measures global self-esteem, reflecting an individual’s overall evaluation of their self-worth. The Connor-Davidson Resilience Scale (CD-RISC; Campbell-Sills & Stein, 2007) is a 10-item scale designed to assess resilience, or the ability to cope with stress and adversity effectively, and the Warwick-Edinburgh Mental Wellbeing Scale (WEMWBS; Tennant et al., 2007) is a 14-item measure that evaluates mental well-being, focusing on positive aspects of mental health such as optimism, energy, and positive relationships.

### Functioning

The Social Functioning Questionnaire (Tyrer et al., 2005) was used. This 8-item self-report scale is rated on a four-point scale (0-3), and addresses both general (e.g., “I complete my tasks at work and home satisfactorily”) and social (e.g., “I have difficulties in getting and keeping close relationships”) functioning. Higher scores indicate better functioning.

# **Supplementary References**

1. Beck, A. T., Ward, C. H., Mendelson, M., Mock, J., & Erbaugh, J. (1979). *Beck Depression Inventory*. Psychological Corporation.
2. Beck, A. T., Epstein, N., Brown, G., & Steer, R. A. (1988). An inventory for measuring clinical anxiety: Psychometric properties. *Journal of Consulting and Clinical Psychology, 56*(6), 893–897. doi: 10.1037/0022-006X.56.6.893
3. Bernstein, D. P., Stein, J. A., Newcomb, M. D., Walker, E., Pogge, D., Ahluvalia, T., … Zule, W. (2003). Development and validation of a brief screening version of the Childhood Trauma Questionnaire. *Child Abuse & Neglect*, *27*(2), 169–190. doi: 10.1016/s0145-2134(02)00541-0
4. Bethell, C., Jones, J., Gombojav, N., Linkenbach, J., & Sege, R. (2019). Positive childhood experiences and adult mental and relational health in a statewide sample: associations across adverse childhood experiences levels. *JAMA Pediatrics*, *173*(11), e193007. doi: 10.1001/jamapediatrics.2019.3007
5. Bifulco, A., Bernazzani, O., Moran, P. M., & Jacobs, C. (2005). The childhood experience of care and abuse questionnaire (CECA.Q): Validation in a community series. *British Journal of Clinical Psychology, 44*(4), 563–581. doi:10.1348/014466505X35344
6. Bradley, R. H., & Caldwell, B. M. (1977). Home observation for measurement of the environment: A validation study of screening efficiency. *American Journal of Mental Deficiency, 81*(5), 417–420.
7. Brown, T. A. (2015). *Confirmatory factor analysis for applied research* (2nd ed.). Guilford Press.
8. Campbell-Sills, L., & Stein, M. B. (2007). Psychometric analysis and refinement of the Connor-Davidson Resilience Scale (CD-RISC): Validation of a 10-item measure of resilience. *Journal of Traumatic Stress, 20*(6), 1019–1028. doi: 10.1002/jts.20271
9. Crush, E., Arseneault, L., Moffitt, T. E., Danese, A., Caspi, A., Jaffee, S. R., … Fisher, H. L. (2018). Protective factors for psychotic experiences amongst adolescents exposed to multiple forms of victimization. *Journal of Psychiatric Research*, *104*, 32–38. doi: 10.1016/j.jpsychires.2018.06.011
10. Dueber, D. M. (2017). *Bifactor indices calculator* [R package]. https://CRAN.R-project.org/package=BifactorIndicesCalculator
11. Dueber, D. M., & Toland, M. D. (2023). A bifactor approach to subscore assessment. *Psychological Methods, 28*(1), 222–241. doi: 10.1037/met0000459
12. Gorsuch, R. L. (1983). *Factor analysis*. L. Erlbaum Associates.
13. Gross, G. M., Kwapil, T. R., Raulin, M. L., Silvia, P. J. & Barrantes-Vidal, N. (2018). The multidimensional schizotypy scale-brief: Scale development and psychometric properties. *Psychiatry Research, 261*, 7-13. doi: 10.1016/j.psychres.2017.12.033
14. Li, M., Cassis, T., D'Arcy, C., Low, N., & Meng, X. (2020). Development and validation of a brief form of the childhood adversities questionnaire among a population of mood disorders. *Journal of Interpersonal Violence*, 37(3-4), 1903–1928. doi: 10.1177/0886260520933037
15. Moore, T. M. (2013). *Iteration of target matrices in exploratory factor analysis* (Doctoral dissertation, University of California, Los Angeles). Retrieved from: https://search.proquest.com/docview/1499086987?accountid=14515
16. Moore, T. M., Reise, S. P., Depaoli, S., & Haviland, M. G. (2015). Iteration of partially specified target matrices: Applications in exploratory and Bayesian confirmatory factor analysis. *Multivariate Behavioral Research*, *50*(2), 149–161. doi: 10.1080/00273171.2014.973990
17. Moore, T. M., Visoki, E., Argabright, S. T., Didomenico, G. E., Sotelo, I., Wortzel, J. D., … Barzilay, R. (2022). Modeling environment through a general exposome factor in two independent adolescent cohorts. *Exposome*, *2*(1), osac010. doi:10.1093/exposome/osac010
18. Muthén, L. K., & Muthén, B. O. (2017). *Mplus: Statistical analysis with latent variables: User’s guide* (Version 8).
19. Narayan, A. J., Rivera, L. M., Bernstein, R. E., Harris, W. W., & Lieberman, A. F. (2018). Positive childhood experiences predict less psychopathology and stress in pregnant women with childhood adversity: A pilot study of the benevolent childhood experiences (BCEs) scale. *Child Abuse & Neglect, 78*, 19–30. doi:10.1016/j.chiabu.2017.09.022
20. Parker, G., Tupling, H. & Brown, L. (1979). A parental Bonding Instrument. *British Journal of Medical Psychology, 52,* 1-10. doi: 10.1037/t06510-000
21. Raine, A. (1991). The SPQ: A scale for the assessment of schizotypal personality based on DSM-III-R criteria. *Schizophrenia Bulletin, 17*(4), 555-564. doi:10.1093/schbul/17.4.555
22. Reise, S. P. (2012). The rediscovery of bifactor measurement models. *Multivariate Behavioral Research*, *47*(5), 667–696. doi:10.1080/00273171.2012.715555
23. Revelle, W. (2020). *Psych: Procedures for personality and psychological research* (Version 2020) [R package]. https://CRAN.R-project.org/package=psych
24. Rodríguez, A., Reise, S. P., & Haviland, M. G. (2016). Evaluating bifactor models: Calculating and interpreting statistical indices. *Psychological Methods*, *21*(2), 137–150. doi: 10.1037/met0000045
25. Rosenberg, M. (1965). *Society and the adolescent self-image.* Princeton University Press. doi:10.1515/9781400876136
26. Rubin, D.B. (1987). *Multiple Imputation for Nonresponse in Surveys*. John Wiley & Sons Inc. doi:10.1002/9780470316696
27. Schreiber, J. B., Nora, A., Stage, F. K., Barlow, E. A., & King, J. (2006). Reporting structural equation modeling and confirmatory factor analysis results: A review. *The Journal of Educational Research, 99*(6), 323–338. doi: 10.3200/JOER.99.6.323-338
28. Stefanis, N. C., Hanssen, M., Smirnis, N. K., Avramopoulos, D. A., Evdokimidis, I. K., Stefanis, C. N., Verdoux, H., & van Os, J. (2002). Evidence that three dimensions of psychosis have a distribution in the general population. *Psychological Medicine, 32*(2), 347–358. doi: 10.1017/S0033291701005141
29. Tennant, R., Hiller, L., Fishwick, R., Platt, S., Joseph, S., Weich, S., … Stewart-Brown, S. (2007). The Warwick-Edinburgh Mental Well-being Scale (WEMWBS): development and UK validation. *Health and Quality of Life Outcomes*, *5(*63), 1-13.  [doi: 10.1186/1477-7525-5-63](https://doi.org/10.1186/1477-7525-5-63)
30. Tyrer, P., Nur, U., Crawford, M., Karlsen, S., MacLean, C., Rao, B., & Johnson, T. (2005). The Social Functioning Questionnaire: a rapid and robust measure of perceived functioning. *International Journal of Social Psychiatry*, *51*(3), 265-275.  [doi: 10.1177/0020764005057391](https://doi.org/10.1177/0020764005057391)
31. Webster-Stratton, C. (1998). Preventing conduct problems in Head Start children: Strengthening parenting competencies. *Journal of Consulting and Clinical Psychology*, *66*(5), 715–730. doi:10.1037/0022-006X.66.5.715
